# Palladium-Catalyzed Selective Carbonylation Reactions of *Ortho*-Phenylene Dihalides with Bifunctional *N*,*O*-Nucleophiles

**DOI:** 10.3390/molecules29235620

**Published:** 2024-11-27

**Authors:** Fanni Bede, Attila Takács, László Kollár, Péter Pongrácz

**Affiliations:** 1 Department of General and Inorganic Chemistry, University of Pécs, Ifjúság u. 6., H-7624 Pécs, Hungary; fanni.bede@gmail.com (F.B.); kollar@gamma.ttk.pte.hu (L.K.); 2 HUN-REN-PTE Research Group for Selective Chemical Syntheses, Ifjúság u. 6., H-7624 Pécs, Hungary; takacsattila@gamma.ttk.pte.hu; 3 János Szentágothai Research Centre, University of Pécs, Ifjúság u. 20., H-7624 Pécs, Hungary

**Keywords:** carbonylation, palladium, homogeneous catalysis, amino alcohol, carbon monoxide, bifunctional nucleophile, aryl halide, amide alcohol, amide-ester, intramolecular carbonylation

## Abstract

Palladium-catalyzed carbonylation reactions of *ortho*-phenylene dihalides were studied using aminoethanols as heterobifunctional *N*,*O*-nucleophiles. The activity of aryl-iodide and -bromide as well as the chemoselective transformation of amine and hydroxyl functionalities were studied systematically under carbonylation conditions. Aminocarbonylation can be selectively realized under optimized conditions, enabling the formation of amide alcohols, and the challenging alkoxycarbonylation can also be proved feasible, enabling amide-ester production. Intramolecular double carbonylation reaction can be achieved using 1,2-diiodobenzene and amino alcohols featuring secondary amine groups, giving oxazocine derivatives. Useful reaction scope with various amino alcohols was performed with good isolated yields of the targeted compounds. Intramolecular C-O coupling of amide alcohols possessing bromo substituent in adjacent *ortho* position is also demonstrated as a potential next step in benzoxazepine heterocycle formation.

## 1. Introduction

Transition metal-catalyzed carbonylation reactions have gained significant attention in organic chemistry in recent decades due to their versatility and potential for use in the synthesis of a variety of important compounds, including pharmaceuticals and other fine chemicals. The reaction can be classified by the type of the substrate and the nucleophile. The transformation of olefins and alkynes [1,2,3] (hydrocarbonylations) as well as aryl [4,5,6] and alkenyl halides [7] (carbonylations) can be performed in the presence of various nucleophiles, such as alcohols, phenols [8], amines [9], thiols [10,11], or water [12,13]. Most of the preliminary studies focus on simple nucleophiles, while the application of multifunctional derivatives (more than one nucleophilic group) is neglected. However, some results clearly demonstrate that different nucleophilic groups can be selectively transformed by the proper choice of catalysts and reaction conditions.

The carbonylation reactions of *para*- and *meta*-aminophenols were studied thoroughly to achieve the regioselective transformation of styrene [14] and alkynes [15]. Ligand- and base-controlled chemoselective alkoxy- and aminocarbonylation are also presented with iodoarenes [16]. Additionally, the origin of chemoselectivity is explored in detail with theoretical calculations [17]. Aromatic and aliphatic diamines were also used in carbonylation reactions, which proved to be an efficient synthetic strategy to prepare dicarboxamides [18]. Besides some sporadic results [19], our research group investigated the carbonylation of iodoalkenes [20] and iodoaromatics in the presence of heterobifunctional ethanolamines as *N*,*O*-nucleophiles, in which the selective synthesis of amide alcohols and amide-esters was realized [21], controlled by the base and substrate ratio.

These references demonstrate that homo- and heterobifunctional nucleophiles can be used effectively in carbonylation reactions to synthesize a variety of compounds with high yield and selectivity. The range of synthetic possibilities of *N*,*O*-nucleophiles can be extended by the application of dihalogenated substrates. It is known that the aminocarbonylation of *ortho*-diiodo- and dibromobenzene is an excellent method to prepare various phthalimides [22,23,24,25], and 2-iodobenzyl bromide is also a capable substrate of carbonylation reactions [26], but a detailed investigation of dihalogenated substrates with bifunctional nucleophiles is highly desirable. Herein, we demonstrate the selective transformation of *ortho*-phenylene dihalides with *N*,*O*-nucleophiles to the corresponding amide and ester derivatives.

## 2. Results and Discussion

We sought to systematically explore the carbonylation reactions of 2-bromoiodobenzene (**1a**), 1,2-dibromobenzene (**1b**), and 1,2-diiodobenzene (**1c**) in the presence of various amino alcohols as *N*,*O*-heterobifunctional nucleophiles. We initiated our study by conducting the reaction of **1a** with 2-aminoethanol (**2a**) under typical carbonylation conditions (see Table 1, entry 1), obtaining full conversion and a mixture of products. Not surprisingly, the aminocarbonylation of the iodoaromatic structural element was highly dominated, providing the 2-bromo amide alcohol (**3a**) and the imide alcohol (**4a**) with 73% and 12% selectivity, respectively. Additional side products were also formed in varying quantities during the optimization reactions: 2-(2-bromophenyl)-4,5-dihydrooxazole (**5a**) and two further products, 2-bromo-*N*,*N*-dimethylbenzamide and 2-bromo-*N*,*N*-diethylbenzamide, as well as the *N*-nucleophiles stemming from DMF and Et_3_N, respectively [27,28]. Contrary to our previous studies with iodobenzene [21], the alkoxycarbonylation reaction was completely scaled back with the substrate–nucleophile ratio at 1:1; thus, no amide-ester (**6a**) or imide-ester (**7a**) derivatives were detected.

To increase the selectivity toward the targeted amide alcohol product, additional bases, ligands, and solvents were introduced into the reaction (entries 2–3, 6–7, and 8–9, respectively). Unfortunately, the formation of **3a** was not improved significantly, but under high-pressure conditions (entries 5 and 12), some increase in selectivity was obtained. By lowering the temperature to 75 and 50 °C (entries 10 and 11), full conversions were also achieved with the Xantphos ligand after 48 h of reaction with comparable chemoselectivity, while the side product formation is considerably suppressed. When performing the reactions in the absence of phosphine ligands, (entry 13) there was comparable chemoselectivity, but no complete conversion was obtained after 48 h. Interestingly, under 50 °C, some alkoxycarbonylated product (**6a**) was also detected. As expected, a further increase in amide-ester selectivity could be achieved by raising the nucleophile amount (entries 15–17), reaching 83% selectivity.

Based on the above-discussed results, analogous carbonylation reactions of 2-bromoiodobenzene (**1a**) were conducted with 2-(methylamino)ethanol (**2b**) as secondary amine. Reasonably, the imide and oxazole derivatives cannot be formed in this case; thus, under typical conditions and higher temperatures, the amide alcohol (**3b**) was formed with excellent conversion and selectivity (Table 2, entry 1). Similarly to earlier observations, TPP was not a suitable ligand for esterification even under an increased nucleophile ratio, but Xantphos provided the amide-ester derivative (**6b**) with moderate selectivity (see entries 2 and 4, respectively). Higher pressure favors double carbonylation, and also good chemoselectivity can be achieved under lower temperatures with extended reaction time (entry 6).

As expected, the iodo-arene and the *N*-nucleophilic group are easily involved in the carbonylation reaction, forming the amide functionality. The *ortho*-positioned aryl bromide remained unchanged in most cases and only occurred in imide formation. The OH-group also proved to be a capable nucleophile in the carbonylation reaction studied, but we suppose that the alkoxycarbonylation was always preceded by an aminocarbonylation step (no ester-amine can be detected). After many attempts, we found the reaction conditions for the synthesis of amide alcohol and amide-ester derivatives with acceptable selectivity using primary and secondary amino alcohols.

Considering that aryl iodides are very reactive in the studied aminocarbonylation reaction, we were curious about the activity of 1,2-dibromobenzene using the model heterobifunctional *N*,*O*-nucleophiles. Under the previously selected carbonylation conditions (Pd(OAc)_2_, TPP, Et_3_N, DMF, 1 bar CO, 100 °C), the catalyst system remained inactive (Table 3, entries 1 and 2). Many attempts were made to find better conditions, some selected experiments can be found below in Table 3, and the detailed ligand effect was also investigated (see Supplementary Materials). When changing the ligand to bidentate Xantphos 53% conversion, a mixture of products were observed after 24 h of reaction time, containing the previously identified compounds (entry 3). The amide alcohol (**3a**) and the imide (**4a**) were formed dominantly beside some known side products (dimethyl-, diethyl-benzamide, and oxazole derivative (**5a**)). A double amount of ligand increased the side product formation, while target compound selectivity remained low (entry 3). Some activity increases can be achieved by doubling the amount of the ligand (entry 4), but unfortunately, the selectivity for the desired compound was still low. Good chemoselectivity can be achieved at lower temperatures besides significant activity loss, and under 50 °C, the reaction did not occur (entries 5 and 6, respectively). Likewise, low activity and selectivity were obtained with two equivalents of the substrate (entry 7) without detecting any traces of amide-ester derivative (**6a**).

Performing the reaction with 2-(methylamino)ethanol yielded much higher conversion and good selectivity for the amide alcohol (**3b**) under initial conditions using Xantphos ligand, which can be explained by the prohibited formation of imide and oxazole derivatives. This was the first time that we realized the presence of the eight-membered heterocyclic oxazocine derivate (**8b**) in the reaction mixture. The catalyst’s performance cannot be improved by temperature increase, using other precursors, or ligands, (entries 9, 10, 12, respectively), and complete activity loss was observed under high-pressure conditions (entry 11). To initiate the alkoxycarbonylation reaction, a substrate–nucleophile ratio of 2 to 1 was applied, but only traces of amide-ester derivative were detected under various conditions (see Table 3, entries 12–15 and Supplementary Materials for ligand effect investigation). As expected, the aryl-bromide is much less reactive in the studied carbonylation reactions, especially with *O*-nucleophiles. Only traces of ester-derivatives were detected, and interestingly, alkoxycarbonylation and intramolecular cycloalkoxycarbonylation were observed to a similar extent.

**Figure 1:**
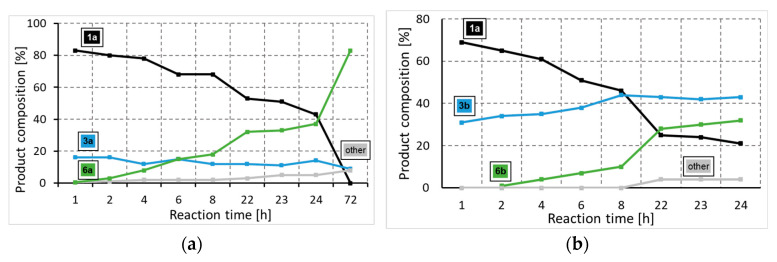
Time plot of carbonylation reactions of 2-iodobromobenzene with 2-aminoethanol (**a**) or 2-(methylamino)ethanol (**b**). (**a**) Reaction conditions: Pd(OAc)_2_ (0.01 mmol); Xantphos (0.01 mmol); 2-iodobromobenzene (1.0 mmol); 2-aminoethanol (0.5 mmol); Et_3_N (2.0 mmol); p_CO_ = 1 bar; DMF (10 mL); T = 50 °C. (**b**) Reaction conditions: Pd(OAc)_2_ (0.01 mmol); Xantphos (0.01 mmol); 2-iodobromobenzene (1.0 mmol); 2-(methylamino)ethanol (0.5 mmol); Et_3_N (2.0 mmol); p_CO_ = 1 bar; DMF (10 mL); T = 100 °C.

To gain a deeper insight into the formation of the amide and ester functionality, the time plot of the carbonylation reaction was carried out in the presence of 2-aminoethanol (**2a**) and 2-(methylamino)ethanol (**2b**) (Figure 1a,b). Using 2-aminoethanol (**2a**), the aminocarbonylation reaction starts, and amide alcohol (**3a**) was formed selectively with 18% conversion. In the second hour, interestingly, no increase in **3a** was observed, but the slow formation of the amide-ester (**6a**) appeared. In the following, the increase in **6a** continued, while the amide alcohol content remained stable. Similar observations can be made in the carbonylation reaction of 2-(methylamino)ethanol (**2b**) at 100 °C, but the amide alcohol (**3b**) content stabilized at a higher level. The time plots clearly indicate that under the selected conditions (optimized to amide-ester formation), the alkoxycarbonylation reaction begins much earlier than before all the *N*-nucleophiles are depleted, completing the aminocarbonylation step. In the presence of 1,2-dibromobenzene (**1b**), the alkoxycarbonylation reaction occurred only to a small extent; thus, the iodoaromatic part proved to be essential for the effective synthesis of amide-ester derivatives.

As a continuation, the carbonylation reaction of 1,2-diiodobenzene (**1c**) was also studied in the presence of heterobifunctional *N*,*O*-nucleophiles. Using the initial conditions applied with the other substrates (**1a** and **1b**) and 2-aminoethanol (**2a**), complete conversion and selective imide (**4a**) formation was observed according to the results reported in the literature [22,23,24,25]. After the first aminocarbonylation step, the reactive *ortho*-positioned iodoaromatic moiety highly favored the intramolecular aminocarbonylation with the nearby located secondary amide functionality, forming succinimide derivatives. After many unsuccessful attempts to modify the reaction outcome, our attention was turned to other *N*,*O*-nucleophiles bearing secondary amine functional groups, which excludes the formation of imides. Using 2-(methylamino)ethanol (**2b**), 2-iodoamide alcohol (**3c**) can be produced with acceptable selectivity (Table 4, entry 1), but alkoxycarbonylation cannot be realized. Phthalic anhydride (**9a**), anthraquinone, and traces of other unidentified products were also observed in the reaction mixture. Using DABCO or Na_3_PO_4_ as the base instead of triethyl amine, benzoxazocine dione derivative (**8b**) was also identified with 16 and 33% selectivity, respectively (entries 2 and 3). In order to increase selectivity for the eight-membered ring heterocycle, a thorough investigation was conducted that included ligand, base, and solvent effects (see Supplementary Materials). Xantphos as a useful ligand of carbonylation reactions seemed to be sufficient in this reaction as well as using K_2_CO_3_ base, but the chemoselectivity remained moderate (38%, entry 6). Unfortunately, a large amount of phthalic anhydride was formed under various conditions. Attempts to remove the anhydride were made by applying other solvents, bases, and ligands, as well as modifying the temperature (entries 9 and 10) and the pressure of carbon monoxide and increasing the nucleophile ratio. No improvement could be achieved. Finally, a slight increase in chemoselectivity was seen using the anhydrous conditions (2-(methylamino)ethanol and DMF was dried over molecular sieves) (48%, entry 11). Interestingly, in the presence of other amino alcohols, the phthalic anhydride content was significantly decreased, resulting in much better chemoselectivity.

**Figure 2:**
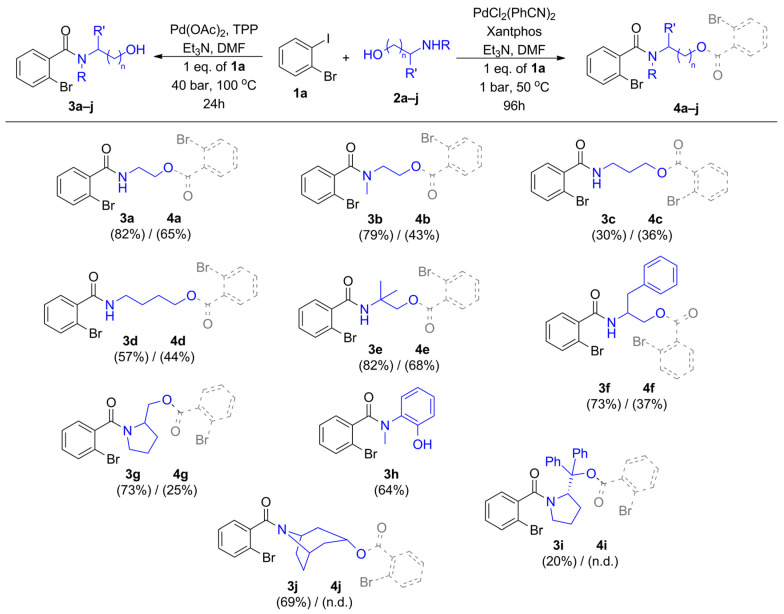
Nucleophile scope. The synthesized amide alcohols (**3a**–**j**, structures with OH) and amide-esters (**4a**–**j**, structures with dotted bonds).

Based on the detailed optimization reactions, we selected the best conditions for the synthesis of amide alcohols (Table 1, entry 5 as condition ‘A’) and amide-esters (Table 2, entry 6 as condition ‘B’), transforming various amino alcohols (**2a**–**2j**) and 2-iodobromobenzene (**1a**) (Figure 2). The isolated yields of the target compounds were varied between a wide range, and amide alcohols (**3a**–**3j**) were produced in higher yields compared to amide-esters (**4a**–**4j**), but normal-chain amino alcohols with elongated methylene bridges gave slightly lower isolated yields (**3c** and **3d**). Branched-chain nucleophiles like 2-methyl-2-aminopropanol (**2e**) and phenylalaninol (**2f**) took place in amino- and alkoxycarbonylation reactions with generally good selectivity and yields (**3e**, **4e**, **3f**, **4f**). Various nucleophiles bearing secondary amine groups were also transformed successfully to the corresponding mono- and dicarbonylated derivatives (**3b**, **4b**, **3g**–**3j,** and **4g**). Nortropine (**2j**) and diphenyl substituted pyrrolidine methanol (**2i**) as secondary and tertiary alcohols provided the amide-ester derivatives (**4i** and **4j**) only in traces; these compounds cannot be isolated in sufficient amounts. A large-scale experiment was also performed for amide alcohol production under optimized conditions (Table 1, entry 5). In the reaction of 2-bromoiodobenzene (**1a**) (10 mmol, 2.82 g) and 2-(methylamino)ethanol (**2b**) (10 mmol, 0.75 g), the targeted 2-bromo-N-(2-hydroxyethyl)-N-methylbenzamide (**3b**) was formed with 55.1% yield (1.4151 g).

**Figure 3:**
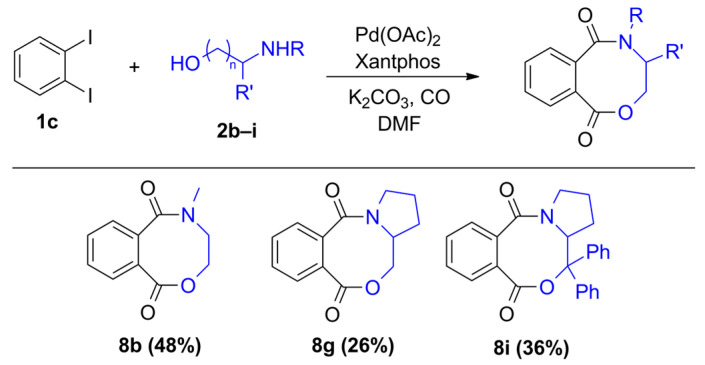
Products of intramolecular amino-alkoxy carbonylation reactions.

Similarly, we attempted the synthesis of benzoxazocine derivatives using 1,2-diiodobenzene (**1c**) and some secondary amino alcohols (Figure 3). The targeted compounds were available with moderate yields using 2-(methylamino)ethanol (**2b**) and pyrrolidine methanol (**2g**) under the optimized conditions (Table 4, entry 11). The diphenyl derivative of pyrrolidine methanol (**2i**) gave similar results in forming an eight-membered ring heterocycle, but nortropine (**2j**) was not a capable nucleophile of the desired reaction. Based on the GC-MS measurements, the oxazocine ring can be formed with aromatic *N*-methyl aminophenol (**2h**), but the compound decomposed during chromatographic isolation.

**Figure 4:**
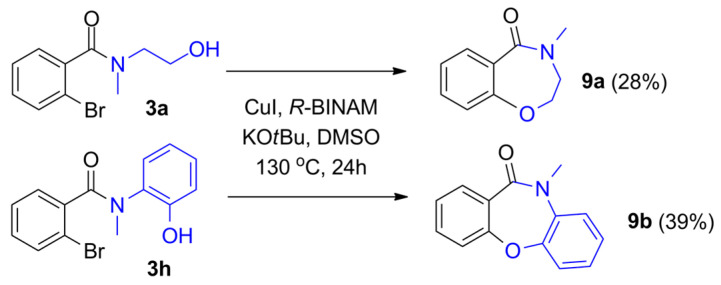
Intramolecular C-O coupling of 2-bromo amide alcohols forming benzoxazepine derivatives.

Finally, we attempted the copper-catalyzed intramolecular C-O coupling of the initially synthesized 2-bromo amide alcohols. Based on the literature focusing on intramolecular Ullmann coupling [29,30,31], various conditions were tested (optimization reactions are included in the Supplementary Materials). In the presence of *R*-BINAM (1,1′-Binaphthyl-2,2′-diamine) as a ligand and KO*t*Bu as a base, in DMSO, the benzoxazepine and dibenzoxazepine derivatives were formed with moderate isolated yields using 2-(methylamino)ethanol and 2-(methylamino)phenol as heterobifunctional nucleophiles, respectively (Figure 4).

## 3. Materials and Methods

All chemicals, including palladium precursors, ligands, solvents, and additives were purchased from Sigma-Aldrich Kft Budapest, Hungary, and were used without further purification. Equipment and glassware were purchased from Sigma-Aldrich Kft, Budapest, Hungary. High pressure autoclaves (Parr pressure vessels) were purchased from LAB-EX Kft, Budapest, Hungary.

In a typical atmospheric carbonylation reaction, a three-necked flask was assembled with a stopper, valve, and a condenser with a balloon. A magnetic stir bar was placed in the flask and the joints were greased. After changing the atmosphere of the system to argon gas (5.0), the stopper was removed and the reactants were transferred to the flask during continuous argon gas flow. First, the precursor and the ligands were washed using the solvent, followed by the substrate, nucleophile, and the base. After adding all the reactants, the atmosphere was changed to carbon monoxide and the mixture was heated to the appropriate temperature with a heat-on block and stirred during the reaction. When the reaction time was over, the system was cooled, the carbon monoxide was pumped out, and the mixture was filtered. The crude solution was immediately analyzed by gas chromatography (a specific atmospheric carbonylation reaction is described in the Supplementary Materials files).

In a typical high-pressure carbonylation reaction, a Schlenk flask was used to prepare the reaction mixture. The atmosphere of the flask was changed to argon gas (5.0) three times and the reactants were transferred during continuous argon gas flow. First, the precursor and the ligands were washed using the solvent, followed by the substrate, nucleophile, and the base (using solid bases (K_2_CO_3_, Na_2_CO_3_, Na_3_PO_4_, KO*t*Bu, etc.), the base was transferred separately to the autoclave, not in the Schlenk flask). The mixture was stirred with a magnetic stirrer and the homogeneous solution was removed with a syringe during continuous argon gas flow. The atmosphere of the autoclave was also changed to argon gas three times and then the reaction mixture was transferred from the syringe. The autoclave was closed thoroughly and pressurized by carbon monoxide. The mixture was heated in an oil bath for the given time. When the reaction time was over, the system was cooled, the carbon monoxide was released, and the mixture was filtered. The crude solution was immediately analyzed by gas chromatography (a specific high-pressure carbonylation reaction is described in the Supplementary Materials file).

Working with carbon monoxide requires particular attention. Well-ventilated hood and gas sensors are obligatory!

All other experimental data, including reaction condition screening, product isolation, characterization, the copy of the spectra and chromatography conditions, and specifications, are given in the Supplementary Materials file.

## 4. Conclusions

The carbonylation reactions of iodo- and bromo-substituted *ortho*-phenylene dihalides were investigated in the presence of aminoethanols as heterobifunctional *N*,*O*-nucleophiles. This study demonstrates the high synthetic potential of 1,2-dihalides beyond imide production. Under optimized conditions, the selective aminocarbonylation of iodo-aryl moiety proved to be feasible, excluding a wide range of side products. Additionally, the much more challenging alkoxycarbonylation was also realized, allowing for the synthesis of amide-esters beside amide alcohols. Detailed reaction screening was performed with 1,2-dibromobenzene as well, but the activation of the aryl-bromide functionality was more complicated, and harsher reaction conditions favored the formation of phthalimides. Furthermore, intramolecular alkoxycarbonylation using 1,2-diiodobenzene and C-O coupling reactions of 2-bromo amide alcohols were also realized using amino alcohols bearing secondary amine functionality. These novel methodologies allow for the synthesis of seven- and eight-membered ring benzoxazepine and benzoxazocine derivatives. These structural moieties are present in various important pharmaceuticals and are of particular interest for future drug design.

## Figures and Tables

**Table 1:**
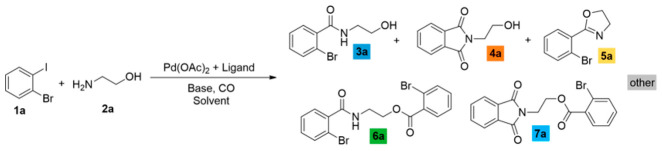
Carbonylation reactions of 2-bromoiodobenzene with 2-aminoethanol.

#	Ligand [mmol]	1a [mmol]	2a [mmol]	Base	p_CO_ [bar]	Solvent	T [°C]	t [h]	Conversion and Composition of the Reaction Mixture
1	TPP [0.02]	0.5	0.5	Et_3_N	1	DMF	100	48	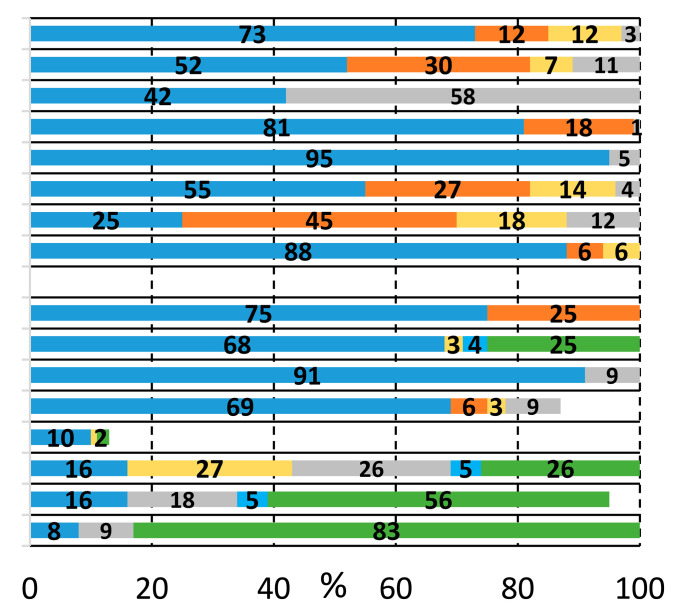
2	TPP [0.02]	0.5	0.5	Cs_2_CO_3_	1	DMF	100	24	
3	TPP [0.02]	0.5	0.5	KO*t*Bu	1	DMF	100	24	
4	TPP [0.02]	0.5	1.0	Et_3_N	1	DMF	100	24	
5	TPP [0.02]	0.5	0.5	Et_3_N	40	DMF	100	24	
6	Xantphos [0.01]	0.5	0.5	Et_3_N	1	DMF	100	48	
7	*t*Bu-Xantphos [0.01]	0.5	0.5	Et_3_N	1	DMF	100	24	
8	Xantphos [0.01]	0.5	0.5	Et_3_N	1	MeCN	82	24	
9	Xantphos [0.01]	0.5	0.5	Et_3_N	1	NMP	100	96	
10	Xantphos [0.01]	0.5	0.5	Et_3_N	1	DMF	75	48	
11	Xantphos [0.01]	0.5	0.5	Et_3_N	1	DMF	50	48	
12	Xantphos [0.01]	0.5	0.5	Et_3_N	40	DMF	75	48	
13	-	0.5	0.5	Et_3_N	1	DMF	75	48	
14	TPP [0.02]	1.0	0.5	Et_3_N	1	DMF	50	24	
15	Xantphos [0.01]	1.0	0.5	Et_3_N	1	DMF	100	24	
16	Xantphos [0.01]	1.0	0.5	Et_3_N	1	DMF	75	24	
17	Xantphos [0.01]	1.0	0.5	Et_3_N	1	DMF	50	72	
Reaction conditions: Pd(OAc)_2_ (0.01 mmol); Base (2.0 mmol); Solvent (10 mL). Conversion and composition are determined by GC.									

**Table 2:**
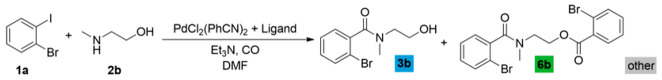
Carbonylation reactions of 2-bromoiodobenzene with 2-(methylamino)ethanol.

#	Ligand [mmol]	1a [mmol]	p_CO_ [bar]	T [°C]	Conversion and Composition of the Reaction Mixture
1	TPP [0.02]	0.5	1 bar	100	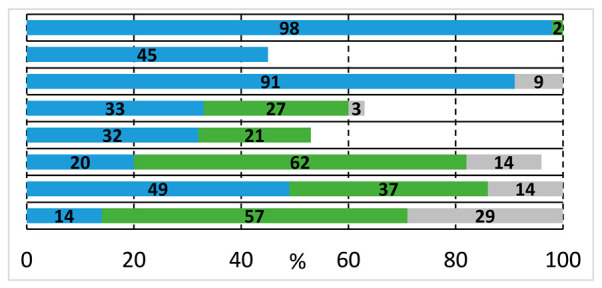
2	TPP [0.02]	1.0	1 bar	100	
3	Xantphos [0.01]	0.5	1 bar	100	
4	Xantphos [0.01]	1.0	1 bar	100	
5	Xantphos [0.01]	1.0	1 bar	75	
6 ^a^	Xantphos [0.01]	1.0	1 bar	50	
7	Xantphos [0.01]	0.5	40 bar	100	
8	Xantphos [0.01]	1.0	40 bar	100	
Reaction conditions: PdCl_2_(PhCN)_2_ (0.01 mmol); **2b** (0.5 mmol); Et_3_N (2.0 mmol); DMF (10 mL); t = 24 h. Conversion and composition determined by GC. ^a^ Reaction time: 96 h.					

**Table 3:**
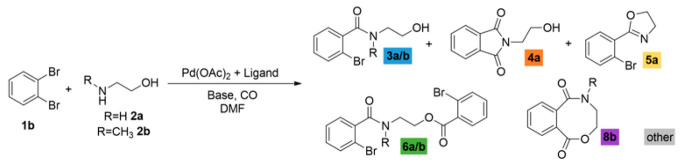
Carbonylation reactions of 1,2-dibromobenzene with 2-aminoethanol and 2-(methylamino)ethanol.

#	Ligand [mmol]	1b [mmol]	2a [mmol]	Base	p_CO_ [bar]	T [°C]	Conversion and Composition of the Reaction Mixture
1	TPP [0.02]	0.5	0.5	Et_3_N	1	100	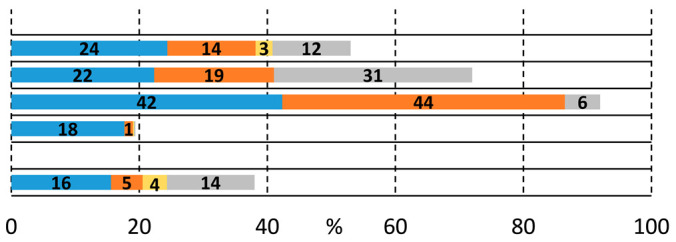
2	Xantphos [0.01]	0.5	0.5	Et_3_N	1	100	
3	Xantphos [0.02]	0.5	0.5	Et_3_N	1	100	
4	Xantphos [0.01]	0.5	1.0	Et_3_N	1	100	
5	Xantphos [0.01]	0.5	0.5	Et_3_N	1	75	
6	Xantphos [0.01]	0.5	0.5	Et_3_N	1	50	
7	Xantphos [0.01]	1.0	0.5	Et_3_N	1	100	
		**1b** [mmol]	**2b** [mmol]				
8	Xantphos [0.01]	0.5	0.5	Et_3_N	1	100	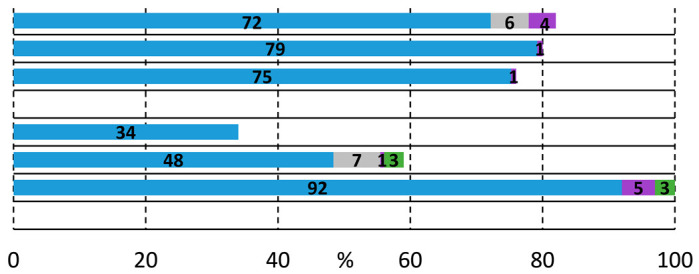
9	Xantphos [0.01]	0.5	0.5	Et_3_N	1	130	
10 ^a^	Xantphos [0.01]	0.5	0.5	Et_3_N	1	100	
11	Xantphos [0.01]	0.5	0.5	Et_3_N	20	100	
12	N-Xantphos [0.01]	1.0	0.5	Et_3_N	1	100	
13	Xantphos [0.01]	1.0	0.5	Et_3_N	1	120	
14	Xantphos [0.01]	1.0	0.5	Cs_2_CO_3_	1	120	
15	Xantphos [0.01]	1.0	0.5	K*t*Bu	1	120	
Reaction conditions: Pd(OAc)_2_ (0.01 mmol); Base (2.0 mmol); DMF (10 mL); t = 24 h. Values determined by GC. ^a^ PdCl_2_(PhCN)_2_ was used.							

**Table 4:**
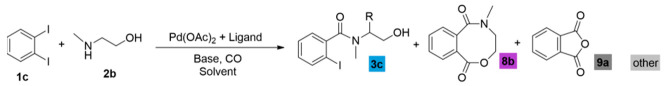
Carbonylation reactions of 1,2-diiodobenzene with 2-(methylamino)ethanol.

#	Ligand [mmol]	Base	Solvent	T [°C]	Conversion and Composition of the Reaction Mixture
1	TPP [0.005]	Et_3_N	DMF	75	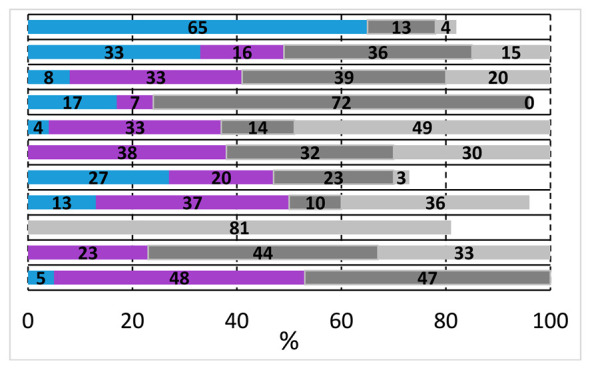
2	TPP [0.005]	DABCO	DMF	75	
3	TPP [0.005]	Na_3_PO_4_	DMF	75	
4	Xantphos [0.005]	Et_3_N	DMF	75	
5	Xantphos [0.005]	Na_3_PO_4_	DMF	75	
6	Xantphos [0.005]	K_2_CO_3_	DMF	75	
7	TPP [0.005]	K_2_CO_3_	MeCN	75	
8	TPP [0.005]	K_2_CO_3_	Dioxane	75	
9	Xantphos [0.005]	K_2_CO_3_	DMF	50	
10	Xantphos [0.005]	K_2_CO_3_	DMF	100	
11	Xantphos [0.005]	K_2_CO_3_	DMF (dry)	75	
Reaction conditions: Pd(OAc)_2_ (0.0025 mmol); Base (0.5 mmol); 1,2-Diiodobenzene (0.125 mmol); 2-(Methylamino)ethanol (0.125 mmol) Solvent (2.5 mL); p_CO_ = 1 bar; t = 24 h. Values determined by GC.					

## Data Availability

The original contributions presented in this study are included in the article/Supplementary Materials; further inquiries can be directed to the corresponding authors.

## Supplementary Materials

The following supporting information can be downloaded at: https://www.mdpi.com/article/10.3390/molecules29235620/s1. Tables S1–S4. Tables of optimization reactions; Spectroscopic characterization of products (Pages 7–15); Copies of MS, ^1^H and ^13^C NMR spectra (Pages 16–40).
