# Supplementary material for: Palladium-Catalyzed Selective Carbonylation Reactions of Ortho-Phenylene Dihalides with Bifunctional N,O-Nucleophiles
Source: Molecules. 2024 Nov 27;29(23):5620. doi: 10.3390/molecules29235620 (PMC11643691; doi:10.3390/molecules29235620)
Supplement: Supplementary file 1 [file molecules-29-05620-s001.zip › molecules-3305913-supplementary.pdf]

Supporting Information

Palladium-Catalyzed Selective Carbonylation Reactions of *Ortho*-Phenylene Dihalides with Bifunctional *N,O*-Nucleophiles

Fanni Bede [1], Attila Takács [2,3], László Kollár [1,2,3] and Péter Pongrácz [1,*]

[1] Department of General and Inorganic Chemistry, University of Pécs, Ifjúság u. 6., H-7624 Pécs, Hungary; fanni.bede@gmail.com (F.B.); kollar@gamma.ttk.pte.hu (L.K.)
[2] HUN-REN-PTE Research Group for Selective Chemical Syntheses, Ifjúság u. 6., H-7624 Pécs, Hungary; takacsattila@gamma.ttk.pte.hu
[3] János Szentágothai Research Centre, University of Pécs, Ifjúság u. 20., H-7624 Pécs, Hungary
* Correspondence: pongracz@gamma.ttk.pte.hu

Page	Content
2	General considerations
2	General atmospheric carbonylation reaction
2	General high-pressure carbonylation reaction
2	Copper-catalyzed C-O coupling reaction
3	Reaction condition screening
3	Table S1. Carbonylation of 1,2-dibromobenzene in the presence of 2-aminoethanol
4	Table S2. Carbonylation of 1,2-dibromobenzene in the presence of 2-(methylamino)ethanol
5	Table S3. Carbonylation of 1,2-diiodobenzene in the presence of 2-(methylamino)ethanol
6	Table S4. Intramolecular C-O coupling of 2-bromo-N-(2-hydroxyethyl)-N-methylbenzamide (**3b**)
6	The general procedure of product isolation/purification
7	Characterization of the prepared compounds
16	Spectroscopic data (^{1}H, ^{13}C, MS) of prepared compounds

General considerations

Palladium precursors, phosphine ligands, solvents and other additives were purchased from Sigma-Aldrich Kft., Budapest, Hungary and were used without further purification. The reactions were performed under inert argon atmosphere using Schlenck techniques.

Conversion and selectivity were determined Shimadzu GC- gas chromatograph, using splitless injection and FID detector (column: DB-1MS or DB-5MS (30m x 0.250mm x 0.25µm) inlet temperature: 250 ºC; starting oven temperature: 50 ºC; rate: 15 ºC/min; final temperature: 320 ºC, carrier gas: N_2 1.30 mL/min).

For MS measurements Shimadzu GC-MS was used with electron spray ionisation (ESI). The data are given as mass unit per charge (m/z) and intensities are given in brackets.

The ^{1}H- and ^{13}C-NMR spectra were recorded on a Bruker Avance-III 500 spectrometer at 500 and 125 MHz, respectively. Chemical shifts δ (ppm) are given relative to solvent: references for $CDCl_3$ were 7.26 ppm (^{1}H-NMR) and 77.16 ppm (^{13}C-NMR). Multiplets were assigned as s (singlet), d (doublet), t (triplet), q (quartet), dd (doublet of doublet), m (multiplet).

High-resolution mass spectrometry (HRMS) data were obtained using a 6530 Accurate-Mass Quadrupole Time-of-Flight (Q-TOF) LC/MS system (Agilent Technologies, Singapore). The compounds were ionized by an Agilent Jet Stream electrospray ion source.

General atmospheric carbonylation reaction

In a typical experiment, catalyst precursor [Pd(OAc)$_2$] (2 mol %) and ligand (4 mol % monophosphine, 2 mol % diphosphine) were placed in a three-necked flask, which was refilled with argon gas three times. DMF (10 mL), 2-iodobromobenzene (0.5 mmol), 2-aminoethanol (0.5 mmol) and Et_3N (2.0 mmol) were transferred to the flask, against constant argon flow. Finally, the atmosphere was changed to carbon monoxide (1 bar). The mixture was placed in a heating block (100 ^oC) and stirred with a magnetic stirrer for 24 hours. After the given reaction time the reaction mixture was cooled, filtered and immediately analyzed by GC and GC–MS.

General high-pressure carbonylation reaction

In a typical experiment, catalyst precursor [Pd(OAc)$_2$] (2 mol %) and ligand (4 mol % monophosphine, 2 mol % diphosphine) were placed in a stainless steel autoclave and the atmosphere was refilled with argon gas three times. DMF (10 mL), iodobenzene (0.5 mmol), 2-aminoethanol (0.5 mmol) and Et_3N (2.0 mmol) were transferred to the autoclave. The reaction vessel was pressurized to 40 bar carbon monoxide, placed in a heating block (100 ^oC) and stirred with a magnetic stirrer for 24 hours. After cooling and venting the autoclave, the pale yellow solution was removed and immediately analyzed by GC and GC-MS.

Copper-catalyzed C-O coupling reaction

In a typical experiment, catalyst precursor [CuI] (20 mol %) and ligand [R-BINAM] (20 mol %) were placed in a three-necked flask, which was refilled with argon gas three times. DMSO (10 mL), substrate (0.5 mmol) and KOtBu (1.0 mmol) were transferred to the flask, against constant argon flow. The mixture was placed in a heating block (130 ^oC) and stirred with a magnetic stirrer for 24 hours under argon atmosphere (1 bar). After the given reaction time the reaction mixture was cooled, filtered and immediately analyzed by GC and GC–MS.

Reaction condition screening

Table S1. Carbonylation of 1,2-dibromobenzene in the presence of 2-aminoethanol (reaction conditions under the table)

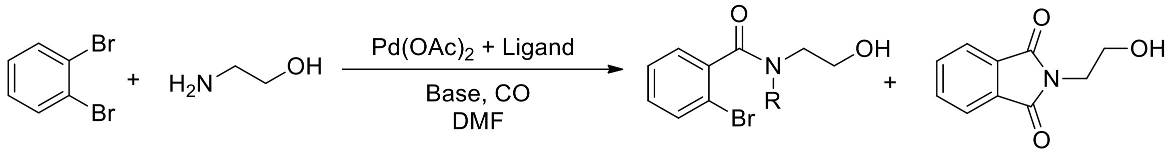

#	Ligand [0.002 mmol]		Substrate-Nucleophile ratio	Conv. [%]	Products [%]		
					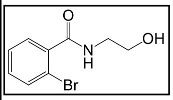	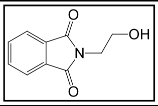	other
1	Xantphos	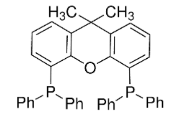	1:1	34	25	7	2
2	*4,5-Bis(diphenylphosphino)-9,9-dimethylxanthene*		1:2	37	28	7	2
3	N-XantPhos	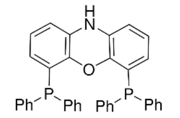	1:1	49	29	16	4
4	*NiXantphos, 4,6-Bis(diphenylphosphino)-10H-phenoxazine, 4,6-Bis(diphenylphosphino)phenoxazine*		1:2	55	40	14	1
5	tBu-XantPhos	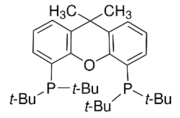	1:1	0	-	-	-
6	*9,9-Dimethyl-4,5-bis(di-tert-butylphosphino)xanthene*		1:2	0	-	-	-
7	DPPF	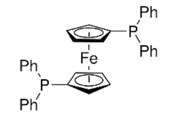	1:1	0	-	-	-
8	*1,1'-Ferrocenediyl-bis(diphenylphosphine)*		1:2	0	-	-	-
9	DiPrPF	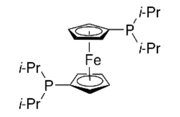	1:1	18	11	6	1
10	1,1′-Bis(diisopropylphosphino)ferrocene		1:2	23	15	7	1
11	DPEPhos	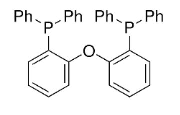	1:1	8	6	1	1
12	*(Oxydi-2,1-phenylene)bis(diphenylphosphine)*		1:2	9	7	1	1
Reaction conditions: $Pd(OAc)_2$ (0.002 mmol); 1,2-dibromobenzene (0.1 mmol); 2-aminoethanol (1 or 2 eq.); Et_3N (0.4 mmol); DMF (2 mL); p_{CO}=1 bar; T=100°C; t=24 h.							

Table S2. Carbonylation of 1,2-dibromobenzene in the presence of 2-(methylamino)ethanol (reaction conditions under the table)

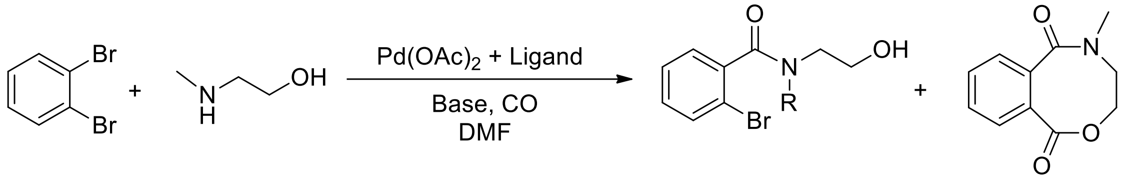

#	Ligand [0.002 mmol]		Conv. [%]	Products [%] 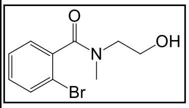	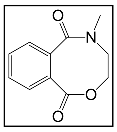
1	Xantphos<br>*4,5-Bis(diphenylphosphino)-9,9-dimethylxanthene*	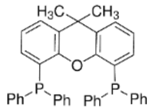	30	30	trace
2	N-XantPhos<br>*NiXantphos, 4,6-Bis(diphenylphosphino)-10H-phenoxazine, 4,6-Bis(diphenylphosphino)phenoxazine*	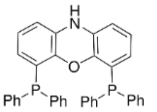	42	41	1
3	DPPE<br>*1,2-Bis(diphenylphosphino)ethane*	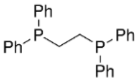	2	2	-
4	DPPB<br>*1,4-Bis(diphenylphosphino)butane*	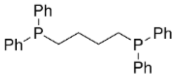	3	3	-
5	DPPH<br>*1,6-Bis(diphenylphosphino)hexane*	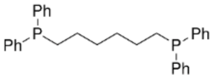	<1	<1	-
6	DCyPB<br>*1,4-Bis(dicyclohexylphosphino)butane*	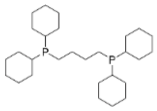	6	6	-
7	d*t* BuPB<br>*1,2-Bis(di-tert-butylphosphinomethyl)benzene*	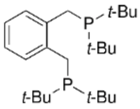	<1	<1	-
8	DPEPhos<br>*(Oxydi-2,1-phenylene)bis(diphenylphosphine)*	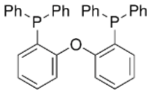	36	36	trace
9	BPPBenz<br>*1,2-Bis(diphenylphosphino)benzene*	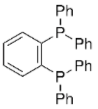	<1	<1	-
10	DPPF<br>*1,1'-Ferrocenediyl-bis(diphenylphosphine)*	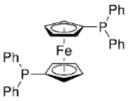	18	18	trace
11	3,5-CF_3-PPh_3<br>*Tris[3,5-bis(trifluoromethyl)phenyl]phosphine*	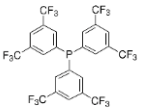	<1	<1	-
12	$DCy_2(iPr)_2Ph$<br>*Dicyclohexyl-(2,6-diisopropylphenyl)phosphine*	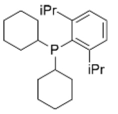	<1	<1	-

Reaction conditions: $Pd(OAc)_2$ (0.002 mmol); 1,2-dibromobenzene (0.1 mmol); N-methylethanolamine (0.1 mmol); Et_3N (0.4 mmol); DMF (2 mL); p_{CO}=1 bar; T=100°C; t=24 h.

Table S3. Carbonylation of 1,2-diiodobenzene in the presence of 2-(methylamino)ethanol

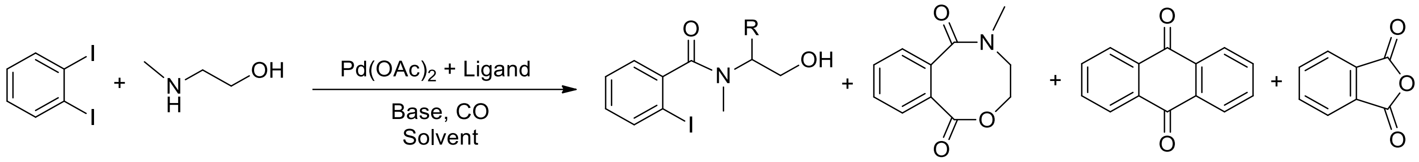

#	Ligand [mmol]	Base [mmol]	Solvent	T [°C]	t [h]	Conv. [%]	Selectivity [%]				
							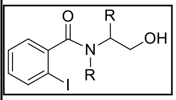	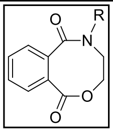	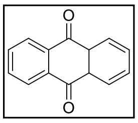	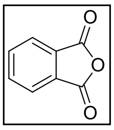	Other[a]
1	TPP [0.005]	Et_3N [0.5]	DMF	75	24	84	74	0	6	20	0
2	TPP [0.005]	DABCO [0.5]	DMF	75	24	100	33	16	15	36	0
3	TPP [0.005]	DBU [0.5]	DMF	75	24	0	0	0	0	0	0
4	TPP [0.005]	Na_3PO_4 [0.5]	DMF	75	24	100	8	33	20	39	0
5	Xantphos [0.005]	Et_3N [0.5]	DMF	75	24	95	17	7	0	72	0
6	tBu-Xantphos [0.005]	Et_3N [0.5]	DMF	75	24	7	100	0	0	0	0
7	DPPF [0.005]	Et_3N [0.5]	DMF	75	24	16	100	0	0	0	0
8	$DCy_2(iPr)_2Ph$ [0.005]	Et_3N [0.5]	DMF	75	24	100	11	4	26	39	20
9	dtBuPB [0.005]	Et_3N [0.5]	DMF	75	24	24	100	0	0	0	0
10	$3,5\text{-}CF_3\text{-}PPh_3$ [0.005]	Et_3N [0.5]	DMF	75	24	69	64	9	0	16	11
11	TPP [0.005]	Et_3N [0.5]	DMSO	75	24	71	0	0	0	0	100
12	TPP [0.005]	Na_3PO_4 [0.5]	DMSO	75	24	100	21	14	24	32	9
13	TPP [0.005]	K_2CO_3 [0.5]	DMSO	75	24	100	0	0	0	10	90
14	TPP [0.005]	Et_3N [0.5]	MeCN	75	24	25	94	0	0	0	6
15	TPP [0.005]	Na_3PO_4 [0.5]	MeCN	75	24	40	78	13	0	0	9
16	TPP [0.005]	K_2CO_3 [0.5]	MeCN	75	24	73	27	25	4	21	24
17	TPP [0.005]	Et_3N [0.5]	Dioxane	75	24	12	50	33	0	0	17
18	TPP [0.005]	Na_3PO_4 [0.5]	Dioxane	75	24	40	53	5	0	0	42
19	TPP [0.005]	K_2CO_3 [0.5]	Dioxane	75	24	60	13	37	10	36	10
20	TPP [0.005]	Et_3N [0.5]	THF	75	24	7	0	0	0	0	100
21	TPP [0.005]	Na_3PO_4 [0.5]	THF	75	24	7	0	0	0	0	100
22	TPP [0.005]	K_2CO_3 [0.5]	THF	75	24	7	0	0	0	0	100
23	Xantphos [0.005]	K_2CO_3 [0.5]	DMF	50	24	81	0	0	0	0	100
24	Xantphos [0.005]	K_2CO_3 [0.5]	DMF	75	24	100	0	38	32	30	0
25	Xantphos [0.005]	K_2CO_3 [0.5]	DMF	100	24	100	0	23	44	33	4
26	Xantphos [0.005]	Na_3PO_4 [0.5]	DMF	50	24	100	8	16	13	42	21
27	Xantphos [0.005]	Na_3PO_4 [0.5]	DMF	75	24	100	5	32	14	49	0
28	Xantphos [0.005]	Na_3PO_4 [0.5]	DMF	100	24	100	5	33	15	47	0
29	Xantphos [0.01]	K_2CO_3 [0.5]	DMF (anhydr.)	75	24	100	5	48	47	0	12
30[a]	Xantphos [0.01]	K_2CO_3 [0.5]	DMF (anhydr.)	75	24	100	0	38	0	31	31
30[b]	Xantphos [0.01]	K_2CO_3 [0.5]	DMF (anhydr.)	75	24	100	0	38	0	31	31

Reaction conditions: $Pd(OAc)_2$ (0.0025 mmol), 1,2-diiodobenzene (0.125 mmol), N-methylethanolamine (0.125 mmol), Solvent (5 mL), pCO=1 bar. [a] Unidentified side products. [a] N-methylethanolamine (0.25 mmol). [b] N-methylethanolamine (0.50 mmol).

Table S4. Intramolecular C-O coupling of 2-bromo-N-(2-hydroxyethyl)-N-methylbenzamide (**3b**)

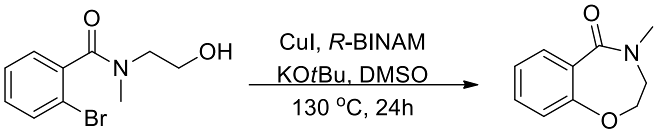

#	precursor [mmol]	ligand [mmol]	substrate (**3b**) [mmol]	additive [mmol]	base [mmol]	solvent	T [°C]	t [h]	conversion [%]	oxazepine selectivity [%]
1	CuI [0.05]	R-BINAM [0.05]	0.25	-	Cs_2CO_3 [0.5]	DMF [5 ml]	130	24	100	0
2	CuI [0.05]	R-BINAM [0.05]	0.25	-	Cs_2CO_3 [0.5]	dioxane [5 ml]	110	24	100	0
3	$Pd(OAc)_2$ [0.025]	-	0.25	Bu_4NI [0.375]	Cs_2CO_3 [0.375]	DMF [4 ml]	110	24	100	0
4	CuI [0.05]	R-BINAM [0.05]	0.25	-	Cs_2CO_3 [0.5]	DMF [5 ml]	110	24	100	0
5	$Pd(OAc)_2$ [0.025]	-	0.25	Bu_4NI [0.375]	Cs_2CO_3 [0.375]	DMF [6 ml]	110	24	100	0
6	CuI [0.05]	R-BINAM [0.05]	0.25	-	Cs_2CO_3 [0.5]	dioxane [5 ml]	100	24	100	0
7	$Pd(OAc)_2$ [0.025]	TPP [0.0375]	0.25	Bu_4NI [0.375]	Cs_2CO_3 [0.375]	DMF [4 ml]	130	24	100	0
8	$PdCl_2$ [0.025]	TPP[0.0375]	0.25	Bu_4NI [0.375]	Cs_2CO_3 [0.375]	DMF [4 ml]	130	24	100	0
9	CuI [0.025]	R-BINAM [0.025]	0.125	-	Cs_2CO_3 [0.25]	DMF [2.5 ml]	130	24	100	0
10	CuI [0.025]	R-BINAM [0.025]	0.125	-	Cs_2CO_3 [0.25]	DMSO [2.5 ml]	130	24	100	0
11	CuI [0.025]	R-BINAM [0.025]	0.125	-	Na_2CO_3 [0.25]	DMF [2.5 ml]	130	24	100	0
12	CuI [0.025]	R-BINAM [0.025]	0.125	-	Na_2CO_3 [0.25]	DMSO [2.5 ml]	130	24	100	0
13	CuI [0.025]	R-BINAM [0.025]	0.125	-	KOtBu [0.25]	DMF [2.5 ml]	130	24	100	0
14	CuI [0.025]	R-BINAM [0.025]	0.125	-	KOtBu [0.25]	DMSO [2.5 ml]	130	24	100	24
15	CuI [0.025]	R-BINAM [0.025]	0.125	-	DABCO [0.25]	DMF [2.5 ml]	130	24	100	0
16	CuI [0.025]	R-BINAM [0.025]	0.125	-	DABCO [0.25]	DMSO [2.5 ml]	130	24	100	0
17	CuI [0.025]	R-BINAM [0.025]	0.125	-	Et_3N [0.25]	DMF [2.5 ml]	130	24	100	0
18	CuI [0.025]	R-BINAM [0.025]	0.125	-	Et_3N [0.25]	DMSO [2.5 ml]	130	24	100	0
19	CuI [0.025]	R-BINAM [0.025]	0.125	-	DBU [0.25]	DMF [2.5 ml]	130	24	100	0
20	CuI [0.025]	R-BINAM [0.025]	0.125	-	DBU [0.25]	DMSO [2.5 ml]	130	24	100	0
21	CuI [0.1]	R-BINAM [0.1]	0.5	-	KOtBu [1.0]	DMSO [10 ml]	130	24	100	35
22	CuI [0.1]	R-BINAM [0.1]	0.5	-	KOtBu [1.0]	DMSO [10 ml]	130	24	100	34

The general procedure of product isolation/purification

The solvent of the reaction mixture was evaporated under vacuum and the crude product was purified by column chromatography on silica gel (0.063-0.2) using different eluents (given in the characterization) to afford the corresponding products.

Characterization of the prepared compounds

**3a**	Isolated yield: 0.0996 g (82%). R_F: 0.46 (on silica gel chloroform:2-propanol=9:1). Yellow high viscosity liquid.
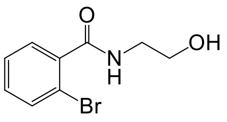	δ_H (500 MHz, $CDCl_3$) 7.62 (1H, d, 7.5 Hz, *Ar*), 7,58 (1H, d, 7.5 Hz, *Ar*), 7.39 (1H, t, 7.6 Hz, *Ar*), 7.3 (1H, t, 7.6 Hz, *Ar)*, 6.82 (1H, s, N*H*), 3.77 (2H, t, 4.9 Hz, OCH_2), 3.67 (2H, m, NHCH_2).
	δ_C (125.7 MHz, $CDCl_3$) 168.4, 133.4, 131.4, 129.7, 127.6, 119.3, 62.1, 42.8.
	MS m/z (rel int.): 245/243 (1, M^+), 225/227 (7), 200/202 (37), 183/185 (100), 155/157 (25), 146 (15), 77 (11).
	HRMS (ESI-Q-TOF) m/z calcd for $C_9H_{10}BrNO_2$ $[M+H]^+$: 243.9968; found: 243.9970.

**4a**	Isolated yield: 0.0382 g (40%). R_F: 0.40 (on silica gel chloroform:ethyl acetate=7:3). Brownish white crystals.
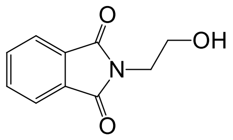	δ_H (500 MHz, $CDCl_3$) 7.88-7.86 (2H, m, *Ar*), 7.75-7.74 (2H, m *Ar*), 3.93-3.89 (4H, m, CH_2).
	δ_C (125.7 MHz, $CDCl_3$) 168.8, 134.1, 132.0, 123.4, 61.0, 40.9.
	MS m/z (rel int.): 191 (3, M^+), 160 (100), 148 (48), 133 (27), 104 (20), 77 (26), 50 (9).
	HRMS (ESI-Q-TOF) m/z calcd for $C_{10}H_9NO_3$ $[M+H]^+$: 192.0655; found: 192.0659.

6a	Isolated yield: 0.1381 g (65%). R_F: 0.49 (on silica gel chloroform:2-propanol=95:5). Yellowish brown high viscosity liquid.
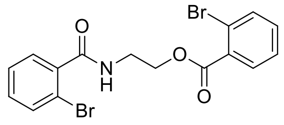	δ_H (500 MHz, $CDCl_3$) 7.86 (1H, d, 7.6 Hz, *Ar*), 7.69 (1H, d, 7.6 Hz, *Ar*), 7.61 (1H, d, 7.9 Hz, *Ar*), 7.58 (1H, d, 7.9 Hz, *Ar*), 7.43-7.36 (3H, m, *Ar*), 7.32 (1H, d, 7.9 Hz, *Ar*), 6.43 (1H, br s, N*H*), 4.61 (2H, t, 5.3 Hz, CH_2O), 3.95-3.92 (2H, m, CH_2NH).
	δ_C (125.7 MHz, $CDCl_3$) 167.7, 166.2, 137.5, 134.4, 133.4, 132.8, 131.9, 131.6, 131.4, 129.6, 127.6, 127.3, 121.6, 119.3, 64.3, 39.1.
	MS m/z (rel int.): 244/242 (14), 227/225 (31), 185/183 (100), 157/155 (16), 146 (33), 105 (9), 76 (19), 50 (9).

7a	Isolated yield: 0.0281 g (12%). R_F: 0.84 (on silica gel chloroform:ethyl acetate=9:1). Brownish white crystals.
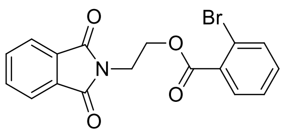	δ_H (500 MHz, $CDCl_3$) 7.91-7.89 (2H, m, *Ar*), 7.84 (1H, d, 7.9 Hz, *Ar*), 7.77-7.75 (2H, m, *Ar*), 7.65 (1H, d, 7.9 Hz, *Ar*), 7.40-7.37 (1H, m, *Ar*), 7.35-7.32 (1H, m, *Ar*), 4.62 (2H, t, 5.4Hz, CH_2O), 4.15 (2H, t, 5.4Hz, CH_2N).
	δ_C (125.7 MHz, $CDCl_3$) 168.1, 165.7, 134.3, 134.1, 132.7, 132.1, 131.7, 127.2, 123.5, 122.7, 121.9, 62.6, 37.0.

	MS m/z (rel int.): 185/183 (20), 173 (100), 160 (22), 105 (11), 77 (8).

3b	Isolated yield: 0.1015 g (79%). R_F: 0.56 (on silica gel chloroform:2-propanol=9:1). Yellowish brown high viscosity liquid.
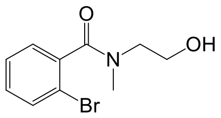	δ_H (500 MHz, $CDCl_3$) 7.60-7.56 (1H, m, *Ar*), 7.40-7.23 (3H, m, *Ar*), 3.93 (1H, br s), 3.78 (0.5H, br s), 3.70 (1H, br s), 3.61 (0.5H, br s), 3.33 (1H, br s), 3.17 (1H, s, C*H*$_3$), 2.93 (2H, s, C*H*$_3$)
	δ_C (125.7 MHz, $CDCl_3$) 170.6, 169.9, 138.2, 132.8, 132.7, 130.4, 130.2, 128.5, 127.9, 127.7, 127.6, 119.2, 119.0, 60.9, 59.7, 52.6, 50.5, 37.7, 33.1.
	MS m/z (rel int.): 259/257 (M^+, 2), 229/227 (15), 216/214 (15), 185/183 (100), 157/155 (19), 105 (8), 76 (18), 42 (11).
	HRMS (ESI-Q-TOF) m/z calcd for $C_{10}H_{12}BrNO_2$ $[M+H]^+$: 258.0124; found: 258.0124.

6b	Isolated yield: 0.0944 g (43%). R_F: 0.76 (on silica gel chloroform:2-propanol=95:5). Colorless high viscosity liquid.
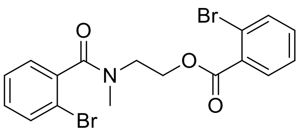	δ_H (500 MHz, $CDCl_3$) 7.86 (0.7H, d, 7.5 Hz, *Ar*), 7.72 (0.3H, d, 7.5Hz, *Ar*), 7.67-7.64 (1H, m, *Ar*), 7.56-7.53 (1H, m, *Ar*), 7.34-7.31 (3H, m, *Ar*), 7.25-7.20 (2H, m, *Ar*), 4.67 (1.4H, br s, C*H*$_2$), 4.46-4.41 (0.3H, m, C*H*$_2$), 4.39-4.34 (0.3H, m, C*H*$_2$), 4.05 (0.7H, br s, C*H*$_2$), 3.92 (0.7H, br s, C*H*$_2$), 3.67-3.61 (0.3H, m, C*H*$_2$), 3.51-3.47 (0.3H, m, C*H*$_2$), 3.24 (1H, s, C*H*$_3$), 2.96 (2H, s, C*H*$_3$).
	δ_C (125.7 MHz, $CDCl_3$) 169.6, 169.5, 165.9, 165.7, 138.3, 137.9, 134.5, 134.4, 133.0, 132.8, 131.8, 131.6, 131.3, 130.4, 130.3, 129.9 128.3, 127.8, 127.7, 127.6, 127.54, 127.50, 127.3, 127.2, 121.8, 121.7, 119.1, 119.0, 62.8, 62.4, 49.2, 46.3, 37.5, 33.0.
	MS m/z (rel int.): 258/256 (8), 241/293 (15), 228/226 (9), 185/183 (100), 160 (39), 132 (47), 105 (10), 76 (14).

3c	Isolated yield: 0.0386 g (30%). R_F: 0.26 (on silica gel chloroform:2-propanol=97:3). Reddish brown high viscosity liquid.
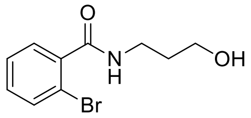	δ_H (500 MHz, $CDCl_3$) 7.61 (1H, d, 8.0 Hz, *Ar*), 7.53 (1H, d, 7.5 Hz, *Ar*), 7.37 (1H, t, 7.5 Hz, *Ar*), 7.30 (1H, t, 8.0 Hz, *Ar*), 6.52 (1H, br s, N*H*), 3.79 (2H, t, 5.6 Hz, C*H*$_2$O), 3.66-3.62 (2H, m, C*H*$_2$N), 2.71 (1H, br s, O*H*), 1.85-1.81 (2H, m, C*H*$_2$).
	δ_C (125.7 MHz, $CDCl_3$) 168.7, 137.6, 133.4, 131.4, 129.5, 127.6, 119.3, 59.6, 37.0, 32.1.
	MS m/z (rel int.): 259/257 (1, M^+), 241/239 (6), 228/226 (8), 214/212 (28), 185/183 (100), 157/155 (22), 105 (10), 76 (24), 50 (10).

	HRMS (ESI-Q-TOF) m/z calcd for $C_{10}H_{12}BrNO_2$ $[M+H]^+$: 258.0124; found: 258.0125.

3d 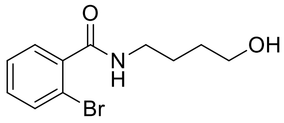	Isolated yield: 0.0766 g (57%). R_F: 0.35 (on silica gel chloroform:2-propanol=9:1). White crystals.
	δ_H (500 MHz, $CDCl_3$) 7.66 (1H, d, 7.9 Hz, *Ar*), 7.46-7.41 (2H, m, *Ar*), 7.38-7.34 (1H, m, *Ar*), 3.64 (2H, t, 8.3 Hz, C*H*$_2$O), 3.41 (2H, t, 6.5 Hz, C*H*$_2$N), 1.75-1.65 (4H, m, C*H*$_2$C*H*$_2$).
	δ_C (125.7 MHz, $CDCl_3$) 169.6, 138.8, 132.8, 130.7, 128.3, 127.2, 119.0, 61.2, 39.3, 29.6, 25.4.
	MS m/z (rel int.): 273/271 (3, M^+), 255/253 (5), 242/240 (10), 228/226 (12), 214/212 (15), 185/183 (100), 157/155 (15), 105 (10), 88 (19), 76 (16).
	HRMS (ESI-Q-TOF) m/z calcd for $C_{11}H_{14}BrNO_2$ $[M+H]^+$: 272.0281; found: 272.0277.

**3e** 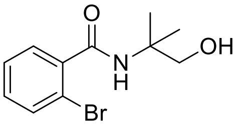	Isolated yield: 0.1111 g (82%). R_F: 0.58 (on silica gel chloroform:2-propanol=92:8). White crystals.
	δ_H (500 MHz, $CDCl_3$) 7.60 (1H, d, 7.7 Hz, *Ar*), 7.53 (1H, d, 7.7 Hz, *Ar*), 7.38 (1H, t, 7.7 Hz, *Ar*), 7.31 (1H, d, 7.7 Hz, *Ar*), 6.02 (1H, br s, N*H*), 3.74 (2H, s, C*H*$_2$), 1.44 (6H, s, C*H*$_3$)
	δ_C (125.7 MHz, $CDCl_3$) 168.3, 138.1, 133.3, 131.4, 129.4, 127.7, 119.3, 70.3, 57.3, 24.6.
	MS m/z (rel int.): 242/240 (47), 202/200 (11), 185/183 (100), 157/155 (14), 105 (11), 76 (15).
	HRMS (ESI-Q-TOF) m/z calcd for $C_{11}H_{14}BrNO_2$ $[M+H]^+$: 272.0281; found: 272.0274.

3g 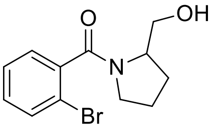	Isolated yield: 0.1033 g (73%). R_F: 0.58 (on silica gel chloroform:2-propanol=92:8). Yellow high viscosity liquid.
	δ_H (500 MHz, $CDCl_3$) 7.58 (1H, d, 7.9 Hz, *Ar*), 7.37 (1H, t, 7.1 Hz, *Ar*), 7.30-7.24 (2H, m, *Ar*), 4.41 (1H, br s, C*H*$_2$O), 4.35 (1H, br s, C*H*$_2$O), 3.79 (1H, br s, C*H*), 3.28-3.23 (2H, m, C*H*$_2$N), 2.18-2.13 (1H, m, CH$_2$C*H*$_2$), 1.90-1.86 (1H, m, CH$_2$C*H*$_2$), 1.83-1.78 (1H, m, CH$_2$C*H*$_2$), 1.73-1.70 (1H, m, CH$_2$C*H*$_2$).
	δ_C (125.7 MHz, $CDCl_3$) 169.6, 139.0, 132. 8, 130.5, 127.9, 127.3, 118.6, 66.3, 61.4, 49.6, 28.6, 24.5.

	MS m/z (rel int.): 267/265 (7), 254/252 (44), 185/183 (100), 157/155 (15), 105 (10), 76 (13).

3h 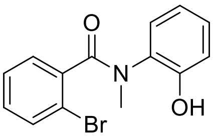	Isolated yield: 0,0979 g (64%). R_F: 0.53 (on silica gel chloroform:ethyl acetate=8:2). Yellow high viscosity liquid.
	δ_H (500 MHz, $CDCl_3$) 7.69 (0.5H, d, 8.1 Hz, *Ar*), 7.48-7.47 (1H, m, *Ar*), 7.43-7.41 (0.5H, m, *Ar*), 7.38-7.35 (0.5H, m, *Ar*), 7.32-7.27 (1H, m, *Ar*), 7.22-7.20 (0.5H, m, *Ar*), 7.18-7.13 (1H, m, *Ar*), 7.06-7.02 (2H, m, *Ar*), 6.80 (0.5H, d, 8.1 Hz, *Ar*), 6.69 (0.5H, t, 7.5 Hz, *Ar*), 3.42 (1.5H, s, CH_3), 3.29 (1.5H, s, CH_3).
	δ_C (125.7 MHz, $CDCl_3$) 170.1, 151.7, 151.3, 138.0, 137.8, 133.0, 132.5, 131.1, 131.0, 130.1, 130.0, 129.7, 128.9, 128.4, 128.1, 127.8, 127.6, 126.7, 124.9, 121.4, 120.6, 120.1, 119.5, 119.0, 116.7, 40.4, 36.2.
	MS m/z (rel int.): 307/305 (24, M^+), 185/183 (100), 157/155 (15), 122 (11).

3f 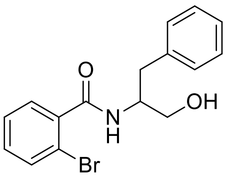	Isolated yield: 0.1228 g (73%). R_F: 0.14 (on silica gel chloroform:2-propanol=97:3). White crystals.
	δ_H (500 MHz, $CDCl_3$) 7.50 (1H, d, 7.8 Hz, *Ar*), 7.32-7.18 (8H, m, *Ar*), 6.70 (1H, br s, N*H*), 4.35-4.32 (1H, m, C*H*), 3.69 (1H, dd, 10.3 Hz, 3.5 Hz, CH_2O), 3.60 (1H, dd, 10.3 Hz, 4.5, Hz, CH_2O), 3.43 (1H, br s. O*H*), 2.96 (2H, d, 7.2 Hz, $CHCH_2$).
	δ_C (125.7 MHz, $CDCl_3$) 168.2, 137.8, 133.2, 131.2, 129.4, 129.1, 128.6, 127.4, 126.6, 119.3, 63.2, 53.3, 36.8.
	MS m/z (rel int.): 335/333 (1, M^+), 304/302 (7), 244/242 (5), 226/224 (10), 202/200 (12), 185/183 (100), 157/155 (16), 134 (12), 105 (18), 91 (25), 76 (15).
	HRMS (ESI-Q-TOF) m/z calcd for $C_{16}H_{16}BrNO_2$ $[M+H]^+$: 334.0437; found: 334.0426.

3i 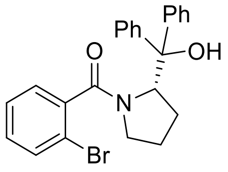	Isolated yield: 0.0435 g (20%). R_F: 0.42 (on silica gel chloroform:ethyl acetate=95:5). Yellow high viscosity liquid.
	δ_H (500 MHz, $CDCl_3$) 7.64 (2H, d, 7.5 Hz, Ar), 7.55 (1H, d, 8.0 Hz, *Ar*), 7.49 (2H, d, 7.5 Hz, *Ar*), 7.42-7.29 (8H, m, *Ar*), 7.22 (1H, t, 7.5 Hz, *Ar*), 5.32 (1H, t, 7.9 Hz, C*H*), 3.13-3.09 (1H, m, NCH_2), 2.66 (1H, br s, CH_2), 2.21 (1H, br s, CH_2), 2.05 (1H, br s, CH_2),1.66 (1H, br s, CH_2), 1.36(1H, br s, CH_2).
	δ_C (125.7 MHz, $CDCl_3$) 170.5, 145.3, 143.0, 138.8, 132.7, 130.4, 128.0, 127.8, 127.6, 127.4, 118.1, 81.9, 68.1, 50.8, 30.4, 24.1.

	MS m/z (rel int.): 253/255 (70), 254/252 (64), 183/185 (100), 174 (31), 157/155 (10), 105 (63), 77 (39).
	HRMS (ESI-Q-TOF) m/z calcd for $C_{24}H_{20}BrNO$ $[M+H]^+$: 418.0801; found: 418.0786.

**3j** 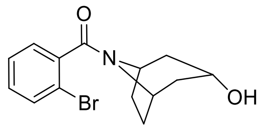	Isolated yield: 0.1066 g (69%). R_F: 0.54 (on silica gel chloroform:ethyl acetate:2-propanol=70:20:10). Yellowish brown high viscosity liquid.
	δ_H (500 MHz, $CDCl_3$) 7.62-7.59 (1H, m, *Ar*), 7.36 (1H, t, 7.5 Hz, *Ar*), 7.28-7.25 (2H, m, *Ar*), 4.85 (1H, br, s, C*H*O), 4.20-4.18 (1H, m, C*H*N), 3.67 (1H, br s, C*H*N), 2.31-1.70 (8H, m, C*H*$_2$).
	δ_C (125.7 MHz, $CDCl_3$) 164.2, 138.6, 132.9, 130.2, 128.0, 127.6, 119.0, 64.9, 55.5, 50.7, 40.2, 39.1, 28.7, 27.5.
	MS m/z (rel int.): 311/309 (27), 230 (33), 202 (22), 185/183 (100), 157/155 (24), 126 (69), 110 (32), 68 (53).
	HRMS (ESI-Q-TOF) m/z calcd for $C_{14}H_{16}BrNO_2$ $[M+H]^+$: 310.0437; found: 310.0429.

4c 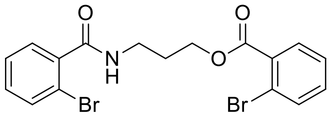	Isolated yield: 0.0785 g (36%). R_F: 0.56 (on silica gel chloroform:2-propanol=95:5). Reddish brown high viscosity liquid.
	δ_H (500 MHz, $CDCl_3$) 7.80 (1H, dd, 7.5 Hz, 2.2 Hz, *Ar*), 7.66 (1H, dd, 7.5 Hz, 2.2 Hz, *Ar*), 7.58 (1H, d, 7.5 Hz, *Ar*), 7.51 (1H, dd, 7.5 Hz, 2.2 Hz, *Ar*), 7.39-7.33 (3H, m, *Ar*), 7.29-7.24 (1H, m, *Ar*), 6.40 (1H, br s, N*H*), 4.52 (2H, t, 6.0 Hz, C*H*$_2$O), 3.67-3.63 (2H, m, C*H*$_2$NH), 2.17-2.12 (2H, C*H*$_2$C*H*$_2$).
	δ_C (125.7 MHz, $CDCl_3$) 167.9, 166.5, 137.9, 134.3, 133.3, 132.7, 132.2, 131.3, 131.2, 129.5, 127.6, 127.3, 121.5, 119.3, 63.2, 37.0, 28.6.
	MS m/z (rel int.):258/256 (11), 241/239 (37), 185/183 (100), 157/155 (17), 132 (8), 105 (12), 76 (18), 56 (40).
	HRMS (ESI-Q-TOF) m/z calcd for $C_{17}H_{15}Br_2NO_3$ $[M+H]^+$: 439.9491; found: 439.9482.

4d 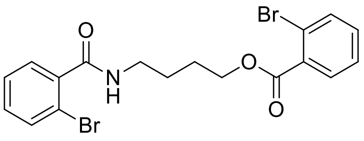	Isolated yield: 0.1002 g (44%). R_F: 0.58 (on silica gel chloroform:2-propanol=95:5). Reddish brown high viscosity liquid.
	δ_H (500 MHz, $CDCl_3$) 7.79 (1H, d, 7.5 Hz, *Ar*), 7.67 (1H, d, 7.5 Hz, *Ar*), 7.58 (1H, d, 8.0 Hz, *Ar*), 7.51 (1H, d, 78.0 Hz, *Ar*), 7.39-7.32 (3H, m, *Ar*), 7.27 (1H, t, 7.9 Hz, *Ar*), 6.24 (1H, br s, N*H*), 4.40 (2H, t, 6.3 Hz, C*H*$_2$O), 3.56-3.52 (2H, m, C*H*$_2$N), 1.95-1.90 (2H, m, C*H*$_2$CH$_2$), 1.85-1.80 (2H, m, C*H*$_2$CH$_2$).

	δ$_C$ (125.7 MHz, $CDCl_3$) 167.8, 166.3, 137.9, 134.3, 133.3, 132.5, 132.4, 131.3, 131.2, 129.5, 127.6, 127.2, 121.5, 119.2, 65.2, 39.6, 26.3, 26.2.
	MS m/z (rel int.): 457/455/453 (5, M^+), 272/270 (26), 255/253 (10), 202/200 (15), 185/183 (100), 157/155 (17), 105 (25), 75 (16), 70 (33).
	HRMS (ESI-Q-TOF) m/z calcd for $C_{18}H_{17}Br_2NO_3$ $[M+H]^+$: 453.9648; found: 453.9635.

4e 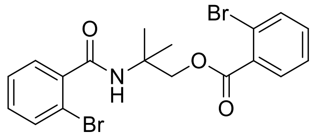	Isolated yield: 0.1539 g (68%). R$_F$: 0.74 (on silica gel chloroform:2-propanol=95:5). Reddish brown crystals.
	δ$_H$ (500 MHz, $CDCl_3$) 7.83 (1H, dd, 7.5 Hz, 2.0 Hz, *Ar*), 7.66 (1H, d, 7.5 Hz, *Ar*), 7.55 (1H, d, 7.5 Hz, *Ar*), 7.49 (1H, d, 7.5 Hz, *Ar*), 7.40-7.30 (3H, m, *Ar*), 7.24 (1H, t, 8.0 Hz, *Ar*), 6.08 (1H, br s, N*H*), 4.58 (2H, s, CH_2), 1.60 (6H, s, CH_3).
	δ$_C$ (125.7 MHz, $CDCl_3$) 167.3, 166.2, 138.5, 134.4, 133.2, 132.8, 132.1, 131.6, 131.1, 129.3, 127.5, 127.3, 121.5, 119.1, 70.2, 54.3, 24.2.
	MS m/z (rel int.): 255/253 (7), 242/240 (73), 185/183 (100), 157/155 (15), 105 (17), 76 (16).
	HRMS (ESI-Q-TOF) m/z calcd for $C_{18}H_{17}Br_2NO_3$ $[M+H]^+$: 453.9648; found: 453.9642.

4g 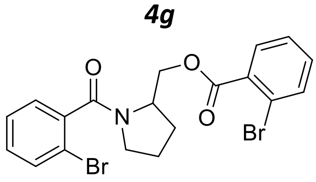	Isolated yield: 0.0582 g (25%). R$_F$: 0.65 (on silica gel chloroform:ethyl acetate=8:2). Orange high viscosity liquid.
	δ$_H$ (500 MHz, $CDCl_3$) 7.86 (1H, d, 7.5 Hz, *Ar*), 7.69-7.65 (1H, m, *Ar*), 7.57 (1H, d, 7.9 Hz, *Ar*), 7.41-7.24 (5H, m, *Ar*), 4.72-4.62 (2H, m, CH_2O), 4.10-3.68 (1H, m, NC*H*, NCH_2), 3.32-3.23 (2H, m, NC*H*, NCH_2), 2.21-2.16 (1H, m, CH_2), 2.10-2.02 (2H, m, CH_2), 1.92-1.88 (1H, m, CH_2).
	δ$_C$ (125.7 MHz, $CDCl_3$) 167.9, 166.1, 139.3, 134.5, 132.8, 132.6, 132.2, 131.5, 131.2, 130.5, 130.3, 127.8, 127.6, 127.3, 121.6, 118.8, 65.7, 64.9, 55.6, 48.7, 46.1, 39.1, 29.1, 27.9, 24.3, 22.5.
	MS m/z (rel int.): 284/282 (5), 254/252 (50), 185/183 (100), 157/155 (13), 105 (19), 76 (11).
	HRMS (ESI-Q-TOF) m/z calcd for $C_{19}H_{17}Br_2NO_3$ $[M+H]^+$: 465.9648; found: 465.9634.

4f	Isolated yield: 0.0953 g (37%). R_F: 0.72 (on silica gel chloroform:ethyl acetate=9:1). Brownish white crystals.
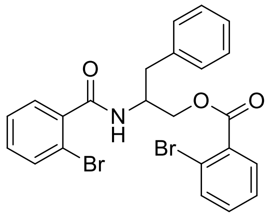	δ_H (500 MHz, $CDCl_3$) 7.87 (1H, dd, 7.5 Hz, 1.8 Hz, *Ar*), 7.70 (1H, d, 7.8 Hz, *Ar*), 5.57 (1H, d, 7.8 Hz, *Ar*), 7.43-7.40 (2H, m, *Ar*), 7.38-7.31 (6H, m, *Ar*), 7.29-7.25 (2H, m, *Ar*), 6.32 (1H, s, N*H*), 4.86-4.80 (1H, m, NCH_2), 4.50 (1H, dd, 11.0 Hz, 4.0 Hz, CH_2O), 4.46 (1H, dd, 11.0 Hz, 4.7 Hz, CH_2O), 3.16 (1H, dd, 13.7 Hz, 6.7 Hz, ArCH_2), 3.09 (1H, dd, 13.7 Hz, 8.0 Hz, ArCH_2).
	δ_C (125.7 MHz, $CDCl_3$) 167.3, 166.1, 137.6, 136.8, 134.4, 133.4, 132.9, 131.8, 131.3, 129.44, 129.36, 128.8, 127.6, 127.4, 127.0, 121.6, 119.2, 65.8, 50.2, 37.6.
	MS m/z (rel int.): 226/224 (100), 198/196 (21), 171/169 (26), 146 (5), 117 (10), 91 (16), 65 (7).
	HRMS (ESI-Q-TOF) m/z calcd for $C_{23}H_{19}Br_2NO_3$ $[M+H]^+$: 515.9804; found: 515.9789.

8b	Isolated yield: 0.0513 g (48%). R_F: 0.69 (on silica gel chloroform:2-propanol=9:1). Yellow high viscosity liquid.
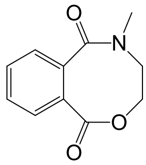	δ_H (500 MHz, $CDCl_3$) 7.76 (1H, d, 8.0 Hz, *Ar*), 7.66 (1H, t, 8.0 Hz, *Ar*), 7.61-7.56 (2H, m, *Ar*), 4.43-4.38 (1H, m, OCH_2), 4.17-4.14 (1H, m, OCH_2), 3.72-3.67 (1H, m, NCH_2), 3.34-3.30 (1H, m, NCH_2), 3.22 (3H, s, CH_3).
	δ_C (125.7 MHz, $CDCl_3$) 170.6, 169.9, 135.9, 132.9, 130.3, 130.1, 129.6, 128.7, 64.7, 48.5, 34.8.
	MS m/z (rel int.): 205 (6, M^+), 175 (11), 160 (26), 132 (50), 104 (100), 76 (32), 57 (14), 42 (20).
	HRMS (ESI-Q-TOF) m/z calcd for $C_{11}H_{11}NO_3$ $[M+H]^+$: 206.0812; found: 206.0820.

8g	Isolated yield: 0.0294 g (26%). R_F: 0.42 (on silica gel chloroform:2-propanol=95:5). Yellowish brown high viscosity liquid.
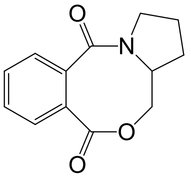	δ_H (500 MHz, $CDCl_3$) 7.66-7.53 (4H, m, *Ar*), 4.22 (1H, dd, 5.6 Hz, 13.5 Hz, CH_2O), 4.12-4.09 (1H, m, CH_2O), 3.97-3.92 (1H, m, C*H*), 3.78-3.74 (1H, m, NCH_2), 3.69-3.65 (1H, m, NCH_2), 2.17-2.03 (3H, m, CH_2CH_2), 1.19-1.88 (1H, m, CH_2CH_2).
	δ_C (125.7 MHz, $CDCl_3$) 170.6, 167.7, 134.8, 131.8, 130.8, 129.1, 128.8, 128.0, 68.9, 57.0, 47.8, 32.3, 21.8.
	MS m/z (rel int.): 231 (14, M^+), 201 (40), 163 (39), 145 (20), 1332 (78), 104 (100), 76 (36).
	HRMS (ESI-Q-TOF) m/z calcd for $C_{13}H_{13}NO_3$ $[M+H]^+$: 232.0890; found: 232.0966.

8i 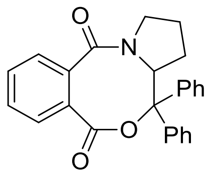	Isolated yield: 0.0349 g (36%). R_F: 0.45 (on silica gel chloroform:ethyl acetate=9:1). Yellowish white high viscosity liquid.
	δH (500 MHz, $CDCl_3$) 7.64 (2H, d, 7.0 Hz, *Ar*), 7.50 (2H, d, 7.5 Hz, *Ar*), 7.44-7.35 (5H, m, *Ar*), 7.32-7.27 (3H, m, *Ar*), 6.97 (1H, br s, *Ar*), 6.74-6.72 (1H, m, *Ar*), 5.26 (1H, t, 8.3 Hz, C*H*), 3.30 (1H, t, 8.0 Hz, NCH_2), 2.77-2.72 (1H, m, NCH_2), 2.25-2.18 (1H, m, CH_2CH_2), 2.01-1.94 (1H, m, CH_2CH_2), 1.74-1.68 (1H, m, CH_2CH_2), 1.42-1.36 (1H, m, CH_2CH_2).
	δC (125.7 MHz, $CDCl_3$) 172.0, 145.4, 143.1, 135.3, 129.0, 128.06, 128.01 127.9, 127.5, 127.4, 127.3, 126.2, 82.1, 67.9, 51.8, 30.4, 24.1.
	HRMS (ESI-Q-TOF) m/z calcd for $C_{25}H_{21}NO_3$ $[M+H]^+$: 384.1594; found: 384.1582.

9b 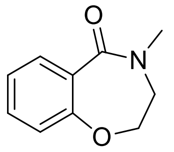	Isolated yield: 0.0250 g (28%). R_F: 0.48 (on silica gel chloroform:2-propanol=95:5). Yellowish brown high viscosity liquid.
	δH (500 MHz, $CDCl_3$) 7.84 (1H, d, 7.4 Hz, *Ar*), 7.43 (1H, t, 7.4 Hz, *Ar*), 7.18 (1H, t, 7.4 Hz, *Ar*), 7.02 (1H, d, 7.4 Hz, *Ar*), 4.42 (2H, t, 5.2 Hz, OCH_2), 3.55 (2H, t, 5.2 Hz, NCH_2), 3.25 (3H, s, CH_3).
	δC (125.7 MHz, $CDCl_3$) 168.6, 153.8, 132.7, 131.1, 127.2, 123.5, 121.3, 72.5, 48.5, 35.5.
	MS m/z (rel int.): 177 (15, M^+), 162 (100), 120 (81), 92 (46), 56 (34), 42 (21).
	HRMS (ESI-Q-TOF) m/z calcd for $C_{10}H_{11}NO_2$ $[M+H]^+$: 178.0863; found: 178.0855.

**9c**	Isolated yield: 0.0439 g (39%). R_F: 0.78 (on silica gel chloroform:ethyl acetate=9:1). Yellowish brown high viscosity liquid.
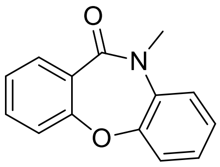	δ_H (500 MHz, $CDCl_3$) 7.91 (1H, d, 8.0 Hz, *Ar*), 7.48 (1H, t, 7.3 Hz, *Ar*), 7.32-7.15 (6H, m, *Ar*), 3.61 (3H, s, CH_3).
	δ_C (125.7 MHz, $CDCl_3$) 166.5, 160.6, 153.7, 136.1, 133.5, 132.3, 126.3, 126.2, 125.8, 125.2, 122.6, 121.5, 119.8, 36.8.
	MS m/z (rel int.): 225 (100, M^+), 208 (15), 196 (20), 182 (23), 168 (25), 127 (9), 77 (10).
	HRMS (ESI-Q-TOF) m/z calcd for $C_{14}H_{11}NO_2$ $[M+H]^+$: 226.0863; found: 226.0859.

Spectroscopic data (^{1}H, ^{13}C, MS) of prepared compounds

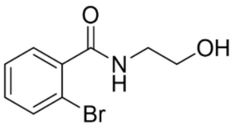
O
N
H
OH
Br

3a

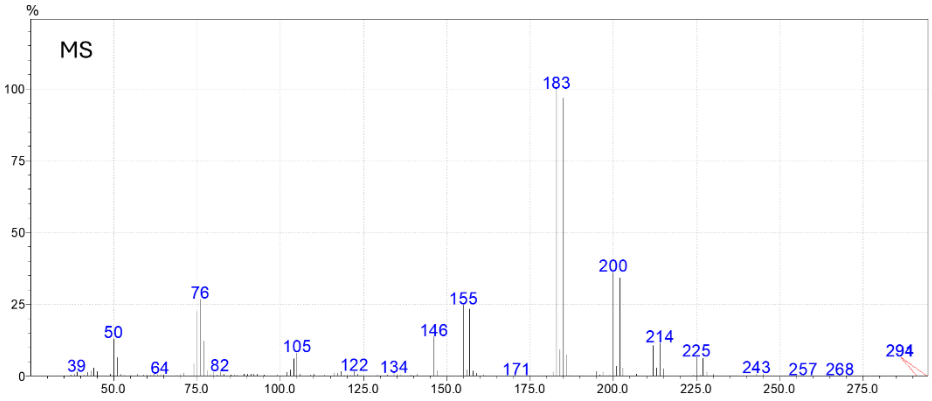
%
MS
100
75
50
25
0
183
200
76
155
50
146
105
214
225
294
39
64
82
122
134
171
243
257
268
50.0
75.0
100.0
125.0
150.0
175.0
200.0
225.0
250.0
275.0

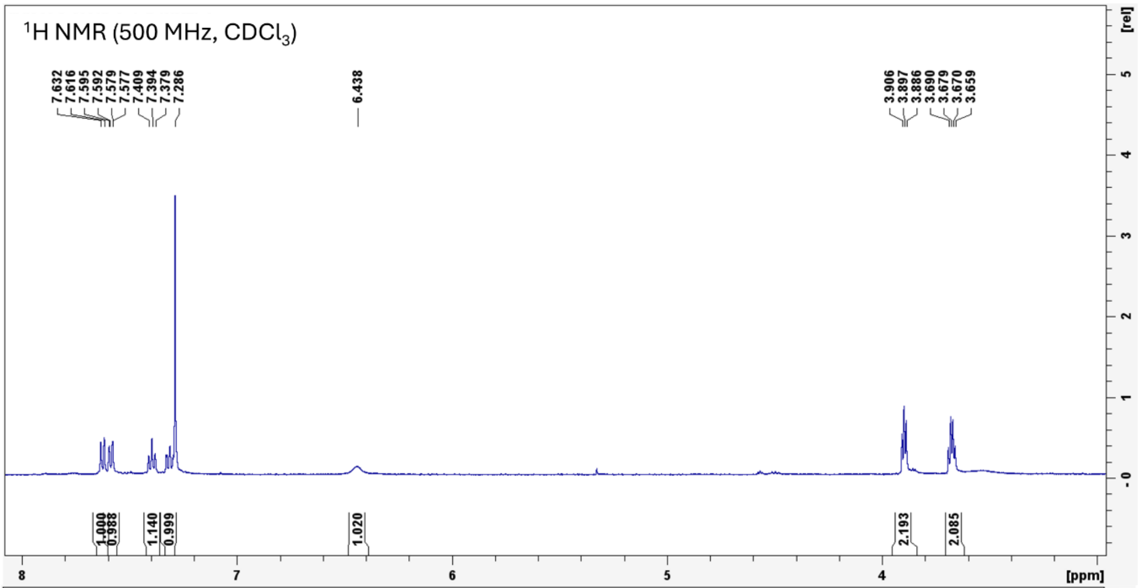
1H NMR (500 MHz, CDCl3)
7.632
7.616
7.595
7.592
7.579
7.577
7.409
7.394
7.379
7.286
6.438
3.906
3.897
3.886
3.690
3.679
3.670
3.659
1.000
0.988
1.140
0.999
1.020
2.193
2.085
[rel]
[ppm]

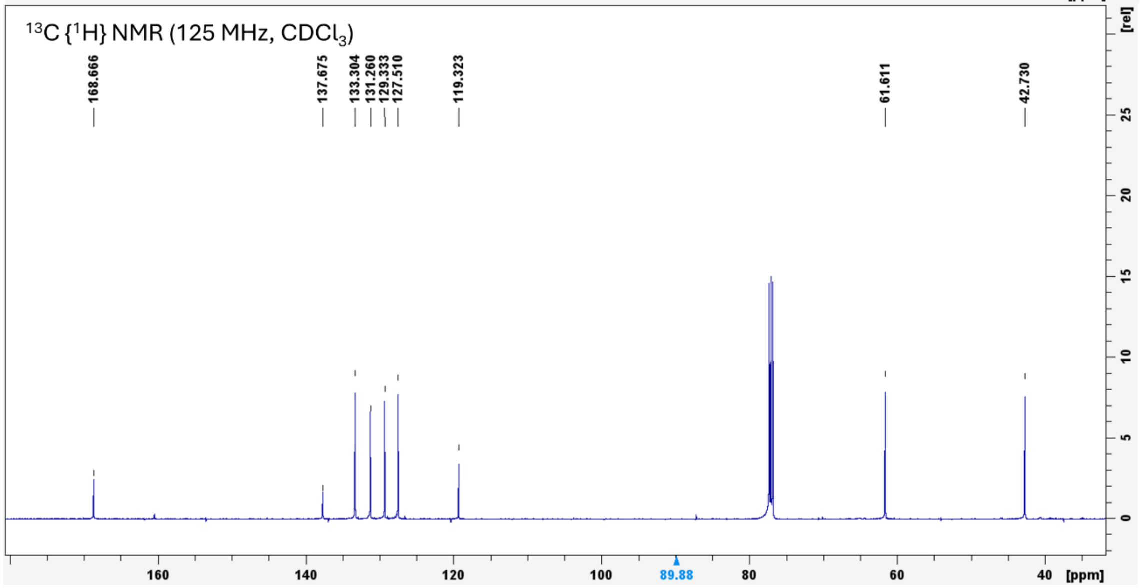
13C {1H} NMR (125 MHz, CDCl3)
168.666
137.675
133.304
131.260
129.333
127.510
119.323
61.611
42.730
89.88
[rel]
[ppm]

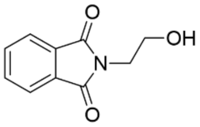
O
N
OH
O

4a

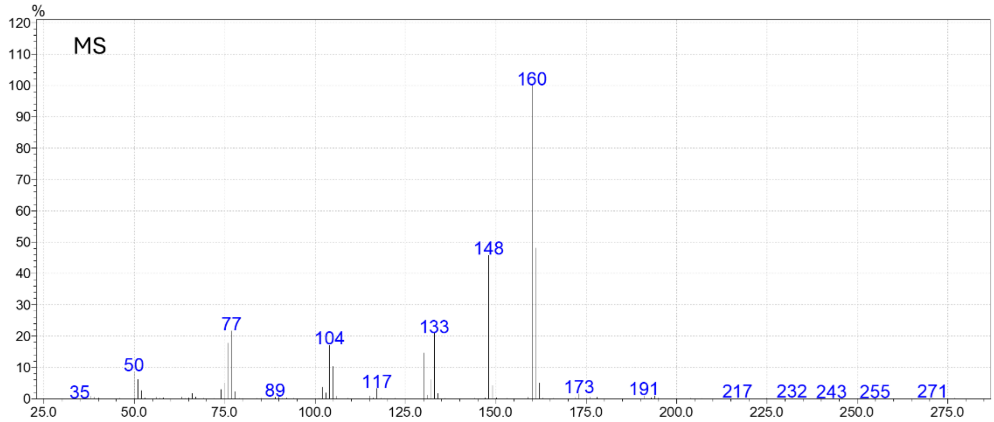
MS
%
160
148
133
104
77
50
35
89
117
173
191
217
232
243
255
271

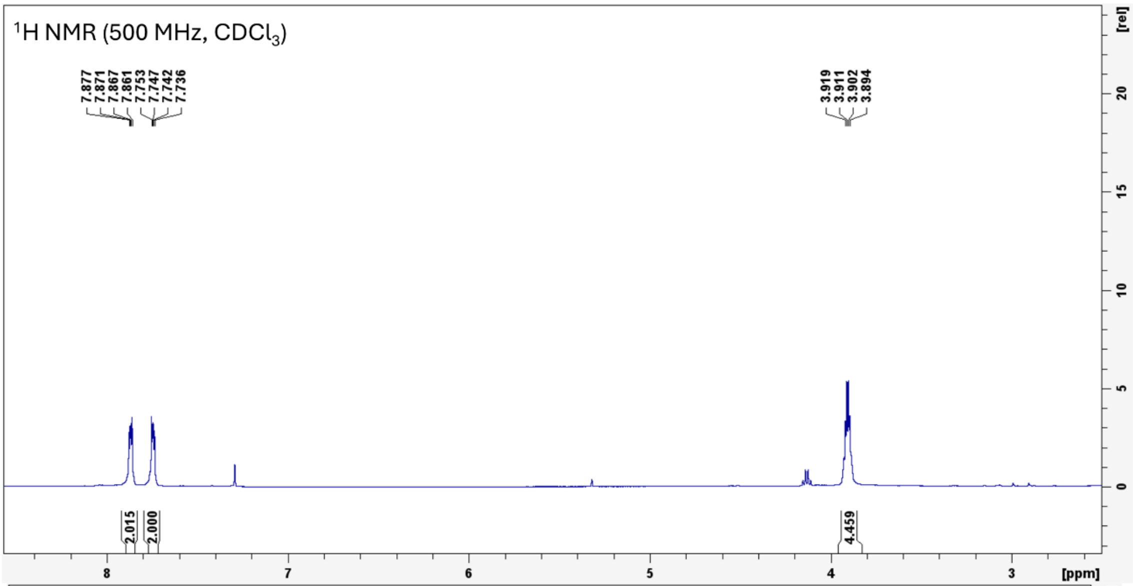
1H NMR (500 MHz, CDCl3)
7.877
7.871
7.867
7.861
7.753
7.747
7.742
7.736
3.919
3.911
3.902
3.894
2.015
2.000
4.459
[ppm]
[rel]

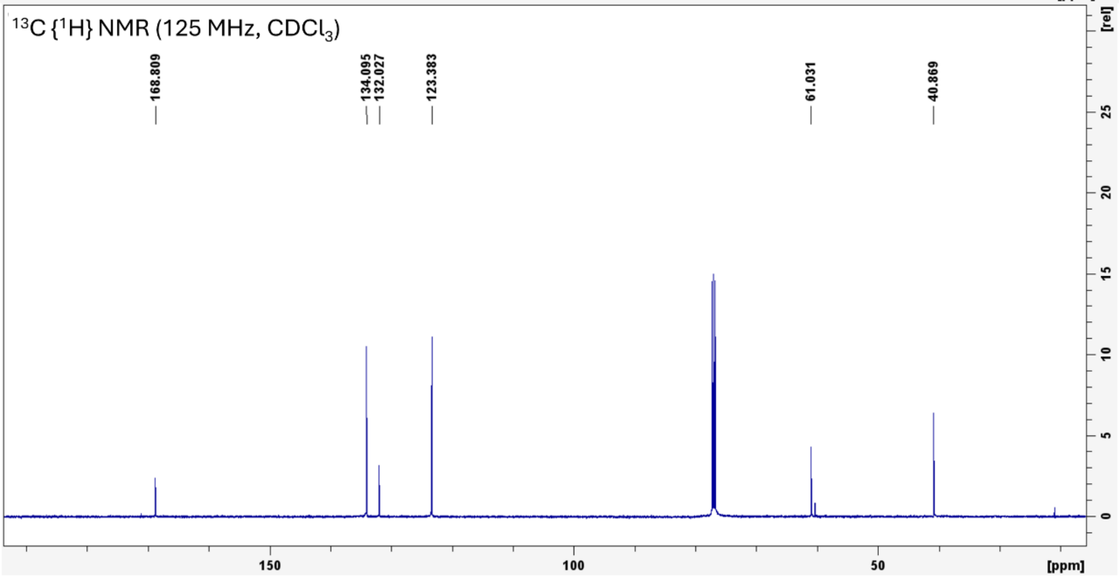
13C {1H} NMR (125 MHz, CDCl3)
168.809
134.095
132.027
123.383
61.031
40.869
[ppm]
[rel]

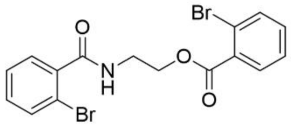

Br
O
N
H
O
O
Br

6a

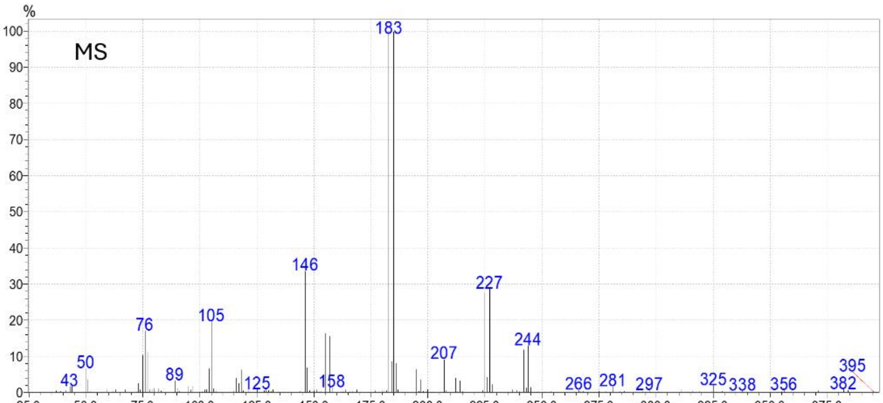

MS
%
183
146
227
244
105
76
207
50
43
89
125
158
266
281
297
325
338
356
382
395

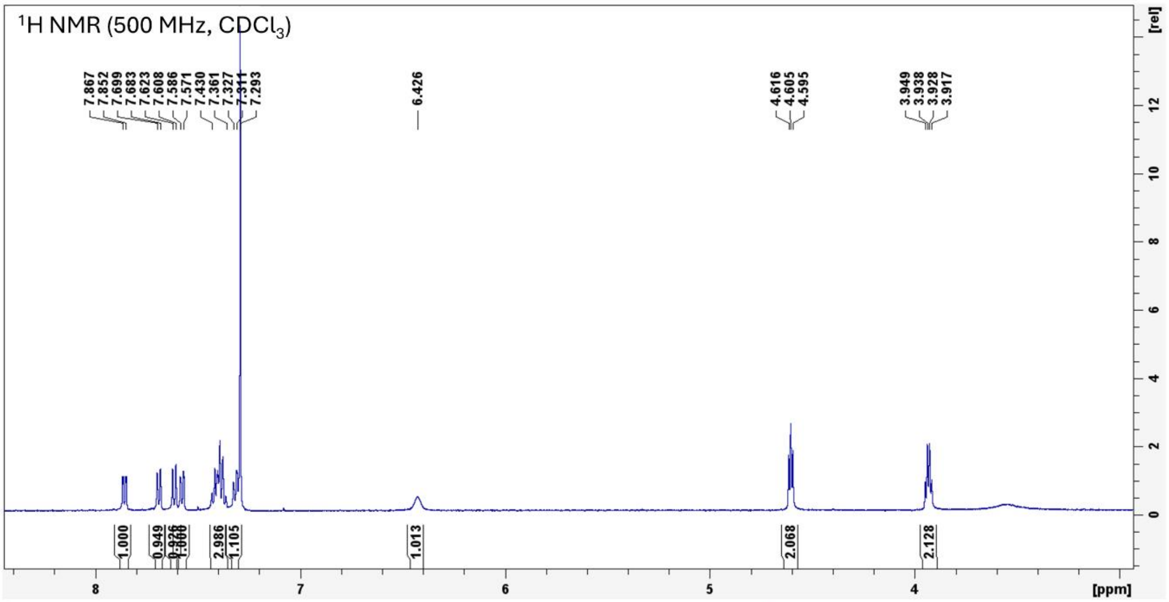

1H NMR (500 MHz, CDCl3)
7.867
7.852
7.699
7.683
7.623
7.608
7.586
7.571
7.430
7.361
7.327
7.311
7.293
6.426
4.616
4.605
4.595
3.949
3.938
3.928
3.917
1.000
0.949
2.986
1.105
1.013
2.068
2.128
[rel]
[ppm]

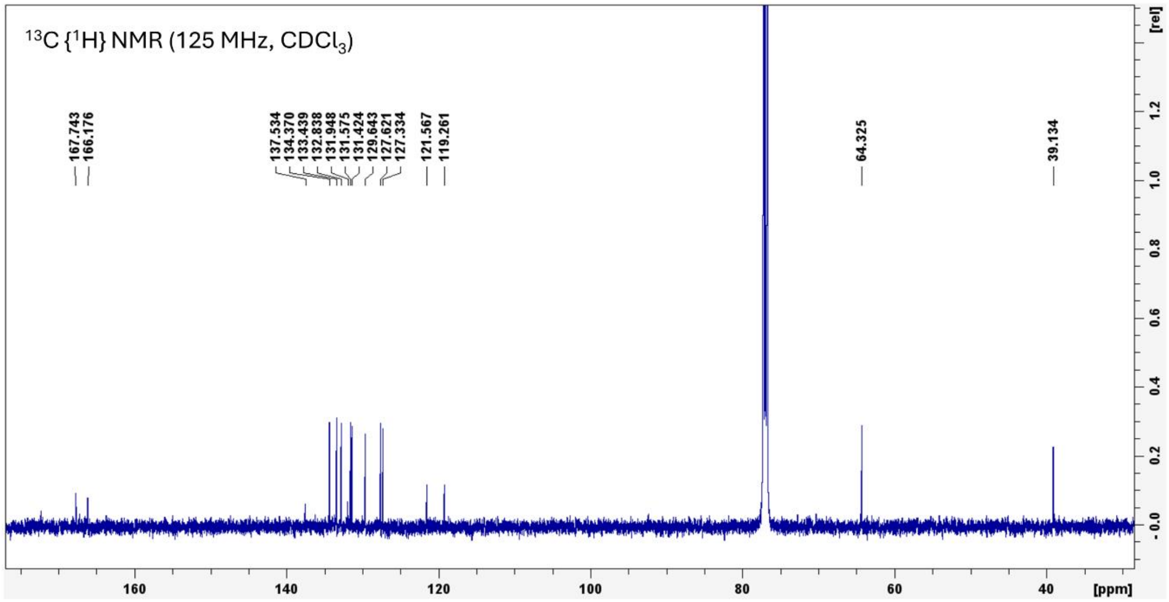

13C {1H} NMR (125 MHz, CDCl3)
167.743
166.176
137.534
134.370
133.439
132.838
131.948
131.575
131.424
129.643
127.621
127.334
121.567
119.261
64.325
39.134
[rel]
[ppm]

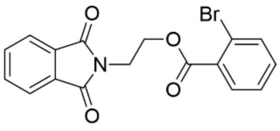
O
Br
N
O
O
O

7a

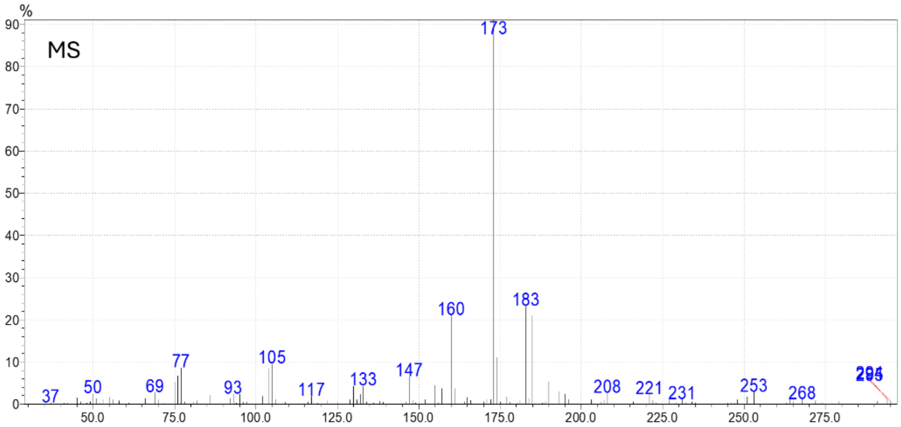
MS
%
173
160
183
37
50
69
77
93
105
117
133
147
208
221
231
253
268
50.0
75.0
100.0
125.0
150.0
175.0
200.0
225.0
250.0
275.0

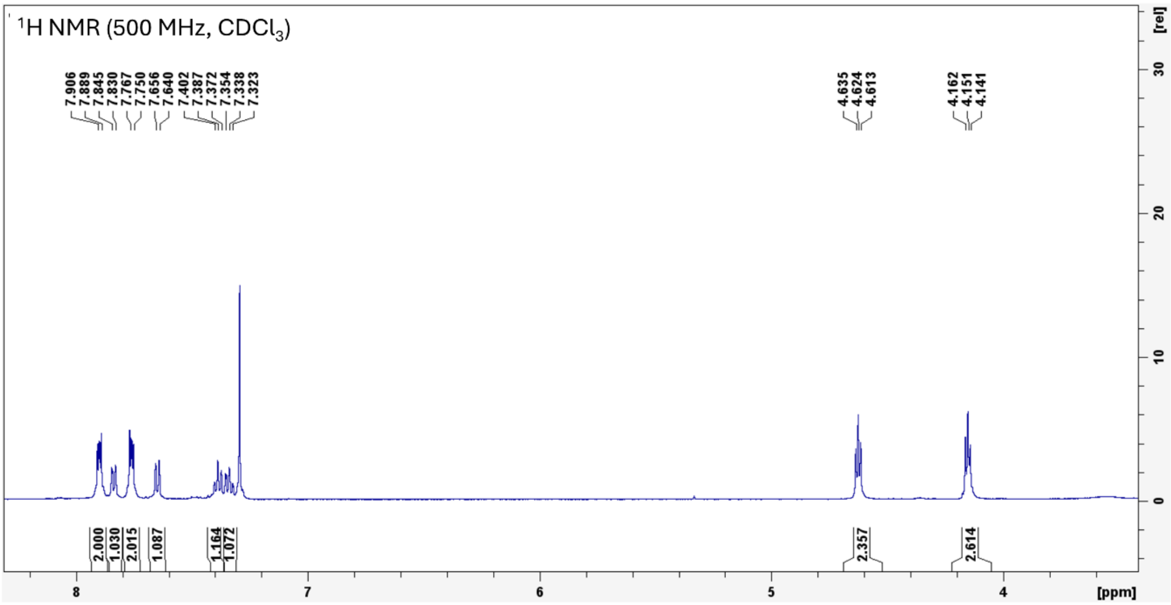
^{1}H NMR (500 MHz, CDCl$_3$)
7.906
7.889
7.845
7.830
7.767
7.750
7.656
7.640
7.402
7.387
7.372
7.354
7.338
7.323
4.635
4.624
4.613
4.162
4.151
4.141
2.000
1.030
2.015
1.087
1.164
1.072
2.357
2.614
[ppm]
[rel]

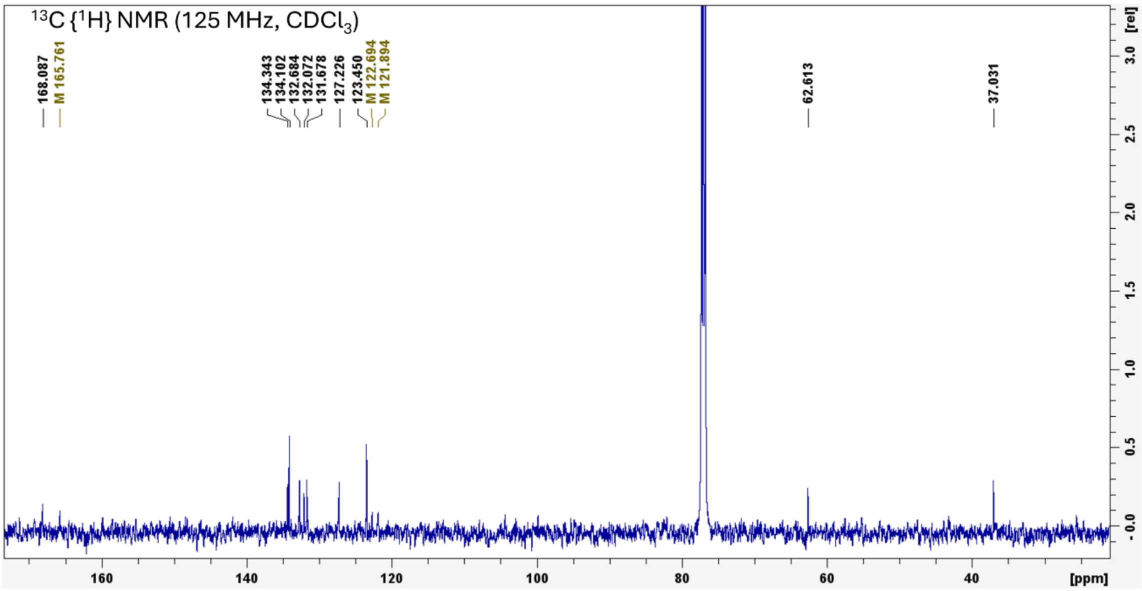
^{13}C {^{1}H} NMR (125 MHz, CDCl$_3$)
168.087
M 165.761
134.343
134.102
132.684
132.072
131.678
127.226
123.450
M 122.694
M 121.894
62.613
37.031
[ppm]
[rel]

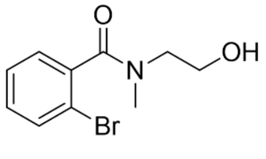
O
N
OH
Br

3b

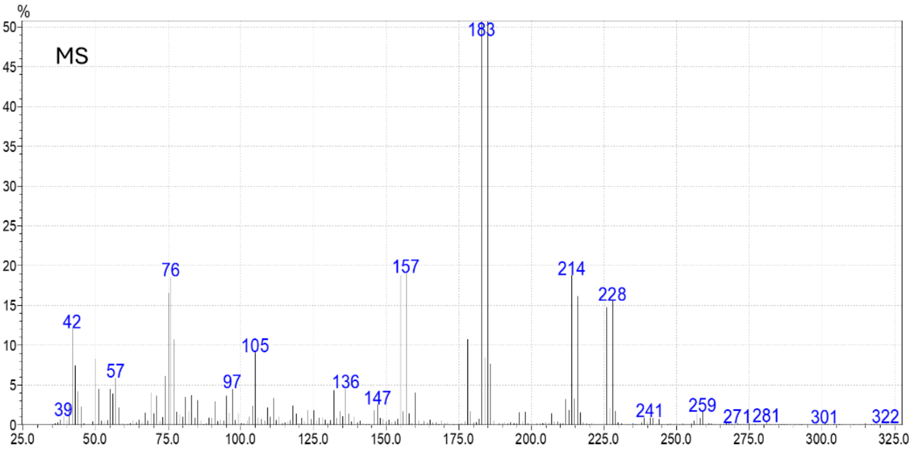
%
MS
183
76
157
214
228
42
105
57
97
136
147
39
241
259
271281
301
322

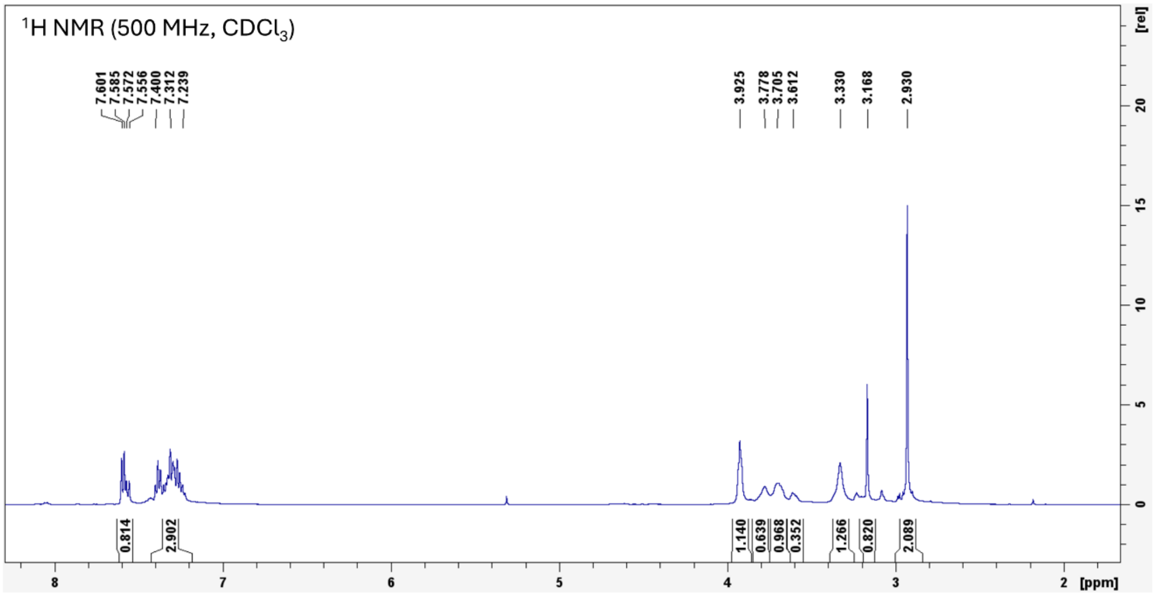
1H NMR (500 MHz, CDCl3)
7.601
7.585
7.572
7.556
7.400
7.312
7.239
3.925
3.778
3.705
3.612
3.330
3.168
2.930
0.814
2.902
1.140
0.639
0.968
0.352
1.266
0.820
2.089
[ppm]
[rel]

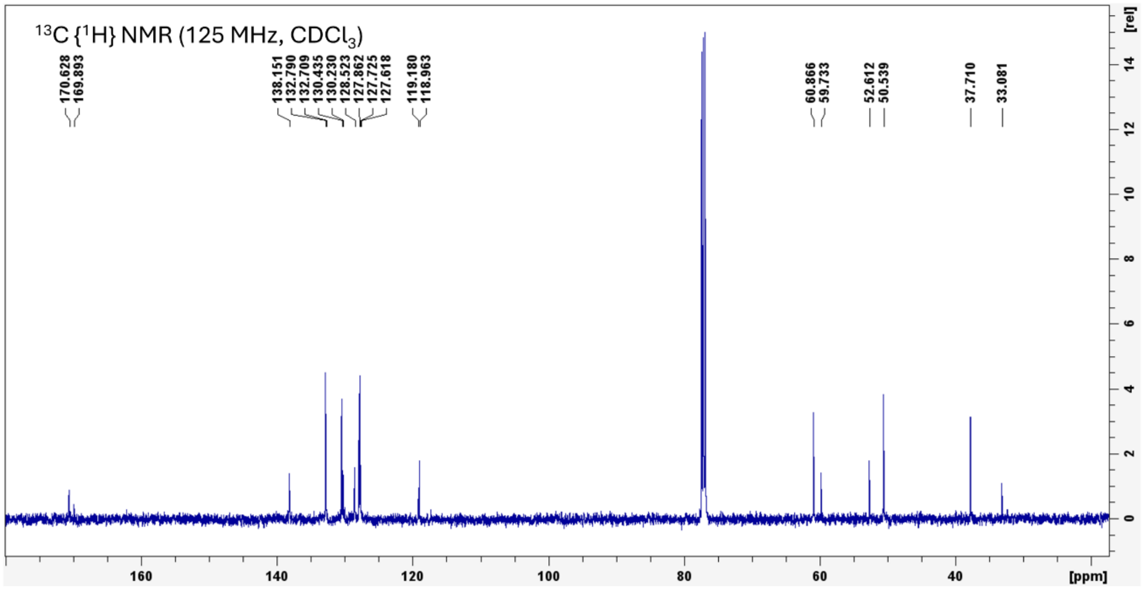
13C {1H} NMR (125 MHz, CDCl3)
170.628
169.893
138.151
132.790
132.709
130.435
130.230
128.523
127.862
127.725
127.618
119.180
118.963
60.866
59.733
52.612
50.539
37.710
33.081
[ppm]
[rel]

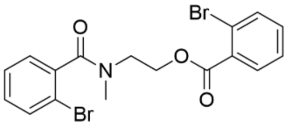
O
Br
N
O
O
Br

6b

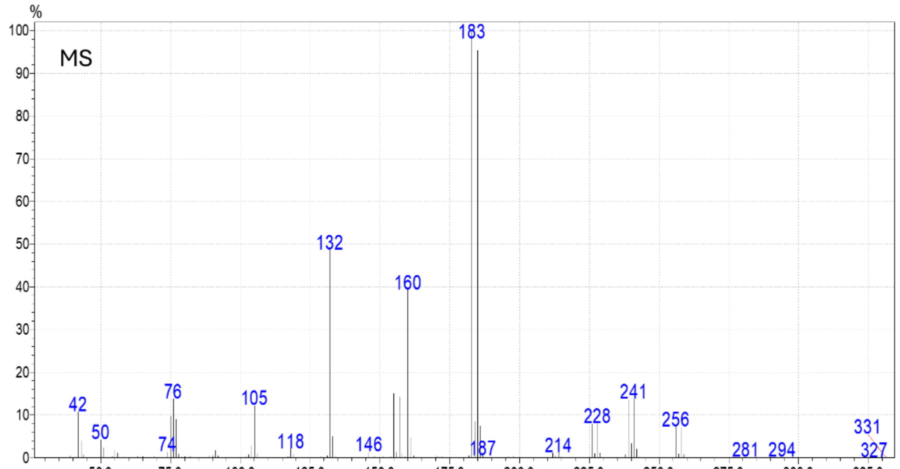
MS
%
183
132
160
42
50
74
76
105
118
146
187
214
228
241
256
281
294
331
327

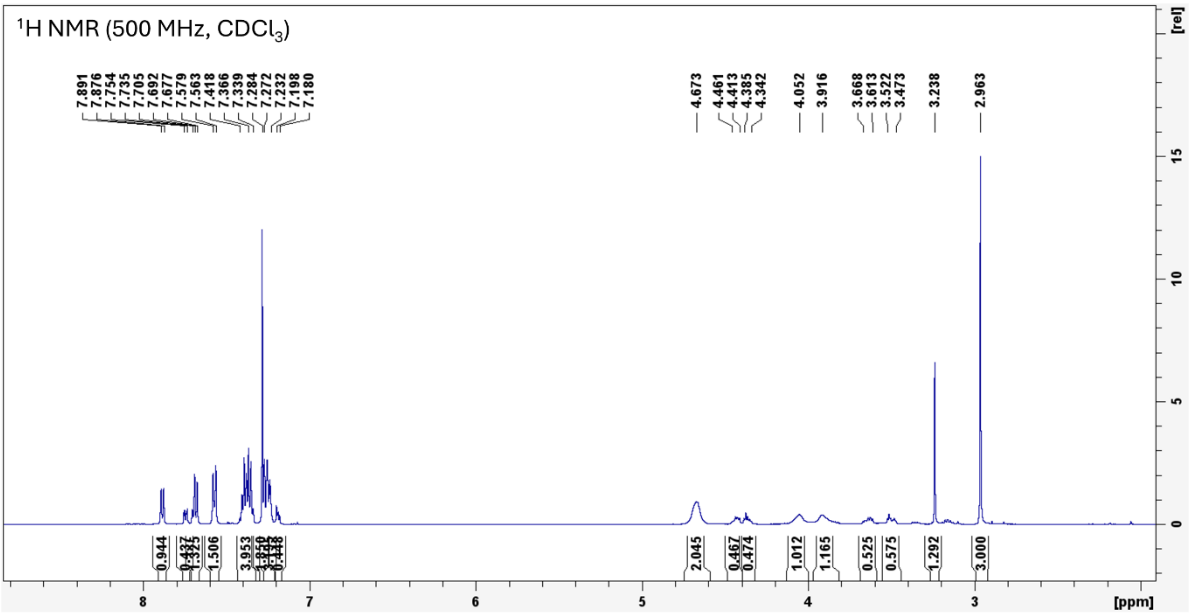
^{1}H NMR (500 MHz, $CDCl_3$)
7.891
7.876
7.754
7.735
7.705
7.692
7.677
7.579
7.563
7.418
7.366
7.339
7.284
7.272
7.232
7.198
7.180
4.673
4.461
4.413
4.385
4.342
4.052
3.916
3.668
3.613
3.522
3.473
3.238
2.963
0.944
1.506
3.953
2.045
0.467
0.474
1.012
1.165
0.525
0.575
1.292
3.000
[ppm]
[rel]

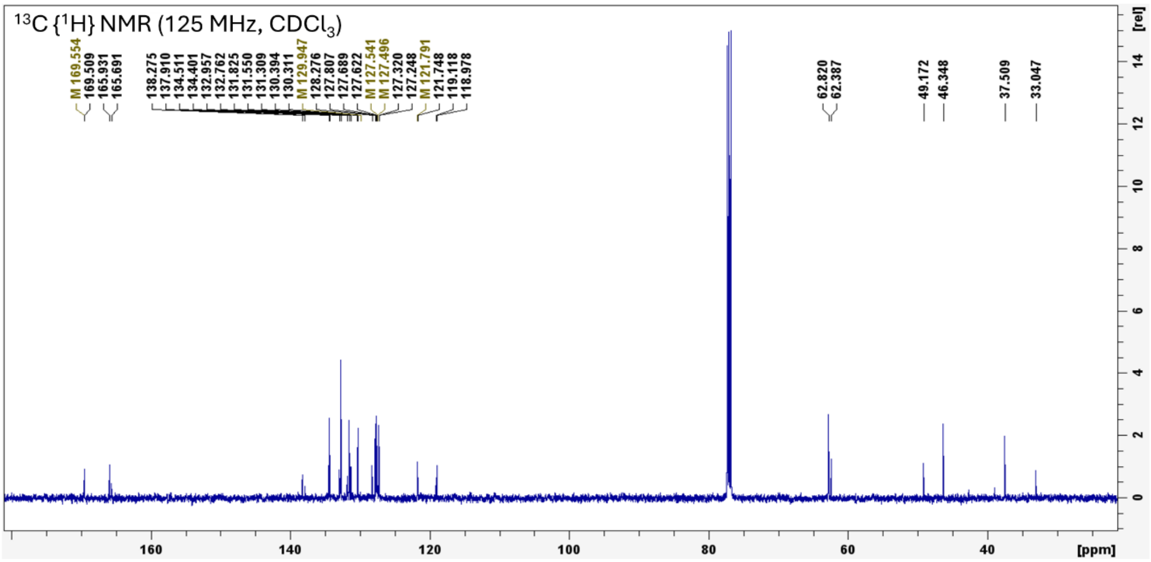
^{13}C {^{1}H} NMR (125 MHz, $CDCl_3$)
M 169.554
169.509
165.931
165.691
138.275
137.910
134.511
134.401
132.957
132.762
131.825
131.550
131.309
130.394
130.311
M 129.947
128.276
127.807
127.689
127.622
M 127.541
M 127.496
127.320
127.248
M 121.791
121.748
119.118
118.978
62.820
62.387
49.172
46.348
37.509
33.047
[ppm]
[rel]

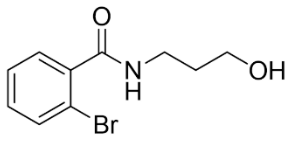
O
N
H
OH
Br

3c

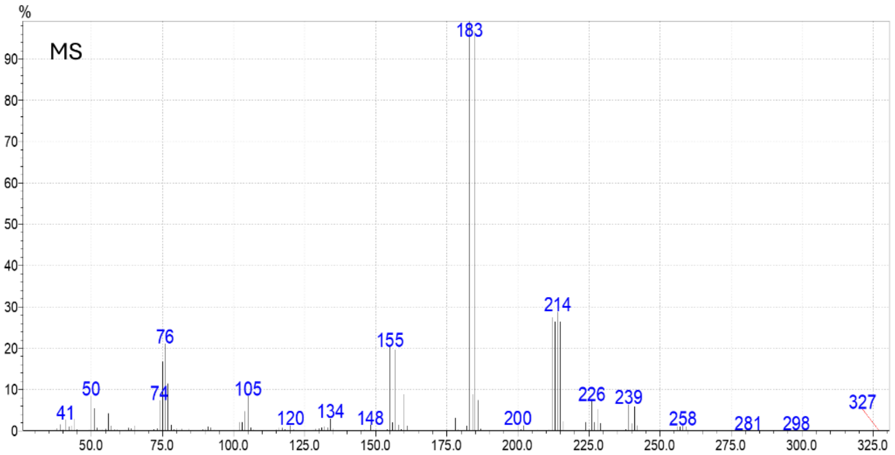
MS
%
183
214
76
155
50
74
105
41
120
134
148
200
226
239
258
281
298
327
50.0
75.0
100.0
125.0
150.0
175.0
200.0
225.0
250.0
275.0
300.0
325.0

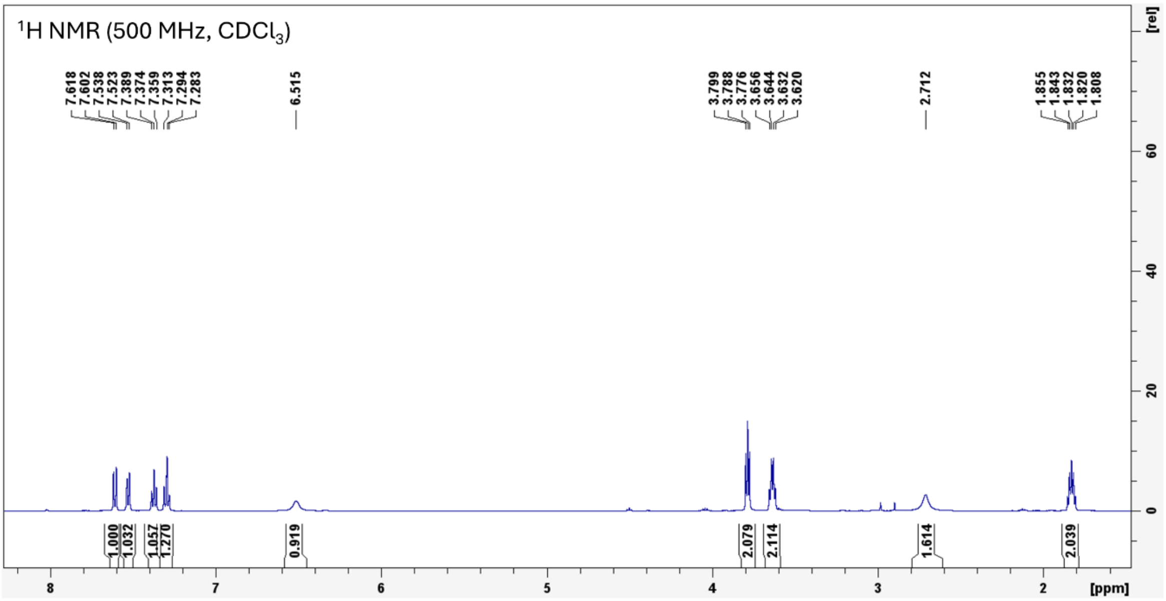
^{1}H NMR (500 MHz, CDCl$_3$)
7.618 7.602 7.538 7.523 7.389 7.374 7.359 7.313 7.294 7.283
6.515
3.799 3.788 3.776 3.656 3.644 3.632 3.620
2.712
1.855 1.843 1.832 1.820 1.808
1.000 1.032 1.057 1.270 0.919 2.079 2.114 1.614 2.039
[ppm]
[rel]

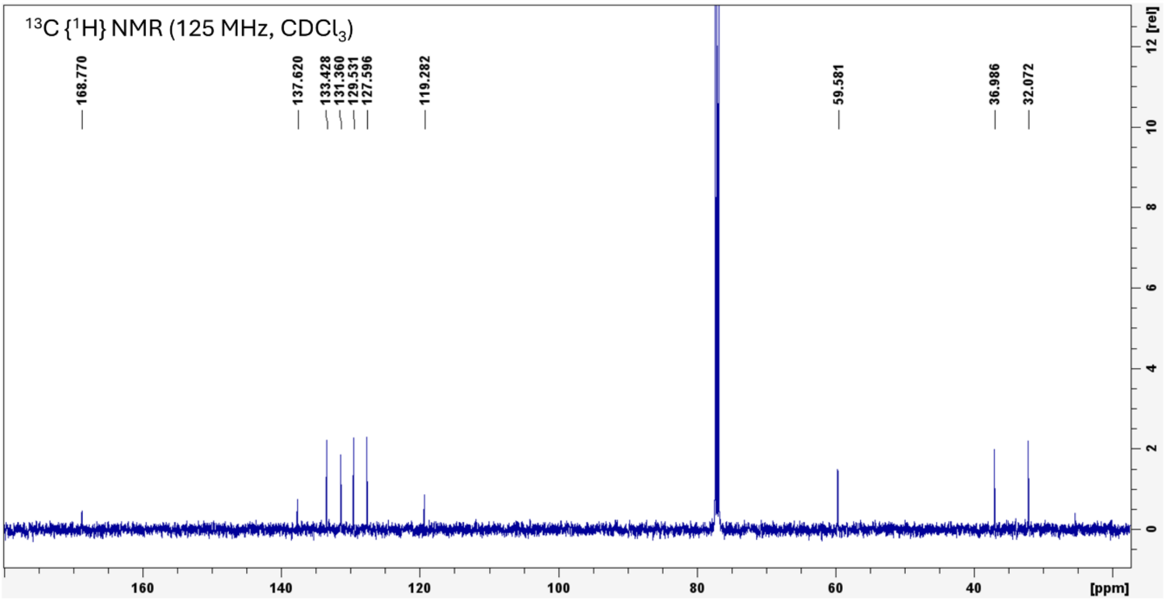
^{13}C {^{1}H} NMR (125 MHz, CDCl$_3$)
168.770
137.620
133.428 131.360 129.531 127.596
119.282
59.581
36.986
32.072
[ppm]
[rel]

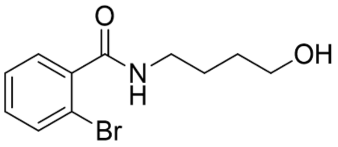
O
N
H
OH
Br

3d

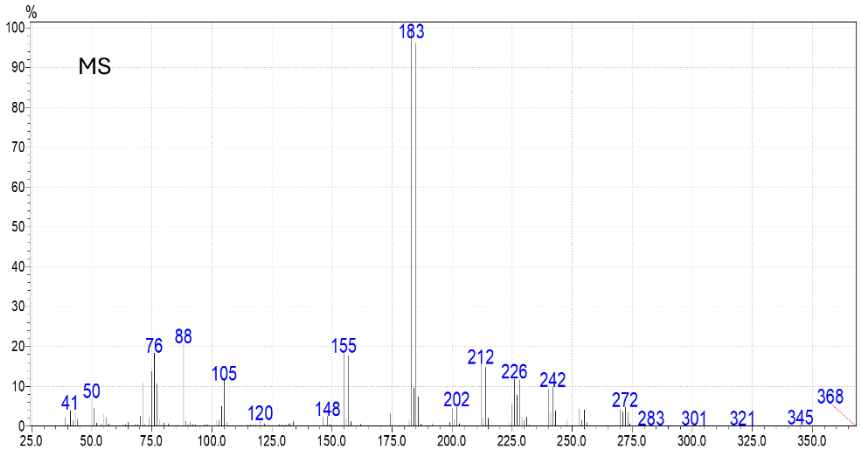
MS
%
183
76
88
155
212
226
242
105
50
41
120
148
202
272
283
301
321
345
368

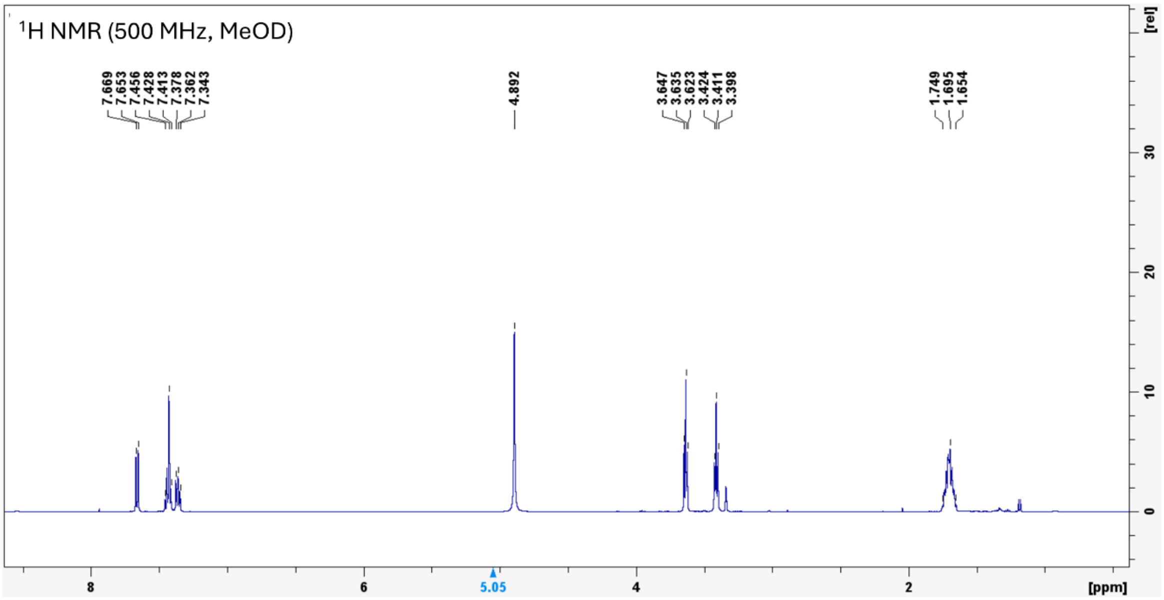
^{1}H NMR (500 MHz, MeOD)
7.669
7.653
7.456
7.428
7.413
7.378
7.362
7.343
4.892
3.647
3.635
3.623
3.424
3.411
3.398
1.749
1.695
1.654
5.05
[ppm]
[rel]

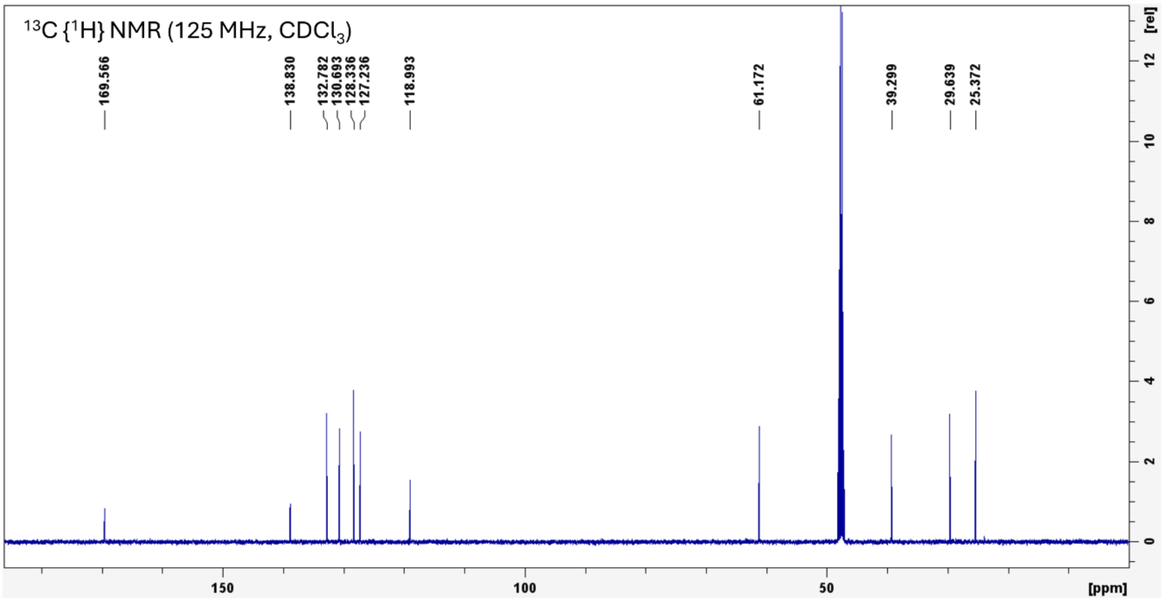
^{13}C {^{1}H} NMR (125 MHz, $CDCl_3$)
169.566
138.830
132.782
130.693
128.336
127.236
118.993
61.172
39.299
29.639
25.372
[ppm]
[rel]

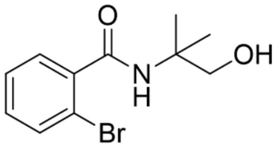

3e

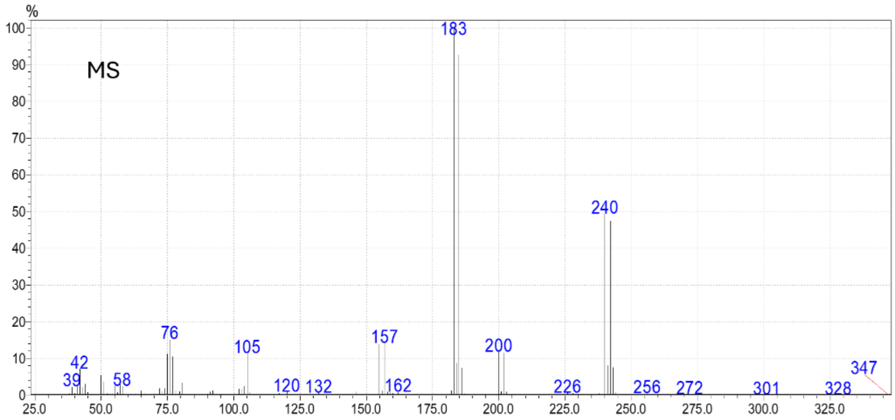
MS
%
183
240
76
105
157
200
42
39
58
120 132
162
226
256
272
301
328
347

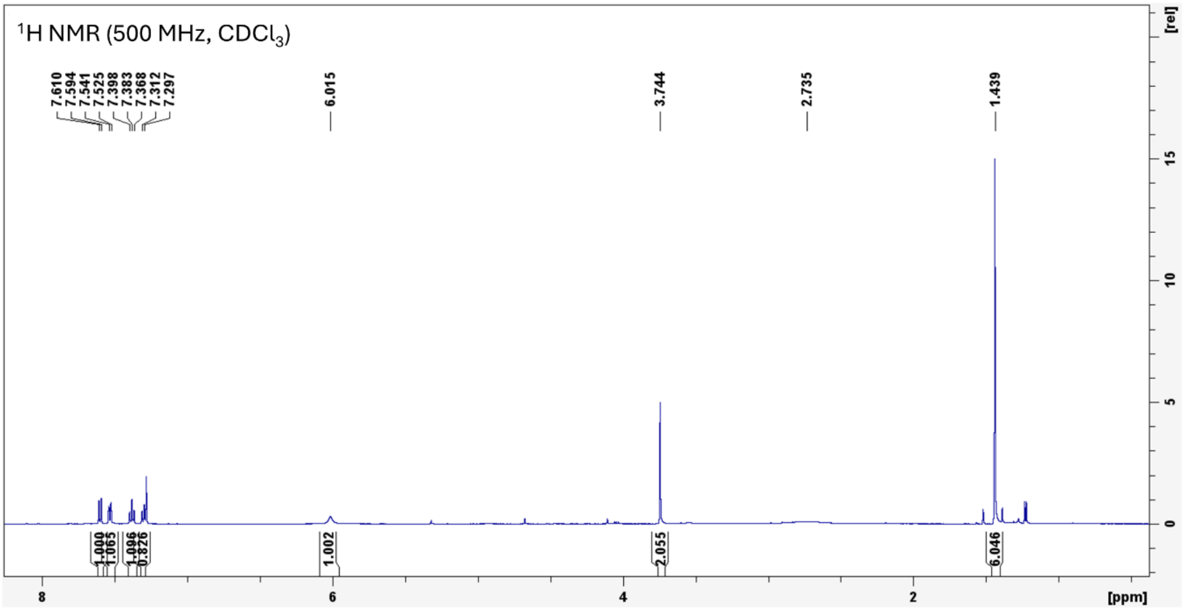
1H NMR (500 MHz, CDCl3)
7.610
7.594
7.541
7.525
7.398
7.383
7.368
7.312
7.297
6.015
3.744
2.735
1.439
1.000
1.065
1.096
0.826
1.002
2.055
6.046
[rel]
[ppm]

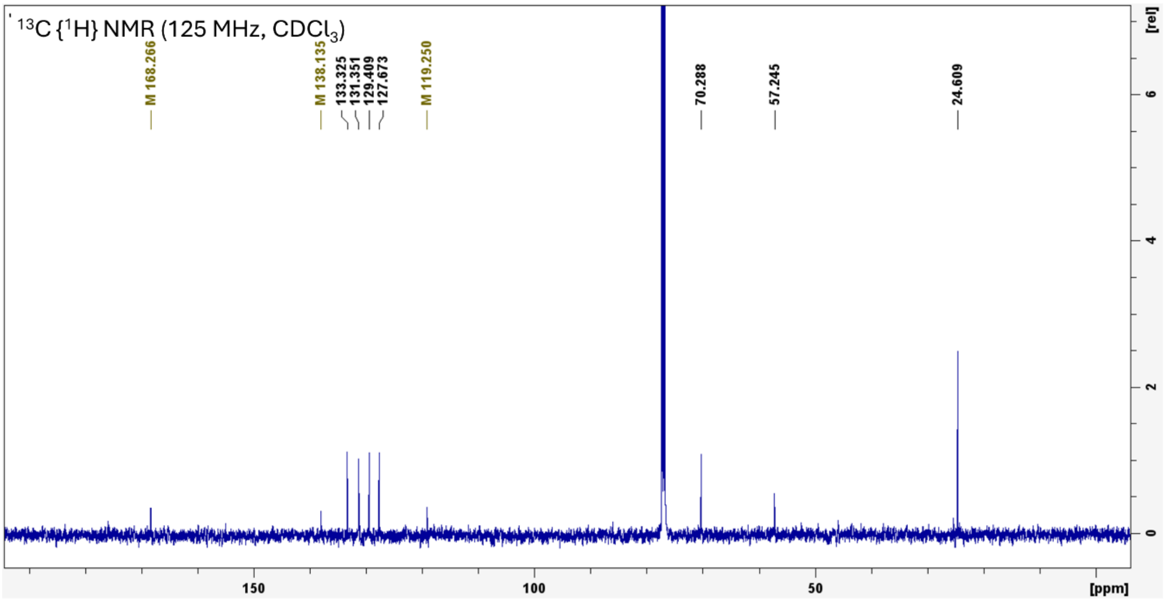
13C {1H} NMR (125 MHz, CDCl3)
M 168.266
M 138.135
133.325
131.351
129.409
127.673
M 119.250
70.288
57.245
24.609
[rel]
[ppm]

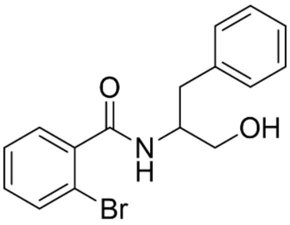
O
N
H
OH
Br

3f

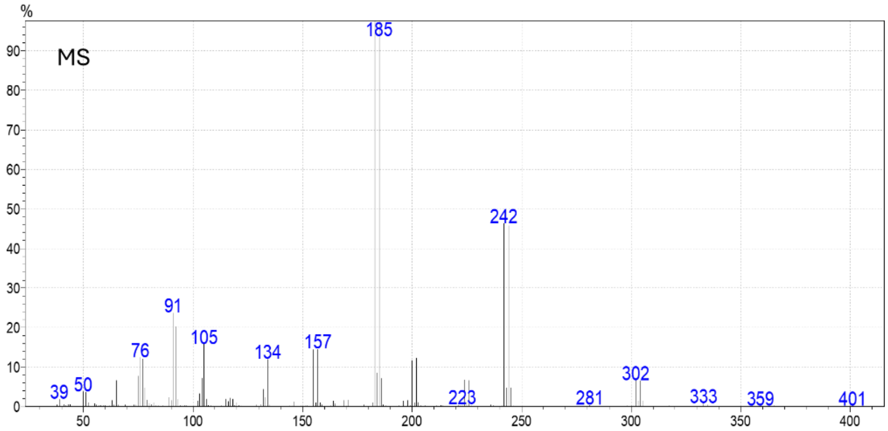
MS
%
185
242
91
105
76
134
157
39
50
223
281
302
333
359
401

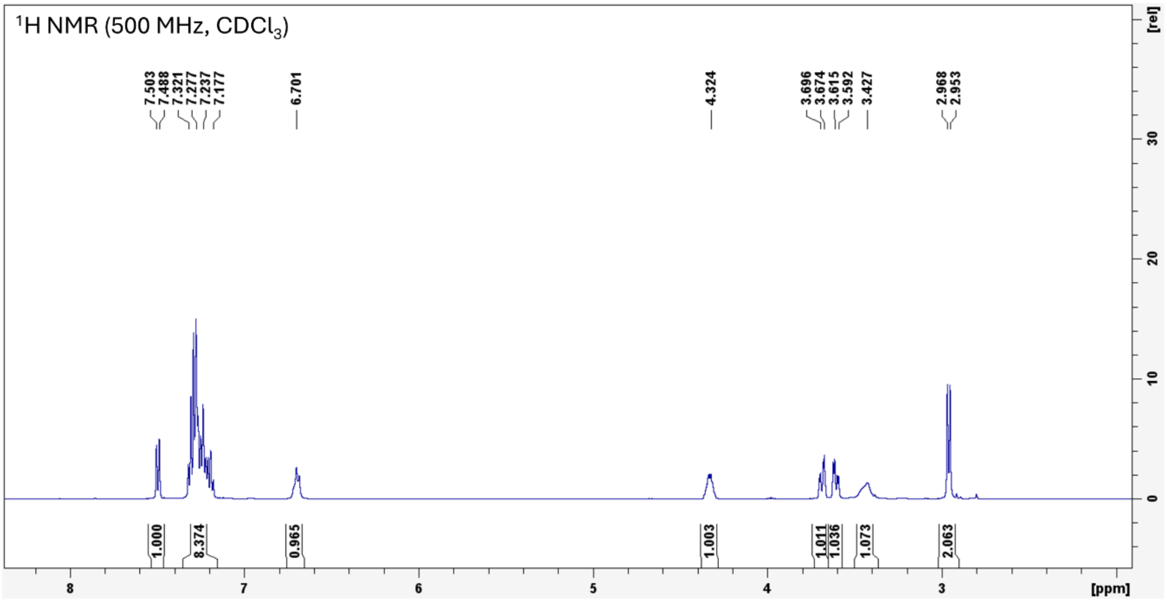
¹H NMR (500 MHz, CDCl₃)
7.503
7.488
7.321
7.277
7.237
7.177
6.701
4.324
3.696
3.674
3.615
3.592
3.427
2.968
2.953
1.000
8.374
0.965
1.003
1.011
1.036
1.073
2.063
[ppm]
[rel]

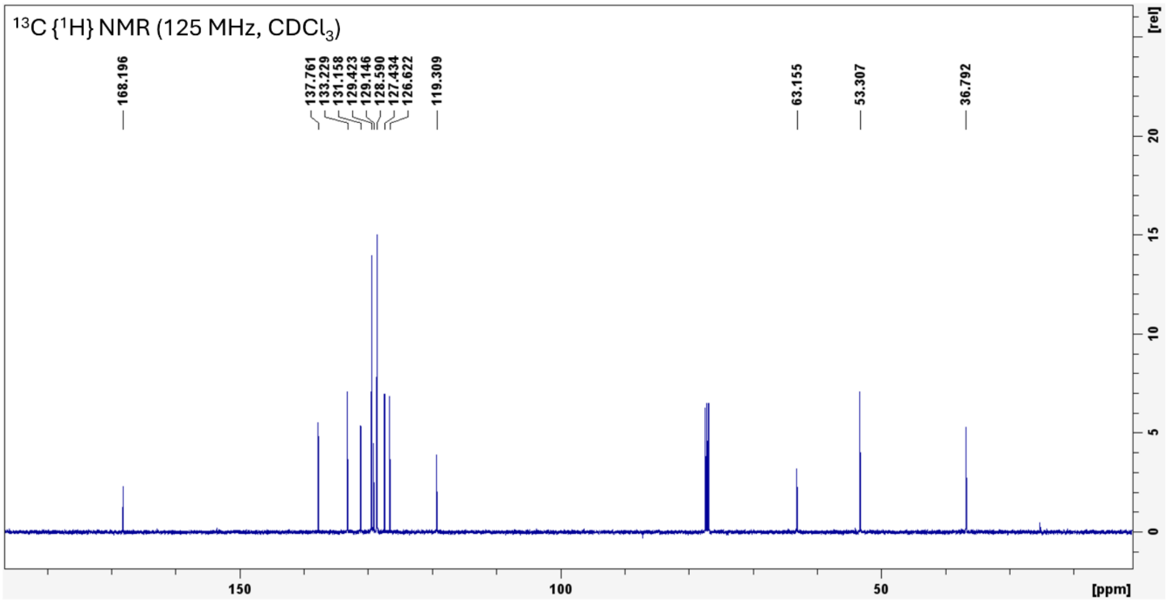
¹³C {¹H} NMR (125 MHz, CDCl₃)
168.196
137.761
133.229
131.158
129.423
129.146
128.590
127.434
126.622
119.309
63.155
53.307
36.792
[ppm]
[rel]

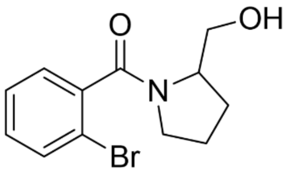
O
OH
N
Br

3g

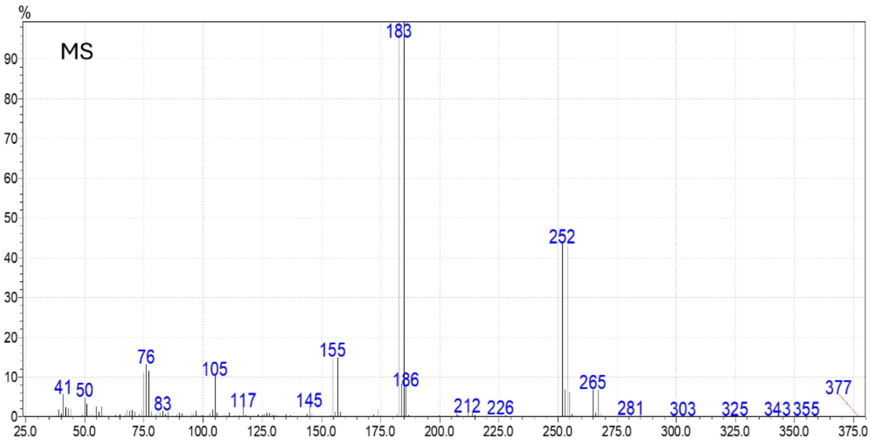
MS
%
183
252
155
76
105
186
265
41
50
83
117
145
212
226
281
303
325
343
355
377

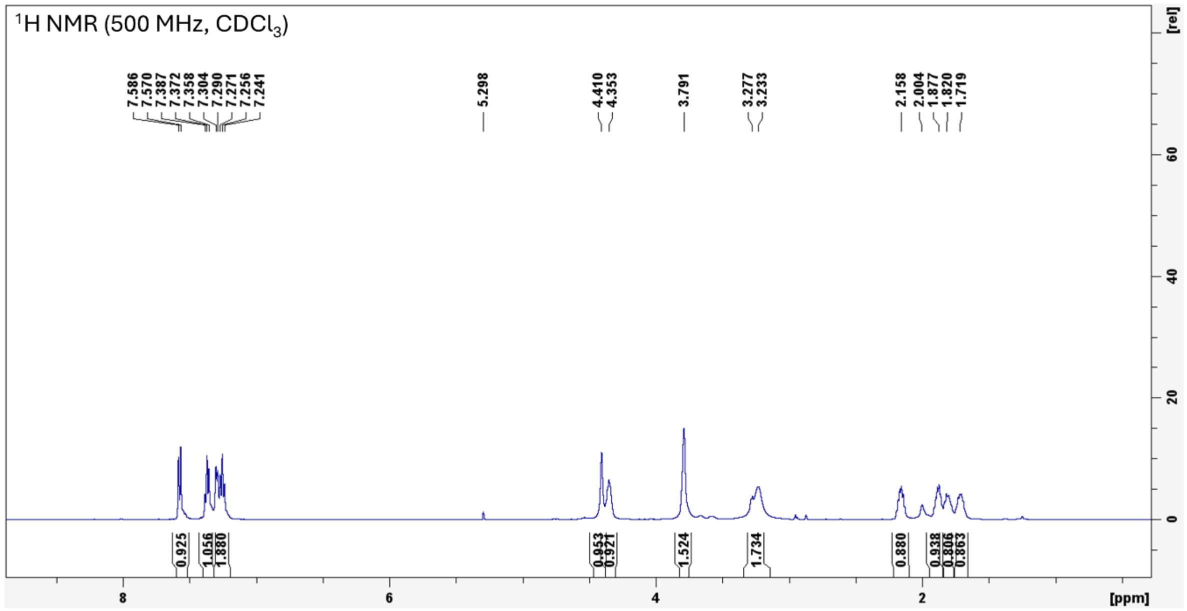
^{1}H NMR (500 MHz, CDCl$_3$)
7.586
7.570
7.387
7.372
7.358
7.304
7.290
7.271
7.256
7.241
5.298
4.410
4.353
3.791
3.277
3.233
2.158
2.004
1.877
1.820
1.719
0.925
1.056
1.880
0.953
0.921
1.524
1.734
0.880
0.938
0.806
0.863
[ppm]
[rel]

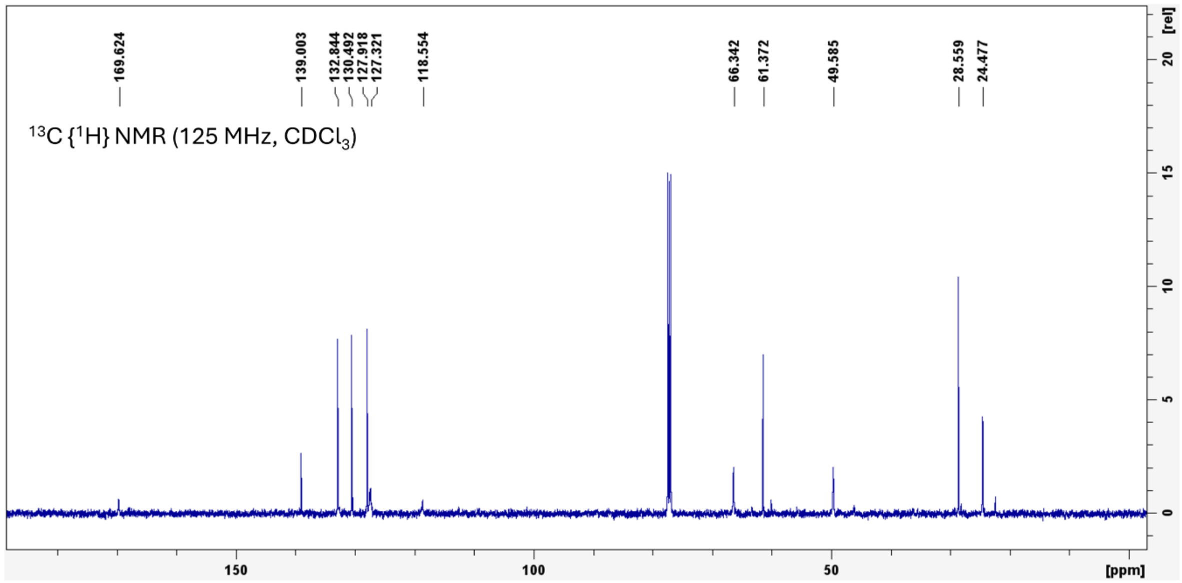
^{13}C {^{1}H} NMR (125 MHz, CDCl$_3$)
169.624
139.003
132.844
130.492
127.918
127.321
118.554
66.342
61.372
49.585
28.559
24.477
[ppm]
[rel]

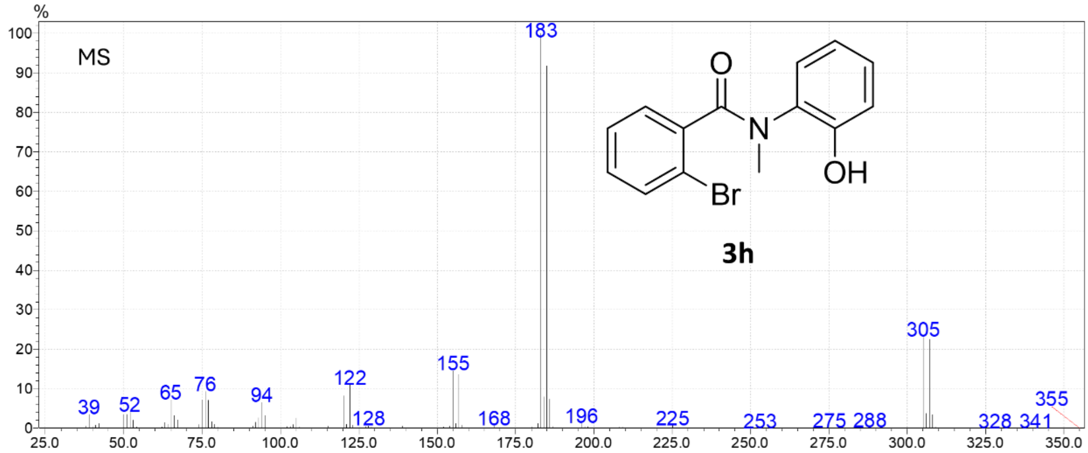
MS
%
183
305
155
122
76
65
94
52
39
128
168
196
225
253
275 288
328 341
355
O
N
OH
Br
3h

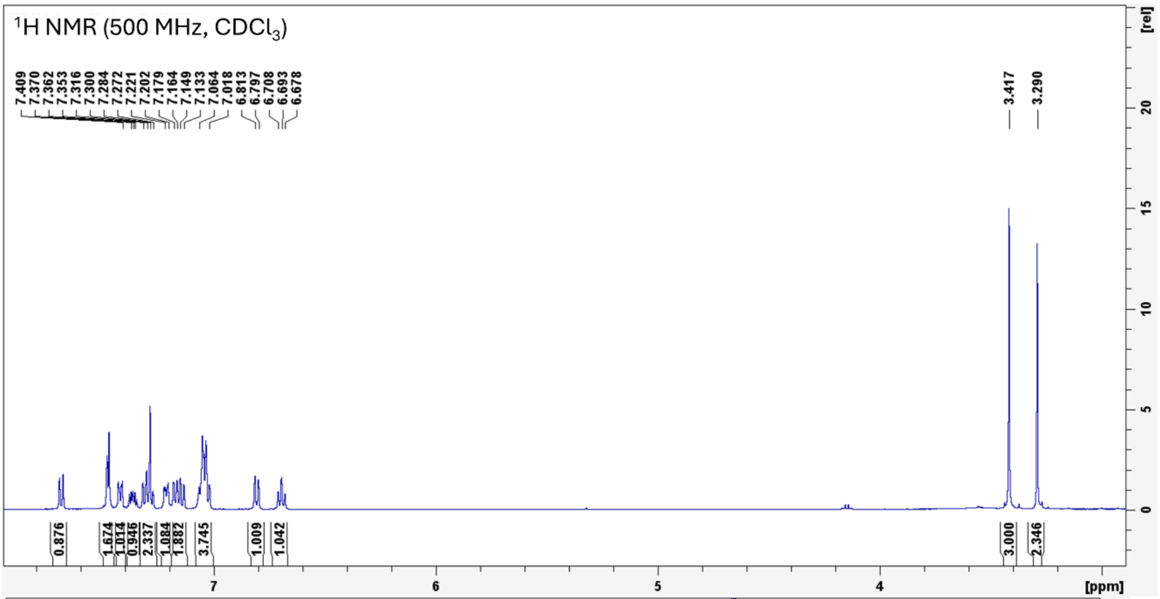
1H NMR (500 MHz, CDCl3)
7.409 7.370 7.362 7.353 7.316 7.300 7.284 7.272 7.221 7.202 7.179 7.164 7.149 7.133 7.064 7.018 6.813 6.797 6.708 6.693 6.678
3.417
3.290
0.876 1.674 1.014 0.946 2.337 1.084 1.882 3.745 1.009 1.042
3.000 2.346
[ppm]
[rel]

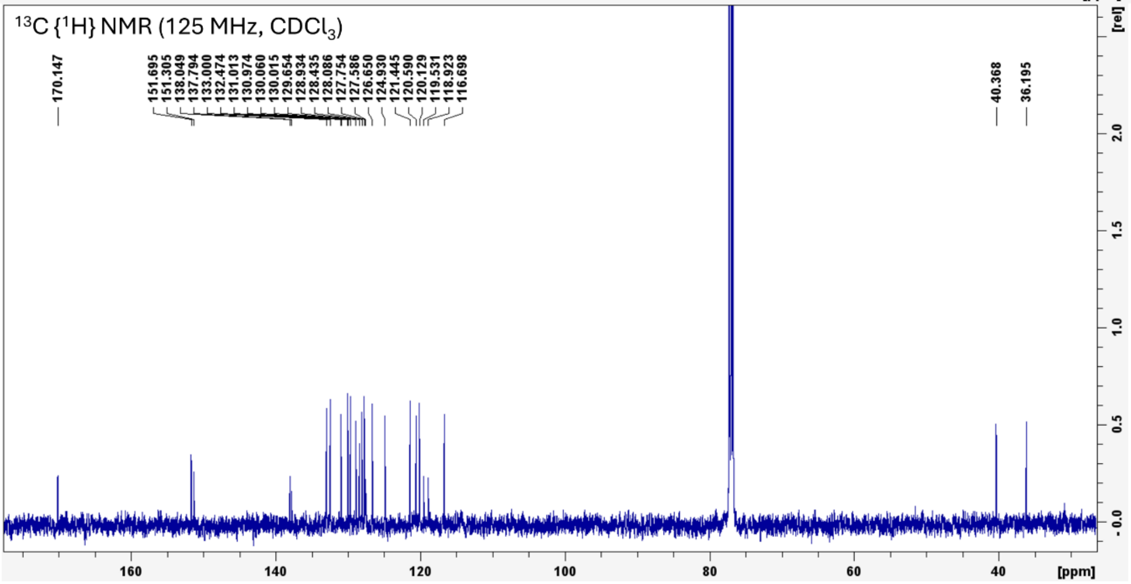
13C {1H} NMR (125 MHz, CDCl3)
170.147
151.695 151.305 138.049 137.794 133.000 132.474 131.013 130.974 130.060 130.015 129.654 128.934 128.435 128.086 127.754 127.586 126.650 124.930 121.445 120.590 120.129 119.531 118.923 116.698
40.368
36.195
[ppm]
[rel]

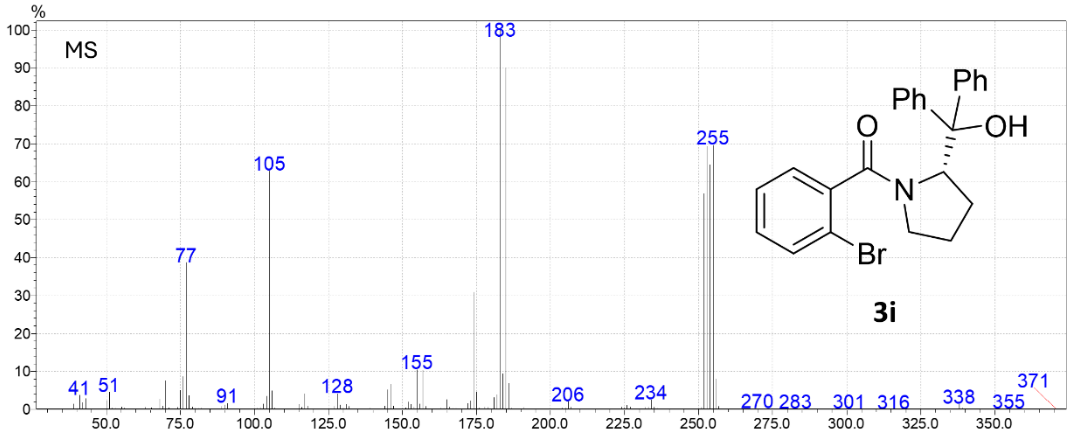
MS
%
183
255
105
77
155
41 51
91
128
206
234
270 283
301 316
338
355
371
Ph
Ph
OH
O
N
Br
3i

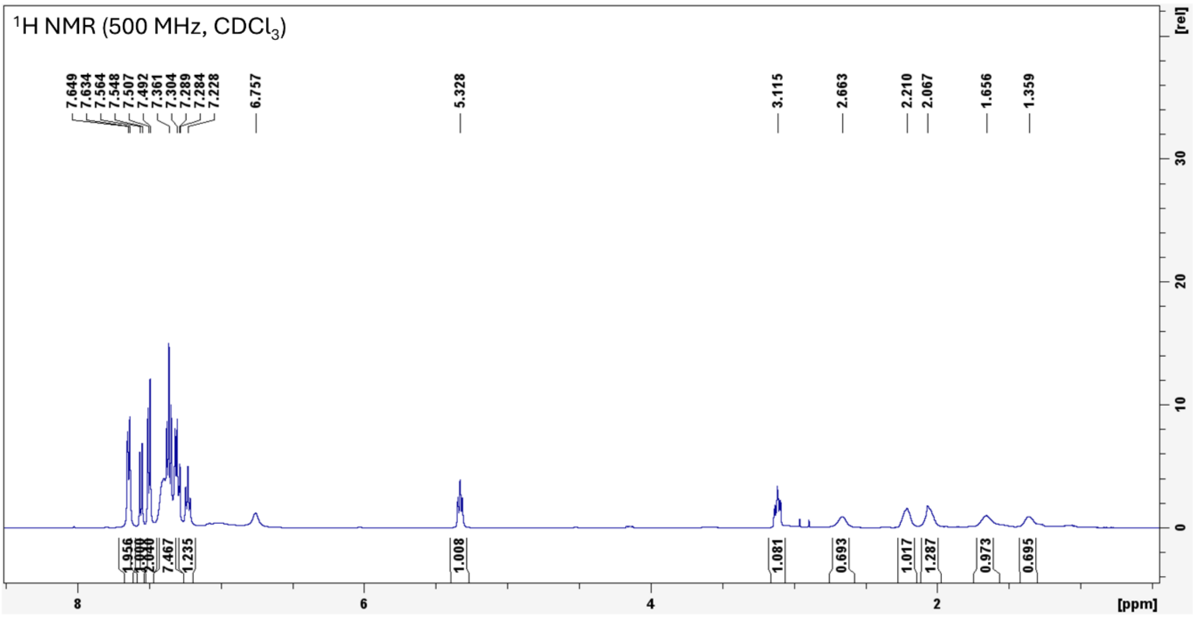
^{1}H NMR (500 MHz, CDCl$_3$)
7.649 7.634 7.564 7.548 7.507 7.492 7.361 7.304 7.289 7.284 7.228
6.757
5.328
3.115
2.663
2.210
2.067
1.656
1.359
1.956 7.467 1.235
1.008
1.081
0.693
1.017 1.287
0.973
0.695
[ppm]
[rel]

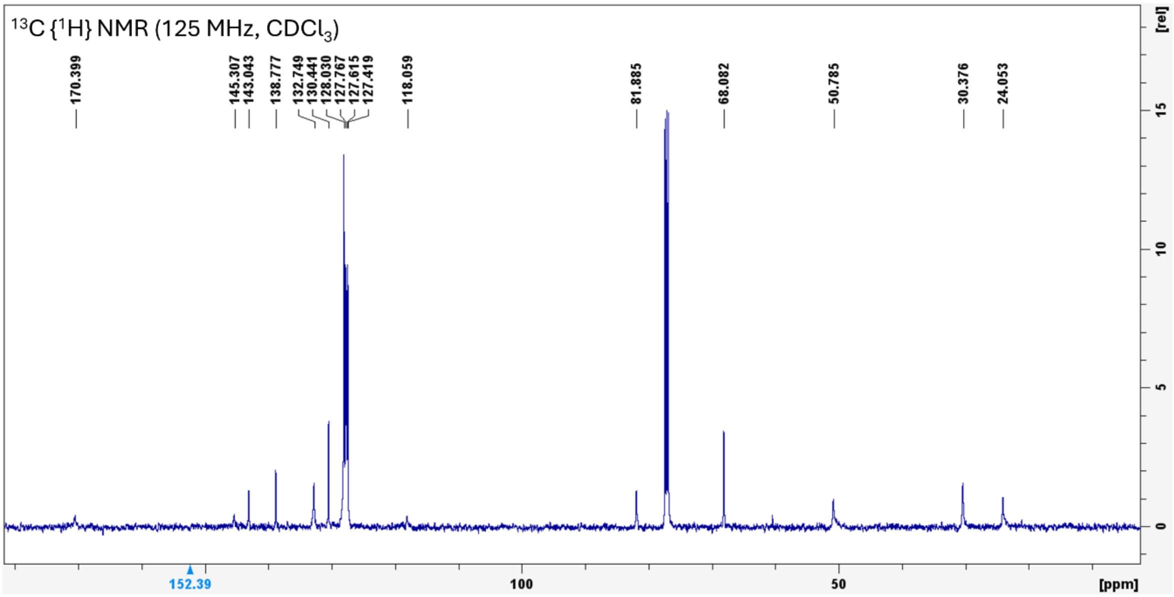
^{13}C {^{1}H} NMR (125 MHz, CDCl$_3$)
170.399
145.307 143.043 138.777 132.749 130.441 128.030 127.767 127.615 127.419
118.059
81.885
68.082
50.785
30.376
24.053
152.39
[ppm]
[rel]

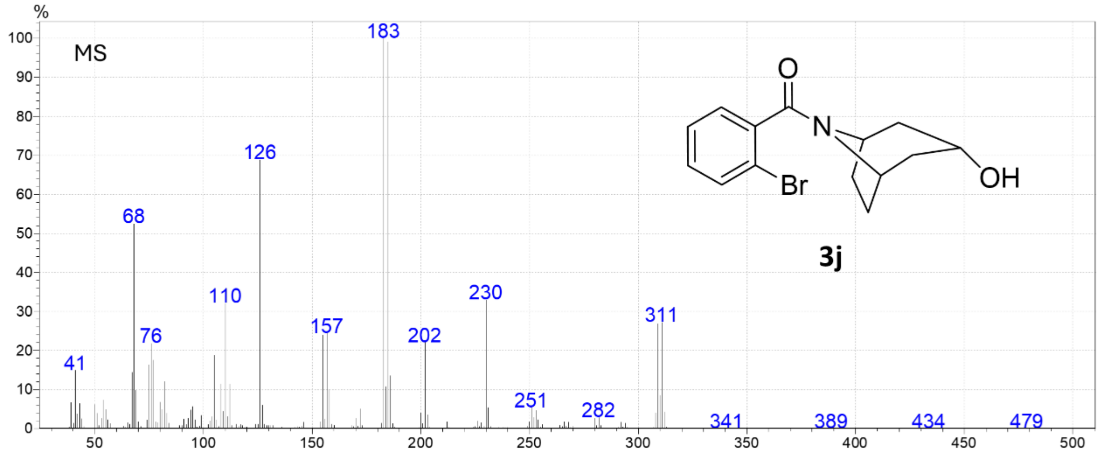

MS
%
183
126
68
110
230
311
157
202
76
41
251
282
341
389
434
479
O
N
Br
OH
3j

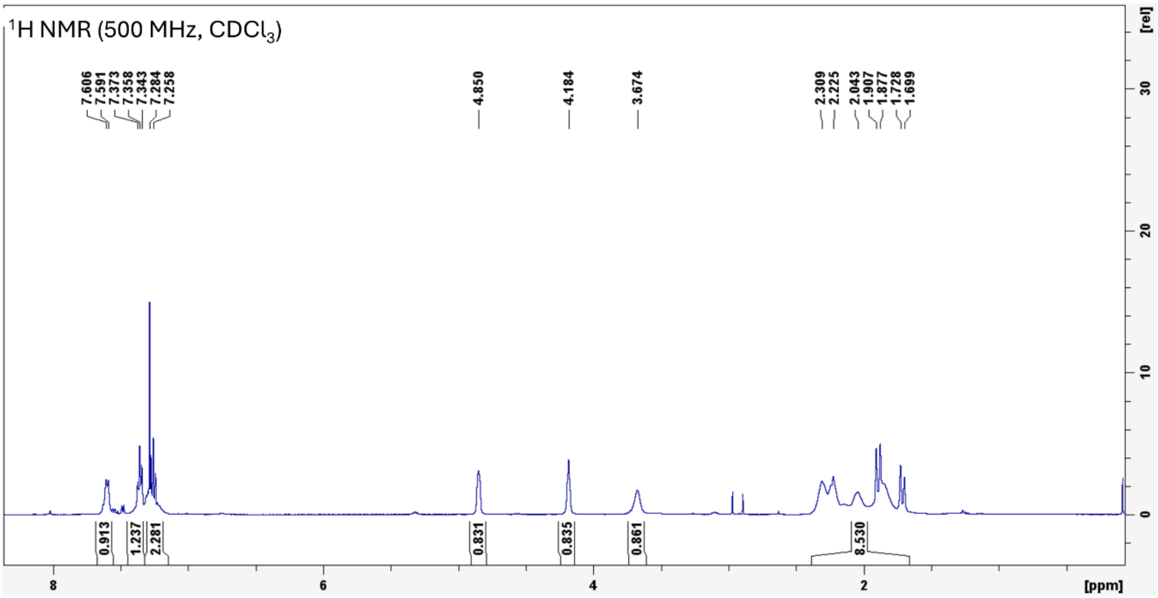

^{1}H NMR (500 MHz, $CDCl_3$)
7.606
7.591
7.373
7.358
7.343
7.284
7.258
4.850
4.184
3.674
2.309
2.225
2.043
1.907
1.877
1.728
1.699
0.913
1.237
2.281
0.831
0.835
0.861
8.530
[ppm]
[rel]

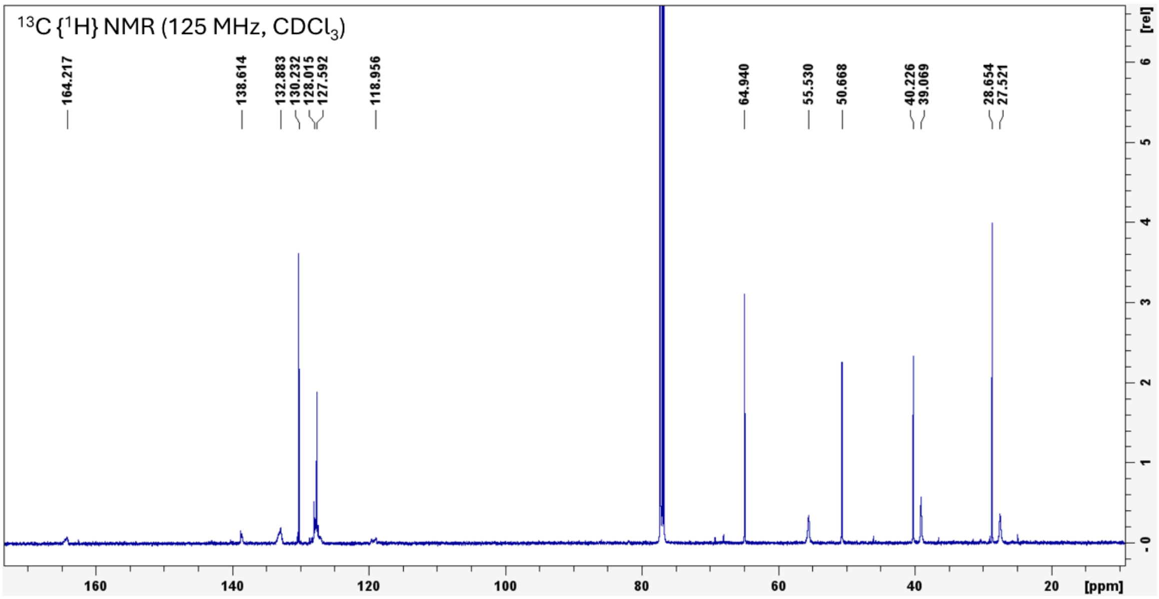

^{13}C {^{1}H} NMR (125 MHz, $CDCl_3$)
164.217
138.614
132.883
130.232
128.015
127.592
118.956
64.940
55.530
50.668
40.226
39.069
28.654
27.521
[ppm]
[rel]

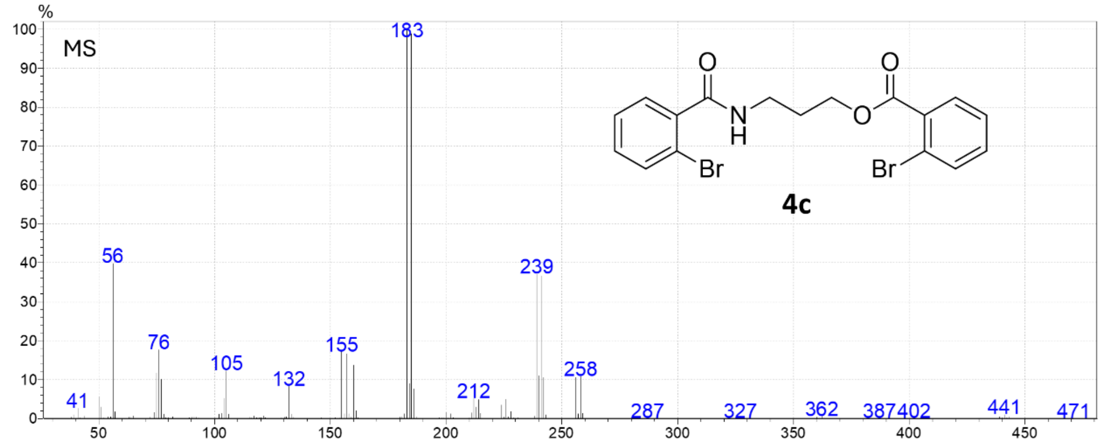
MS
4c
183
56
239
76
155
258
105
132
212
41
287
327
362
387402
441
471

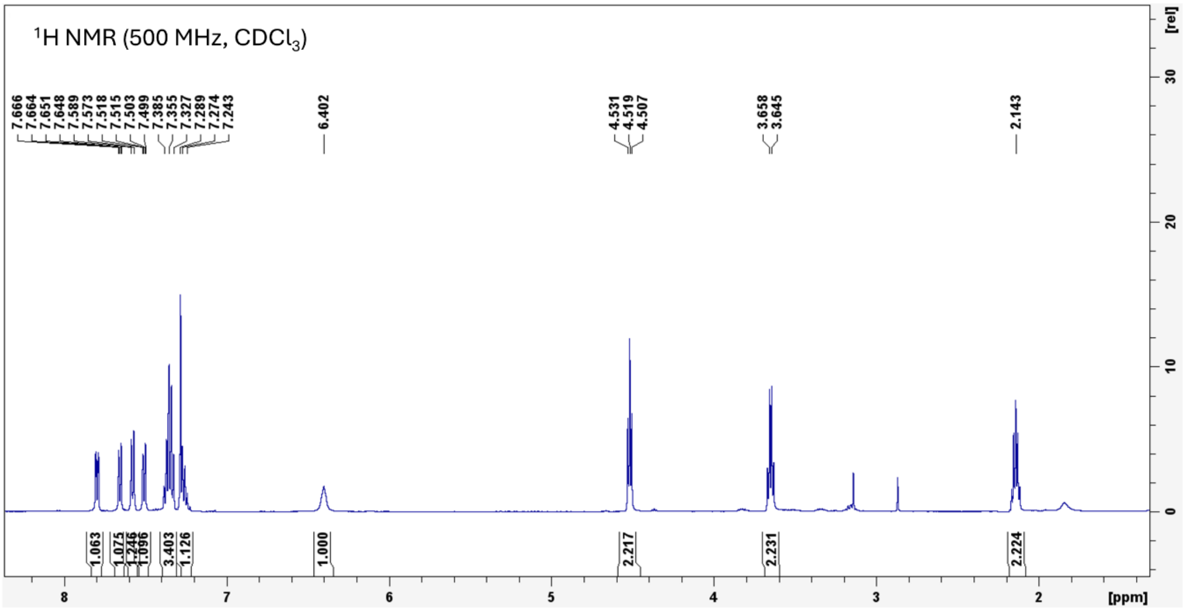
1H NMR (500 MHz, CDCl3)
7.666 7.664 7.651 7.648 7.589 7.573 7.518 7.515 7.503 7.499 7.385 7.355 7.327 7.289 7.274 7.243
6.402
4.531 4.519 4.507
3.658 3.645
2.143
1.063 1.075 1.246 1.096 3.403 1.126 1.000 2.217 2.231 2.224
[ppm]
[rel]

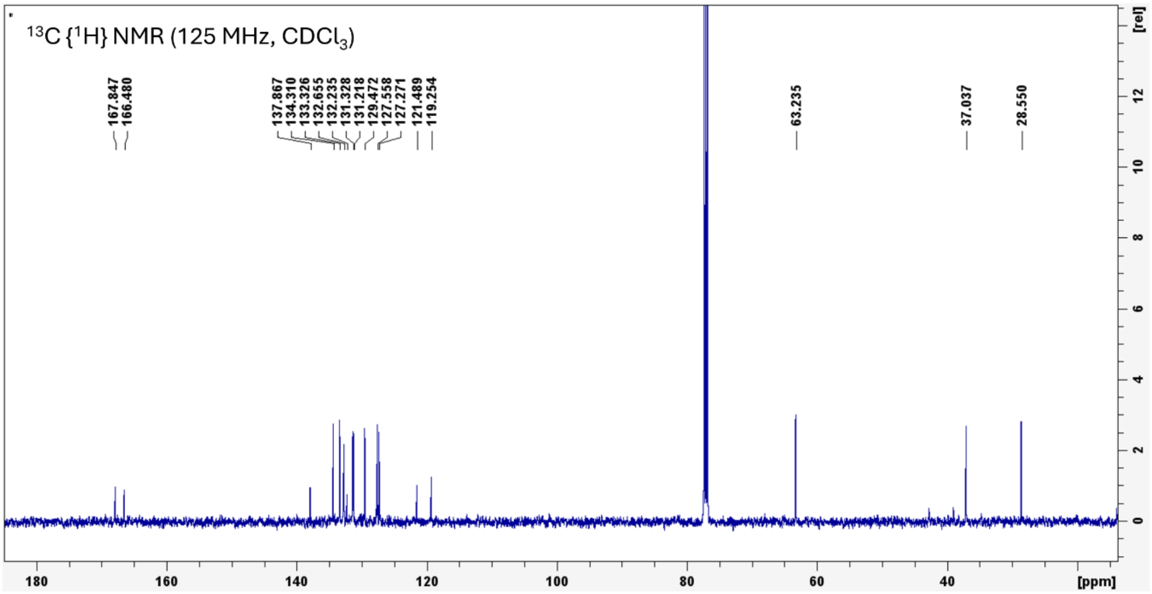
13C {1H} NMR (125 MHz, CDCl3)
167.847 166.480
137.867 134.310 133.326 132.655 132.235 131.328 131.218 129.472 127.558 127.271 121.489 119.254
63.235
37.037
28.550
[ppm]
[rel]

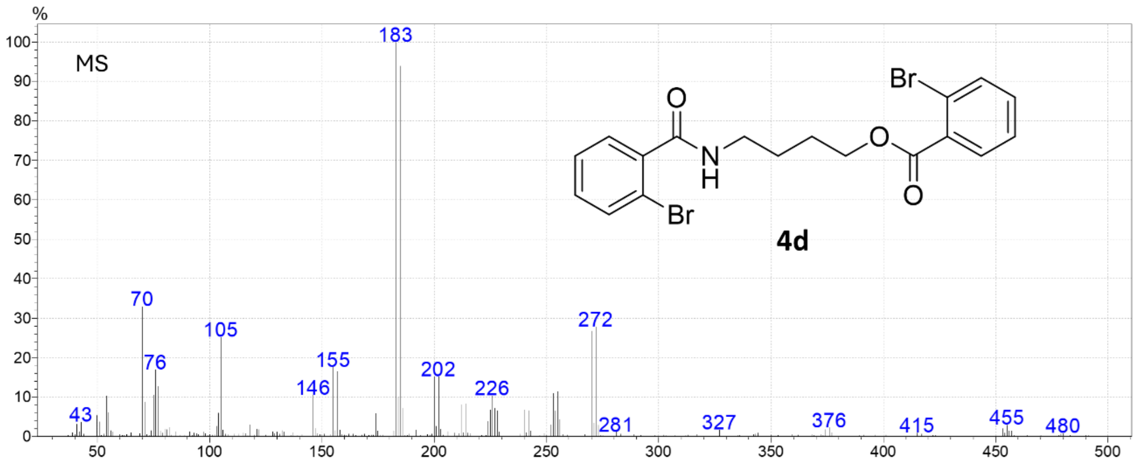
MS
4d
Br
O
N
H
Br
O
O
183
70
105
272
76
155
202
226
146
43
281
327
376
415
455
480

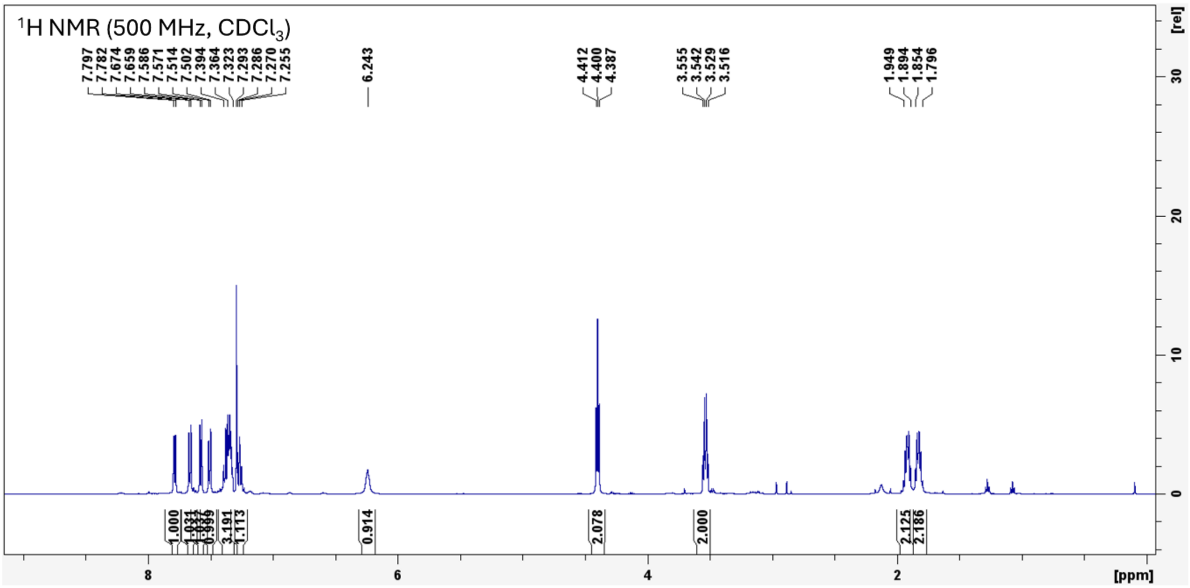
^{1}H NMR (500 MHz, CDCl$_3$)
7.797
7.782
7.674
7.659
7.586
7.571
7.514
7.502
7.394
7.364
7.323
7.293
7.286
7.270
7.255
6.243
4.412
4.400
4.387
3.555
3.542
3.529
3.516
1.949
1.894
1.854
1.796
1.000
1.031
3.191
1.113
0.914
2.078
2.000
2.125
2.186
[ppm]
[rel]

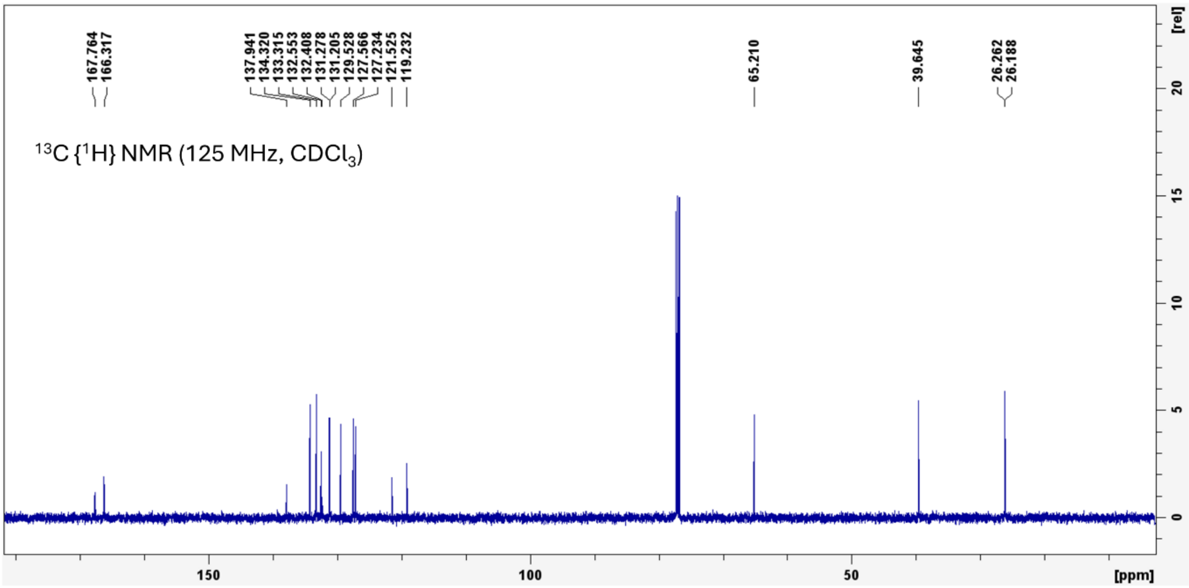
^{13}C {^{1}H} NMR (125 MHz, CDCl$_3$)
167.764
166.317
137.941
134.320
133.315
132.553
132.408
131.278
131.205
129.528
127.566
127.234
121.525
119.232
65.210
39.645
26.262
26.188
[ppm]
[rel]

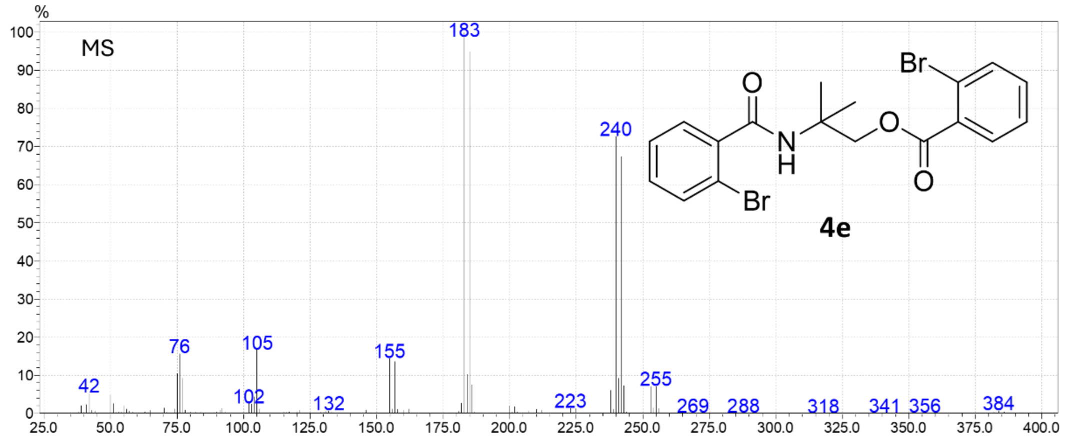
MS
183
240
76
105
155
255
42
102
132
223
269
288
318
341
356
384
Br
O
N
H
O
O
Br
4e

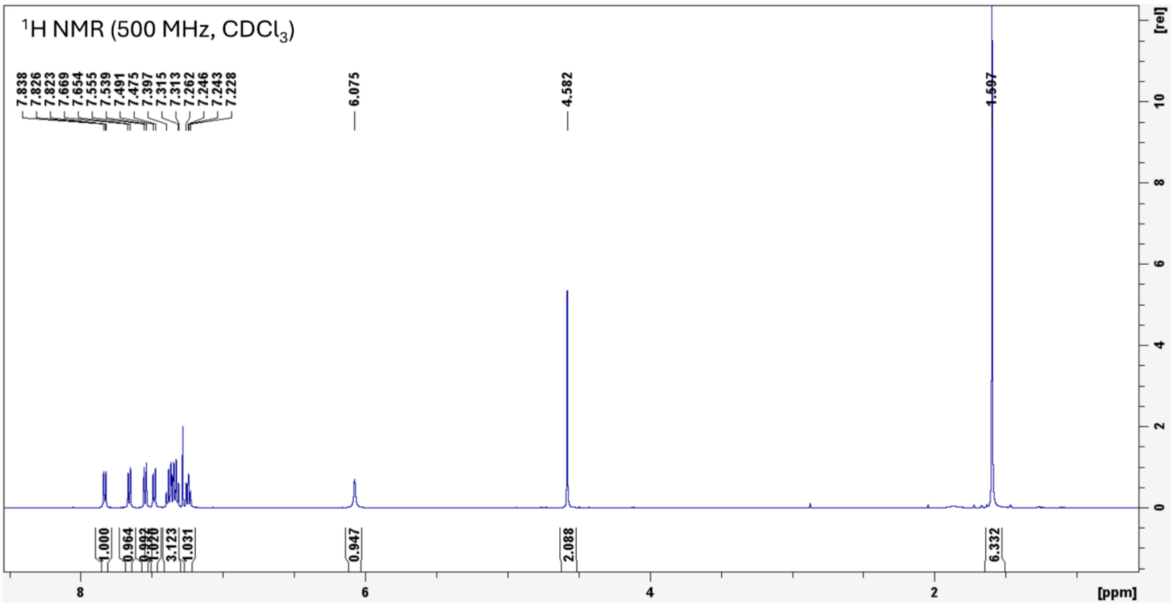
^{1}H NMR (500 MHz, CDCl$_3$)
7.838
7.826
7.823
7.669
7.654
7.555
7.539
7.491
7.475
7.397
7.315
7.313
7.262
7.246
7.243
7.228
6.075
4.582
1.597
1.000
0.964
3.123
1.031
0.947
2.088
6.332
[ppm]
[rel]

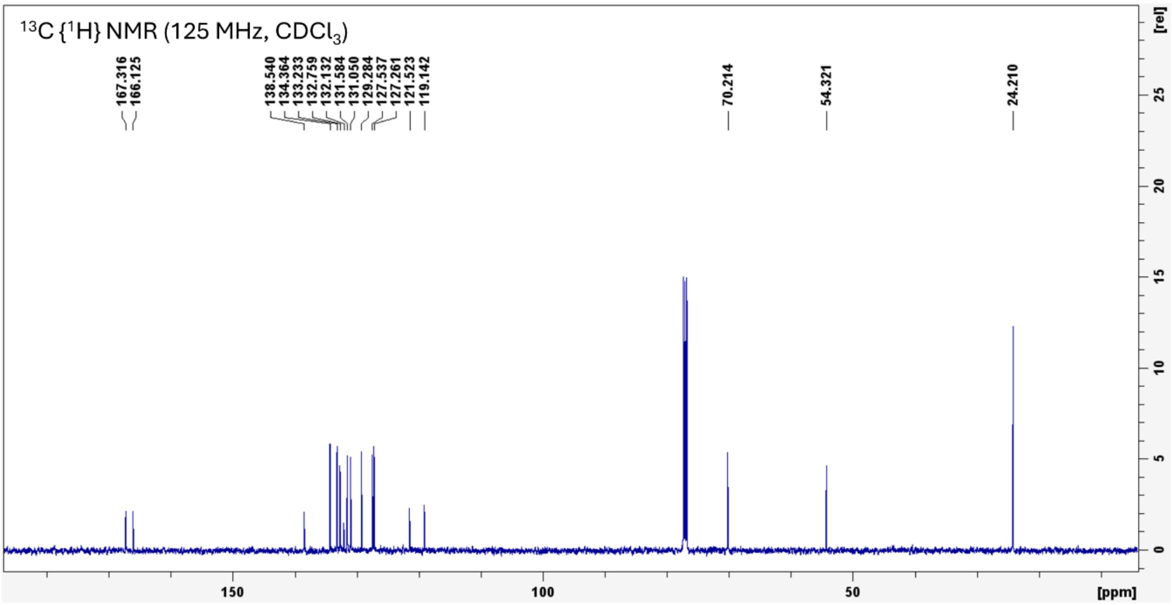
^{13}C {^{1}H} NMR (125 MHz, CDCl$_3$)
167.316
166.125
138.540
134.364
133.233
132.759
132.132
131.584
131.050
129.284
127.537
127.261
121.523
119.142
70.214
54.321
24.210
[ppm]
[rel]

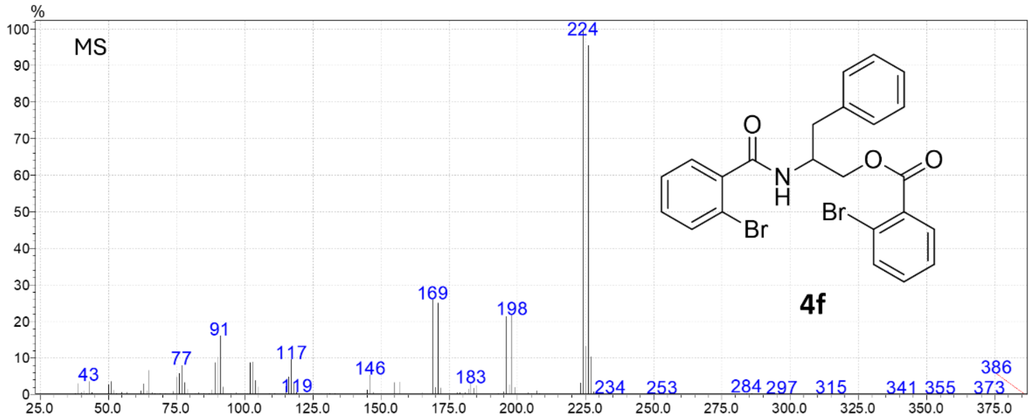
MS
224
169
198
91
117
77
43
146
183
119
234
253
284
297
315
341
355
373
386
O
N
H
O
O
Br
Br
4f

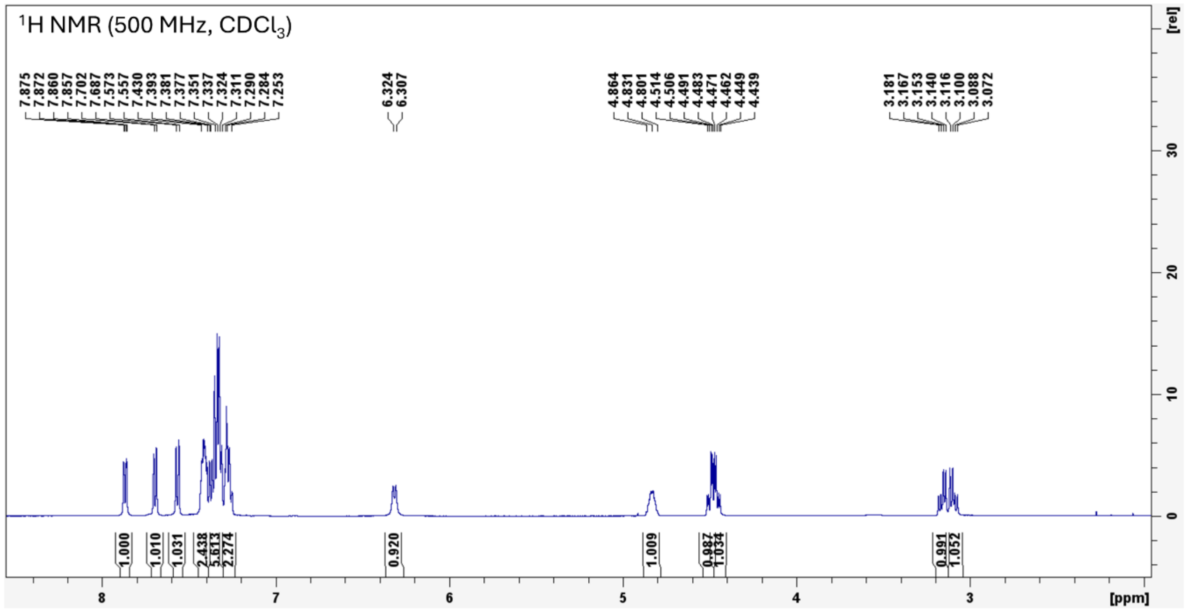
^{1}H NMR (500 MHz, CDCl$_3$)
7.875 7.872 7.860 7.857 7.702 7.687 7.573 7.557 7.430 7.393 7.381 7.377 7.351 7.337 7.324 7.311 7.290 7.284 7.253
6.324 6.307
4.864 4.831 4.801 4.514 4.506 4.491 4.483 4.471 4.462 4.449 4.439
3.181 3.167 3.153 3.140 3.116 3.100 3.088 3.072
1.000 1.010 1.031 2.438 5.613 2.274 0.920 1.009 0.987 1.034 0.991 1.052
[ppm]
[rel]

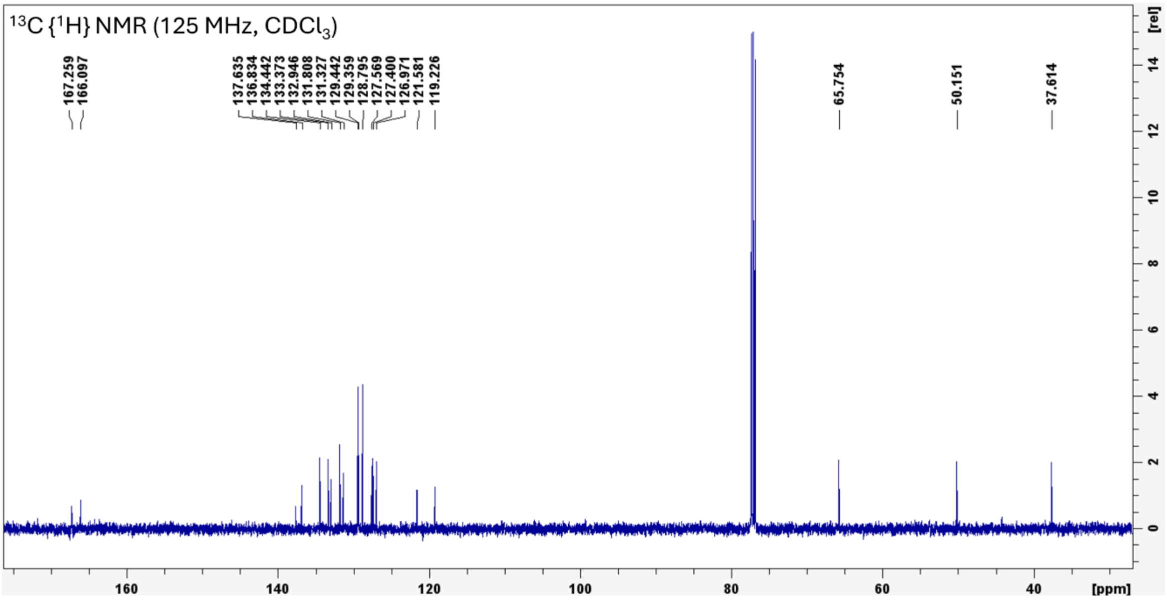
^{13}C {^{1}H} NMR (125 MHz, CDCl$_3$)
167.259 166.097
137.635 136.834 134.442 133.373 132.946 131.808 131.327 129.442 129.359 128.795 127.569 127.400 126.971 121.581 119.226
65.754
50.151
37.614
[ppm]
[rel]

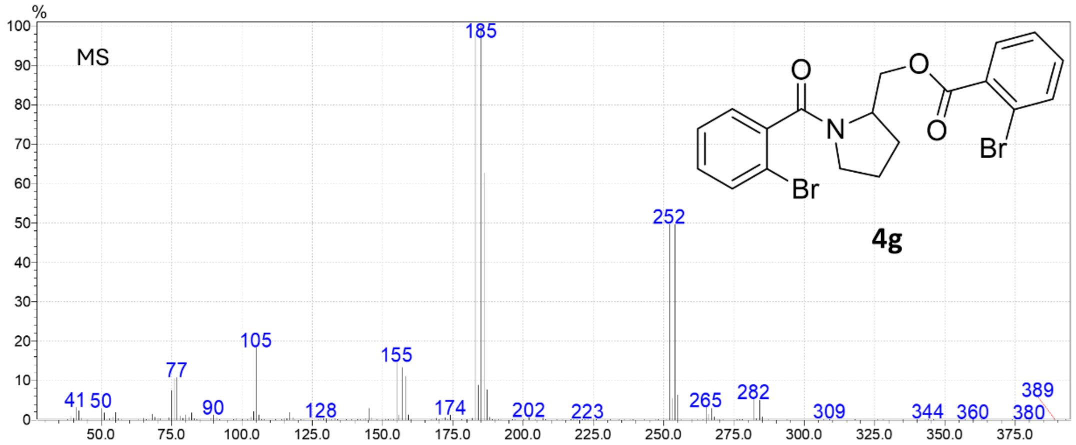
MS
4g
185
252
105
155
77
41
50
90
128
174
202
223
265
282
309
344
360
380
389

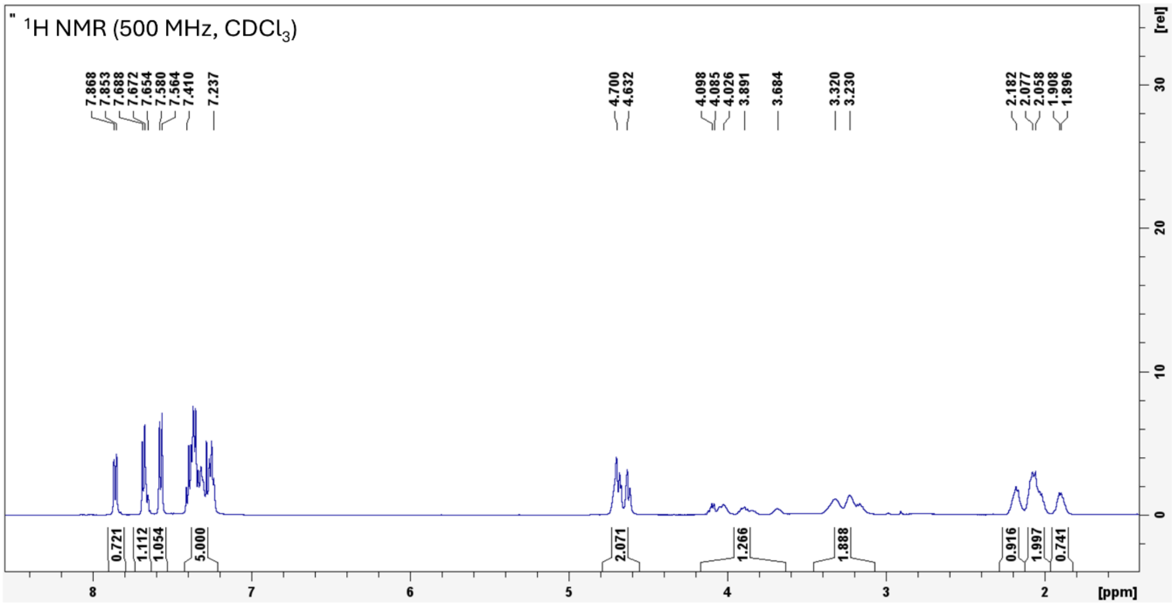
^{1}H NMR (500 MHz, $CDCl_3$)
7.868 7.853 7.688 7.672 7.654 7.580 7.564 7.410 7.237
4.700 4.632
4.098 4.085 4.026 3.891 3.684
3.320 3.230
2.182 2.077 2.058 1.908 1.896
0.721 1.112 1.054 5.000 2.071 1.266 1.888 0.916 1.997 0.741
[ppm]
[rel]

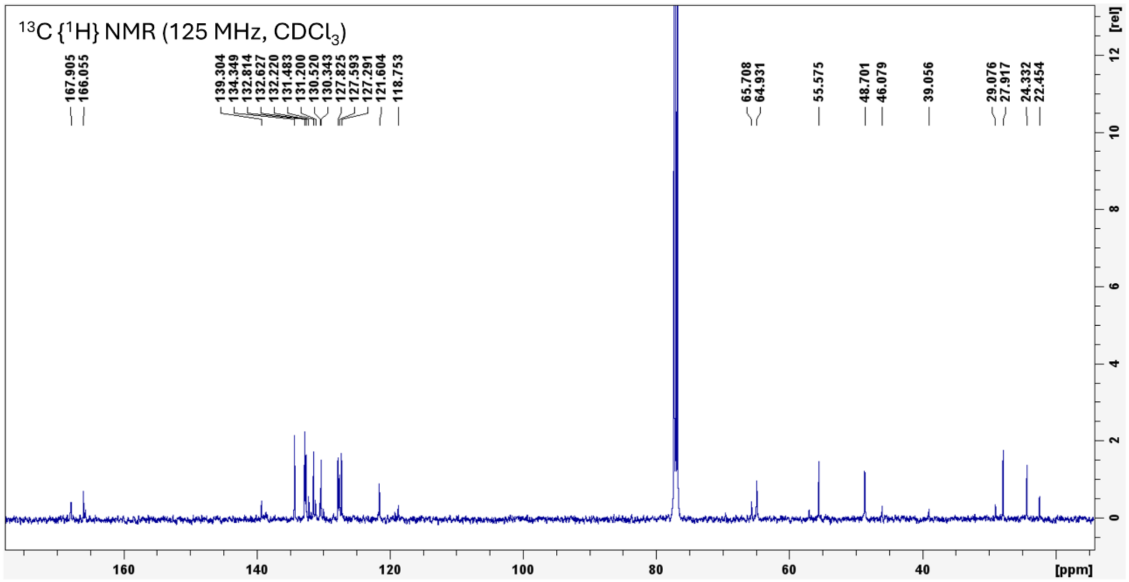
^{13}C {^{1}H} NMR (125 MHz, $CDCl_3$)
167.905 166.055
139.304 134.349 132.814 132.627 132.220 131.483 131.200 130.520 130.343 127.825 127.593 127.291 121.604 118.753
65.708 64.931
55.575
48.701 46.079
39.056
29.076 27.917 24.332 22.454
[ppm]
[rel]

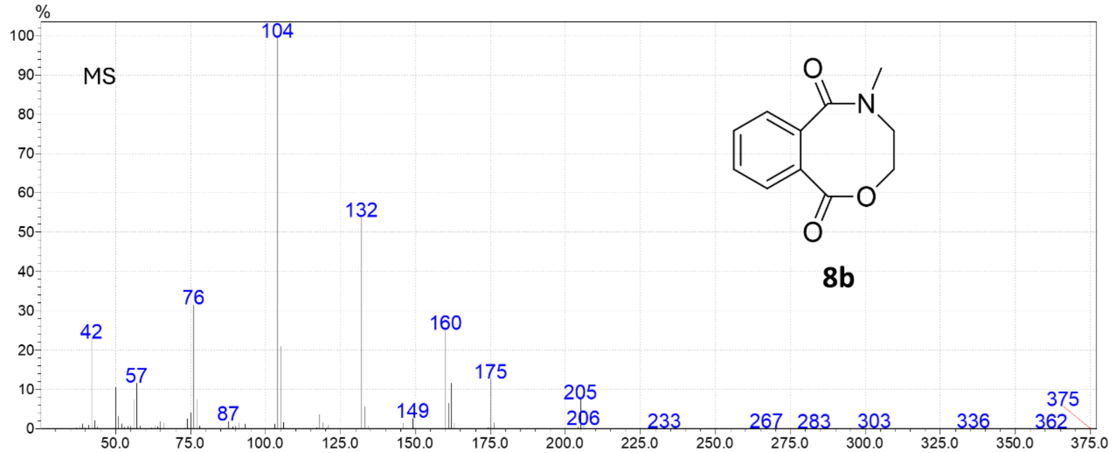

MS
%
104
132
76
42
160
57
175
205
206
87
149
233
267
283
303
336
375
362
O
N
O
O
8b

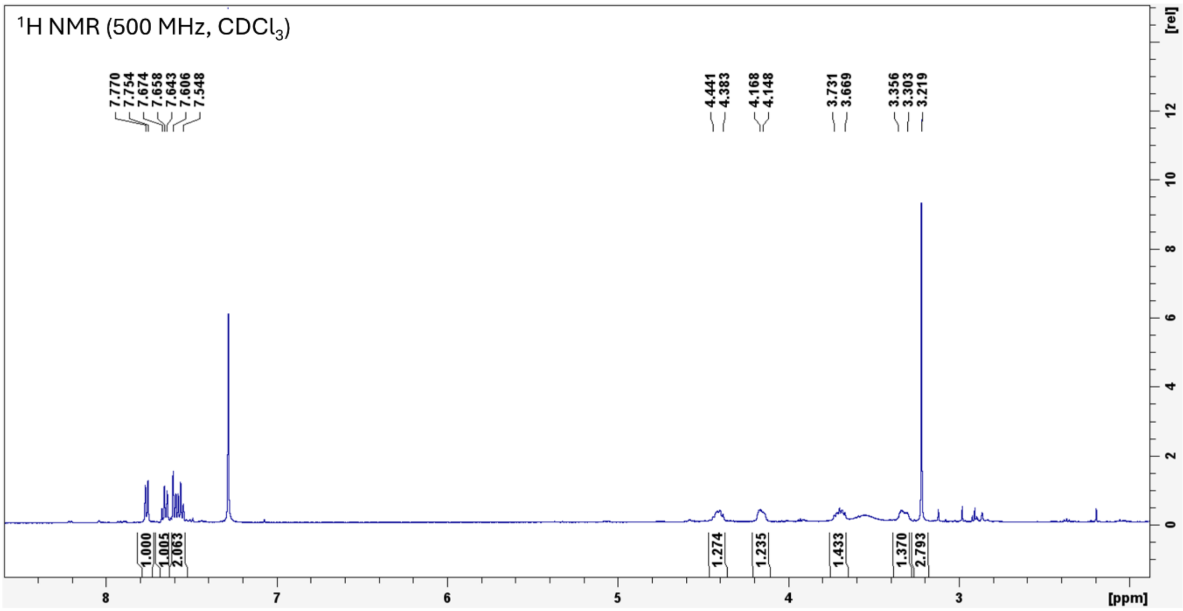

1H NMR (500 MHz, CDCl3)
7.770
7.754
7.674
7.658
7.643
7.606
7.548
4.441
4.383
4.168
4.148
3.731
3.669
3.356
3.303
3.219
1.000
1.005
2.063
1.274
1.235
1.433
1.370
2.793
[ppm]
[rel]

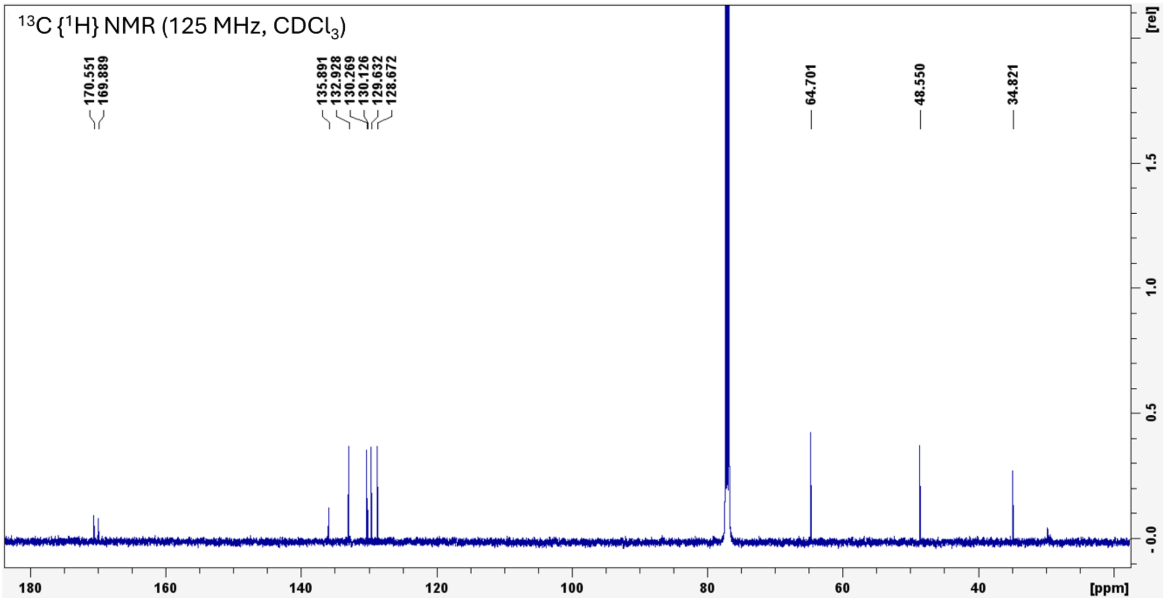

13C {1H} NMR (125 MHz, CDCl3)
170.551
169.889
135.891
132.928
130.269
130.126
129.632
128.672
64.701
48.550
34.821
[ppm]
[rel]

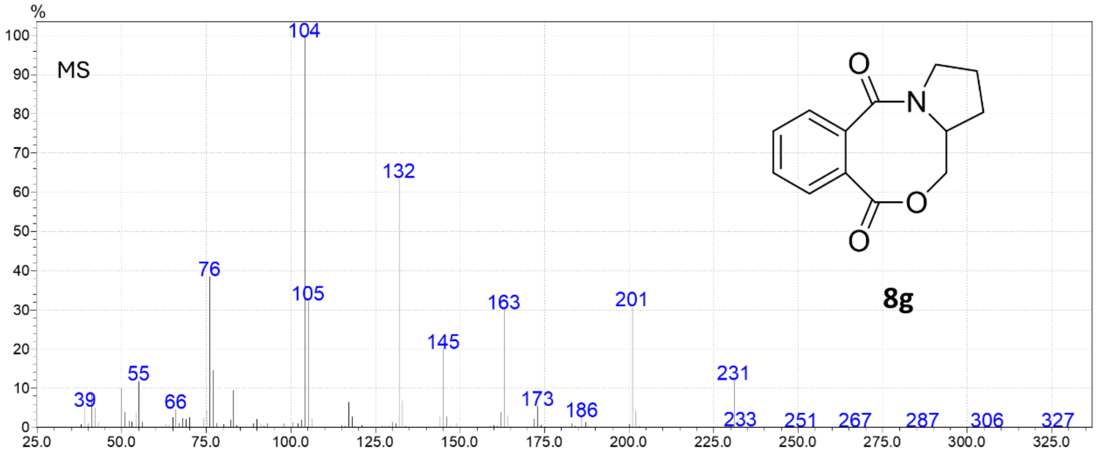
MS
%
104
132
76
105
163
201
145
55
231
39
66
173
186
233
251
267
287
306
327
8g

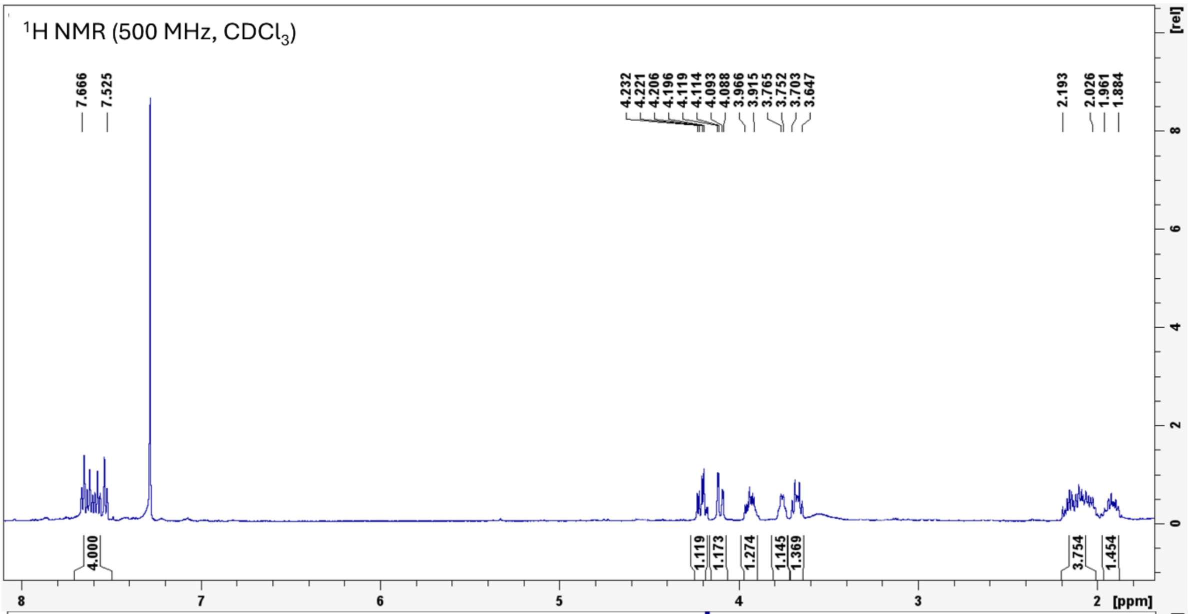
^{1}H NMR (500 MHz, CDCl$_3$)
7.666
7.525
4.232
4.221
4.206
4.196
4.119
4.114
4.093
4.088
3.966
3.915
3.765
3.752
3.703
3.647
2.193
2.026
1.961
1.884
4.000
1.119
1.173
1.274
1.145
1.369
3.754
1.454
[ppm]
[rel]

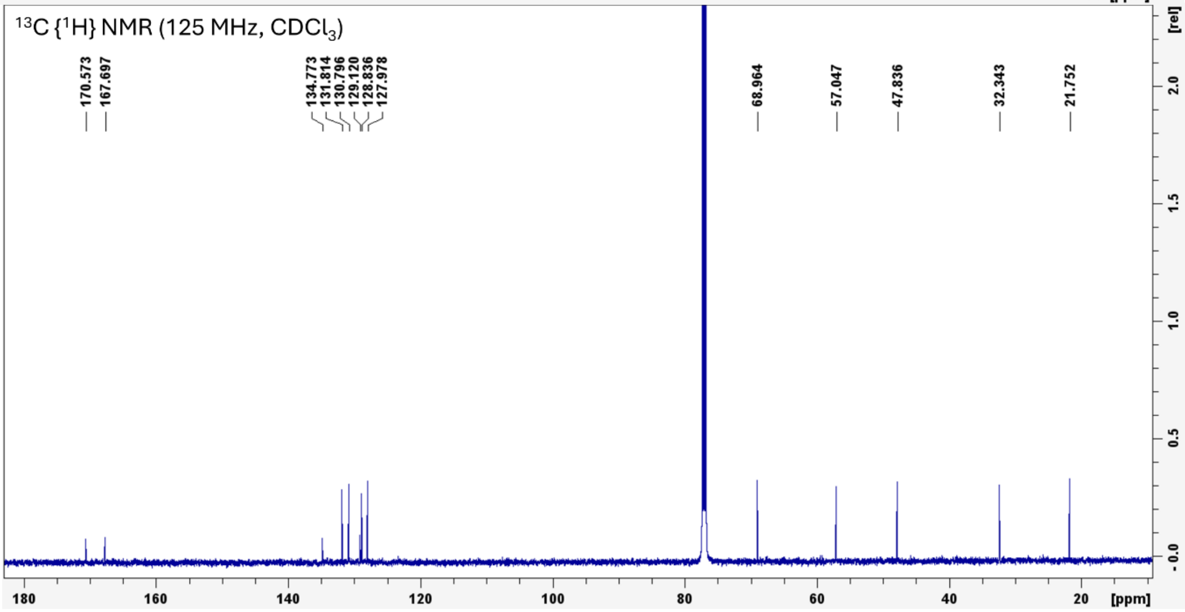
^{13}C {^{1}H} NMR (125 MHz, CDCl$_3$)
170.573
167.697
134.773
131.814
130.796
129.120
128.836
127.978
68.964
57.047
47.836
32.343
21.752
[ppm]
[rel]

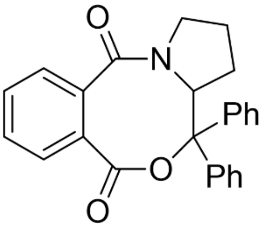

8i

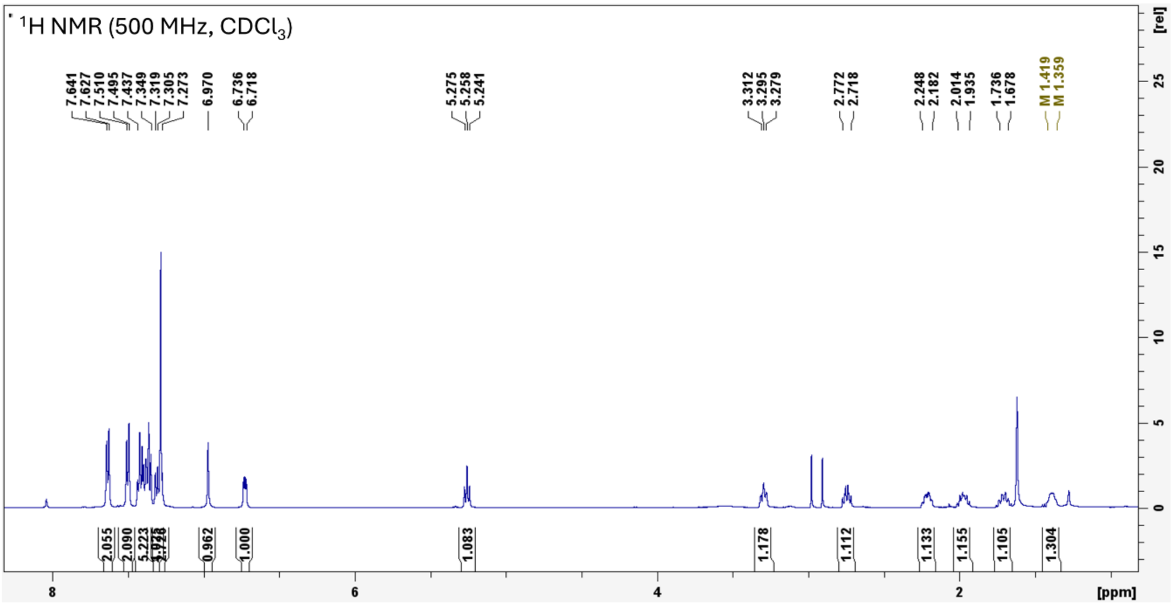

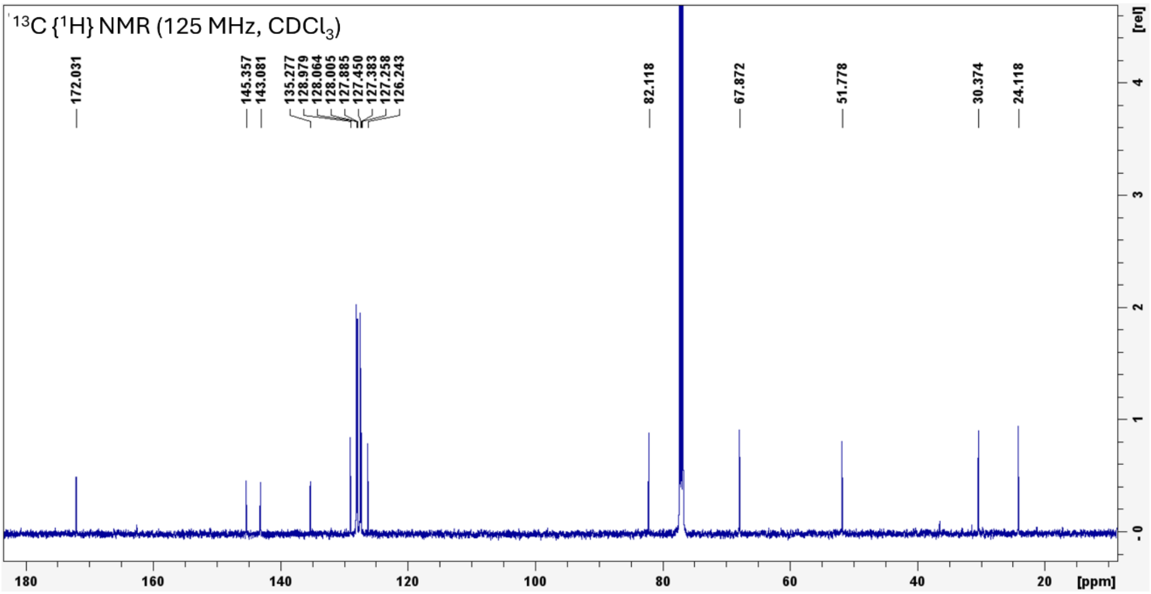

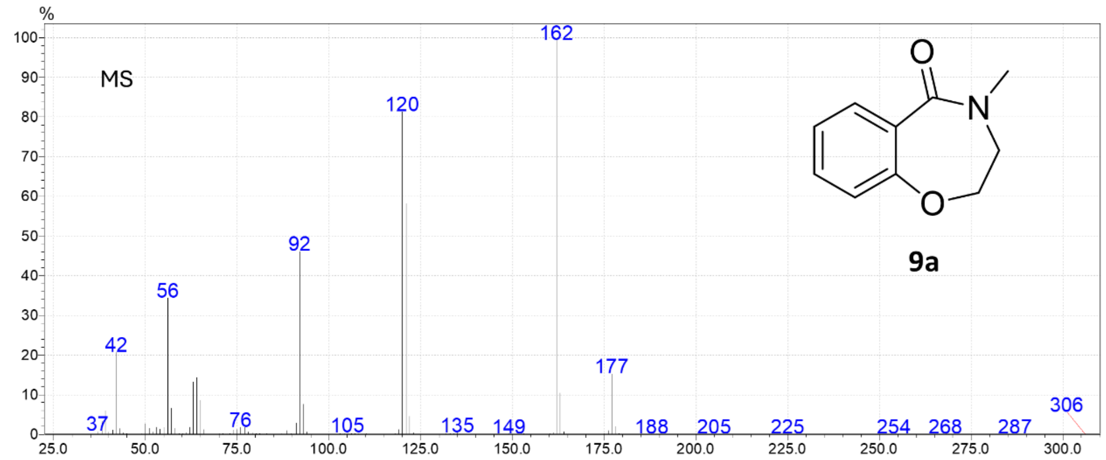
MS
162
120
92
56
42
177
306
9a

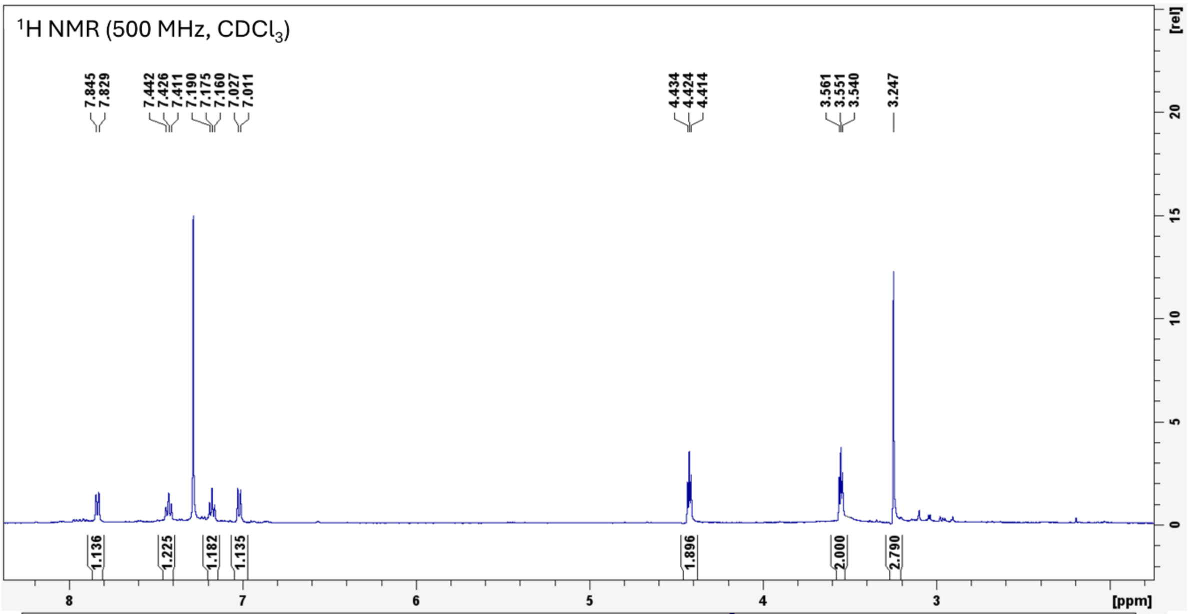
1H NMR (500 MHz, CDCl3)
7.845
7.829
7.442
7.426
7.411
7.190
7.175
7.160
7.027
7.011
4.434
4.424
4.414
3.561
3.551
3.540
3.247
1.136
1.225
1.182
1.135
1.896
2.000
2.790
[ppm]

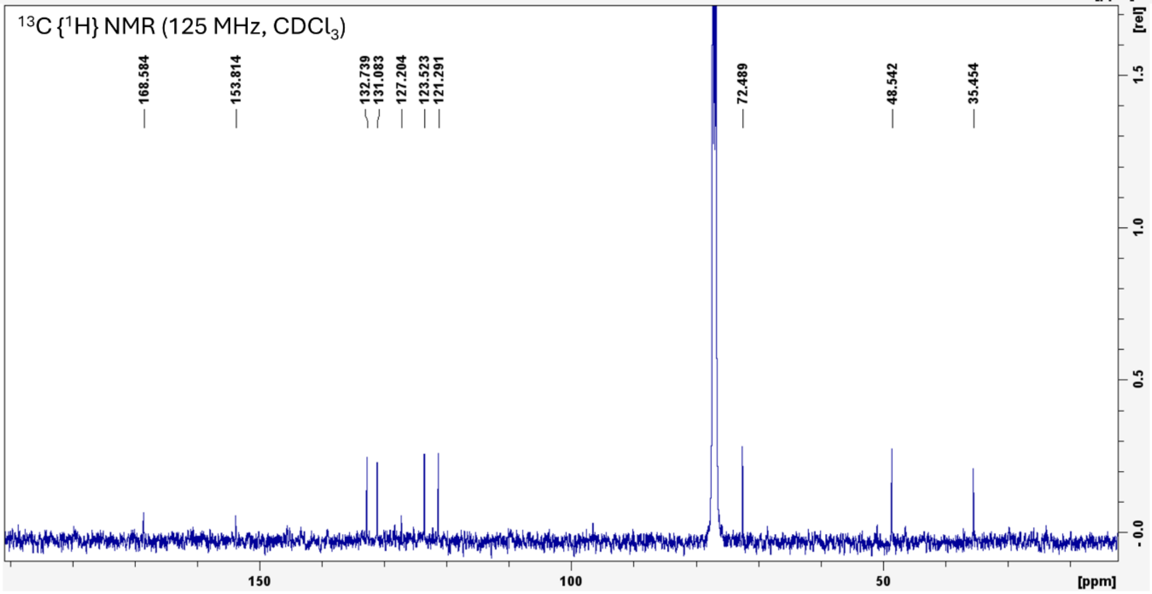
13C {1H} NMR (125 MHz, CDCl3)
168.584
153.814
132.739
131.083
127.204
123.523
121.291
72.489
48.542
35.454
[ppm]

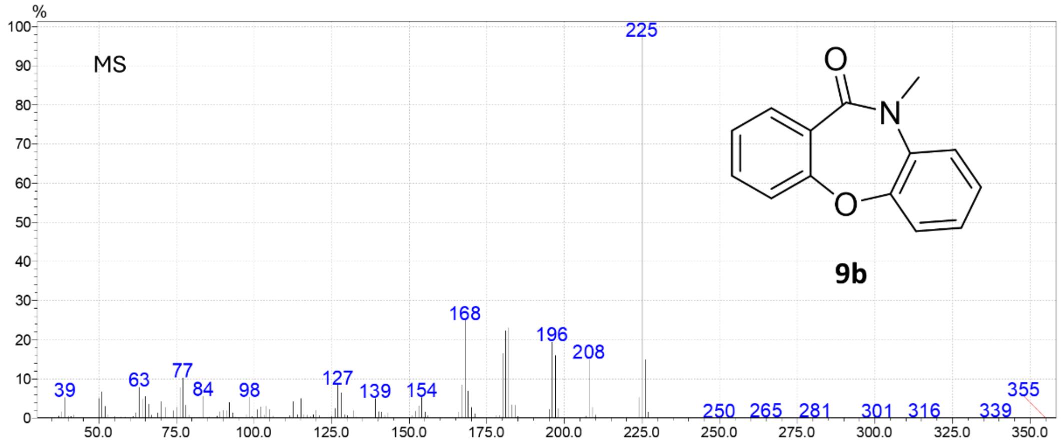
MS
9b
225
168
196
208
127
77
63
39
84
98
139
154
250
265
281
301
316
339
355

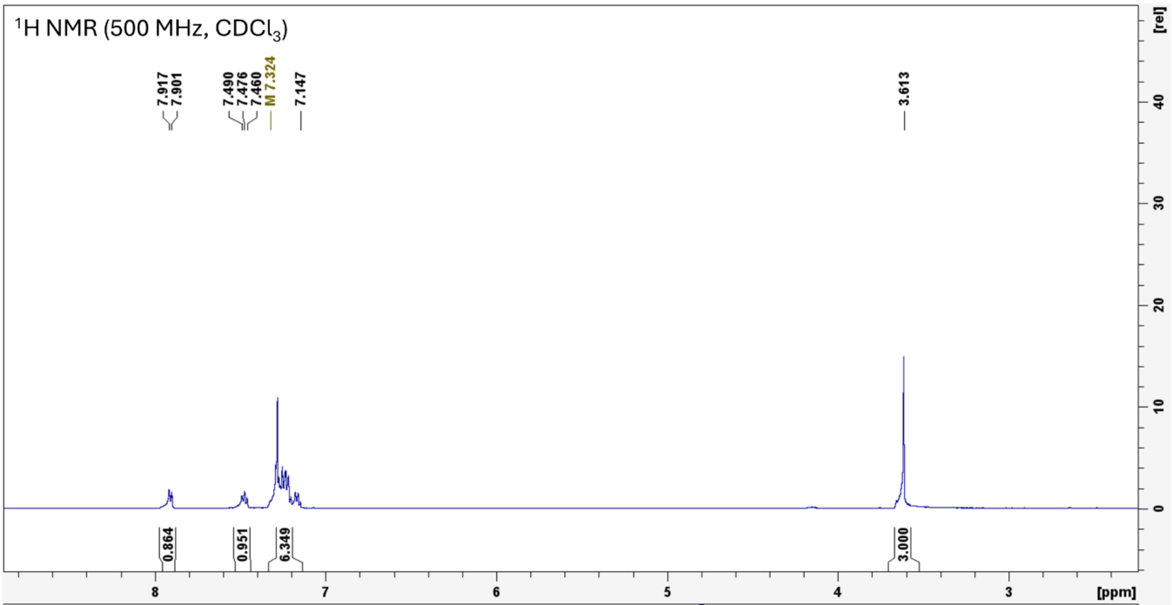
¹H NMR (500 MHz, CDCl₃)
7.917
7.901
7.490
7.476
7.460
M 7.324
7.147
3.613
0.864
0.951
6.349
3.000
[ppm]
[rel]

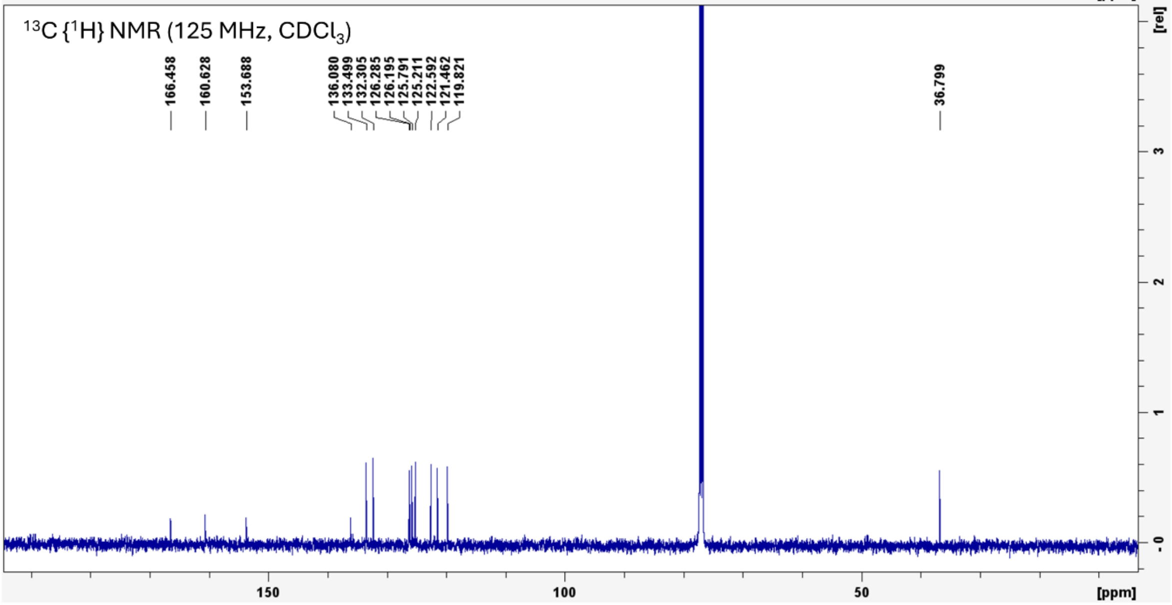
¹³C {¹H} NMR (125 MHz, CDCl₃)
166.458
160.628
153.688
136.080
133.499
132.305
126.285
126.195
125.791
125.211
122.592
121.462
119.821
36.799
[ppm]
[rel]
